# A Head-to-Head Comparison of Four Artemisinin-Based Combinations for Treating Uncomplicated Malaria in African Children: A Randomized Trial

**DOI:** 10.1371/journal.pmed.1001119

**Published:** 2011-11-08

**Authors:** The Four Artemisinin-Based Combinations (4ABC) Study Group

**Affiliations:** Walter and Eliza Hall Institute of Medical Research, Australia

## Abstract

**Background:**

Artemisinin-based combination therapies (ACTs) are the mainstay for the management of uncomplicated malaria cases. However, up-to-date data able to assist sub-Saharan African countries formulating appropriate antimalarial drug policies are scarce.

**Methods and Findings:**

Between 9 July 2007 and 19 June 2009, a randomized, non-inferiority (10% difference threshold in efficacy at day 28) clinical trial was carried out at 12 sites in seven sub-Saharan African countries. Each site compared three of four ACTs, namely amodiaquine-artesunate (ASAQ), dihydroartemisinin-piperaquine (DHAPQ), artemether-lumefantrine (AL), or chlorproguanil-dapsone-artesunate (CD+A). Overall, 4,116 children 6–59 mo old with uncomplicated *Plasmodium falciparum* malaria were treated (1,226 with AL, 1,002 with ASAQ, 413 with CD+A, and 1,475 with DHAPQ), actively followed up until day 28, and then passively followed up for the next 6 mo. At day 28, for the PCR-adjusted efficacy, non-inferiority was established for three pair-wise comparisons: DHAPQ (97.3%) versus AL (95.5%) (odds ratio [OR]: 0.59, 95% CI: 0.37–0.94); DHAPQ (97.6%) versus ASAQ (96.8%) (OR: 0.74, 95% CI: 0.41–1.34), and ASAQ (97.1%) versus AL (94.4%) (OR: 0.50, 95% CI: 0.28–0.92). For the PCR-unadjusted efficacy, AL was significantly less efficacious than DHAPQ (72.7% versus 89.5%) (OR: 0.27, 95% CI: 0.21–0.34) and ASAQ (66.2% versus 80.4%) (OR: 0.40, 95% CI: 0.30–0.53), while DHAPQ (92.2%) had higher efficacy than ASAQ (80.8%) but non-inferiority could not be excluded (OR: 0.35, 95% CI: 0.26–0.48). CD+A was significantly less efficacious than the other three treatments. Day 63 results were similar to those observed at day 28.

**Conclusions:**

This large head-to-head comparison of most currently available ACTs in sub-Saharan Africa showed that AL, ASAQ, and DHAPQ had excellent efficacy, up to day 63 post-treatment. The risk of recurrent infections was significantly lower for DHAPQ, followed by ASAQ and then AL, supporting the recent recommendation of considering DHAPQ as a valid option for the treatment of uncomplicated *P. falciparum* malaria.

**Trial Registration:**

ClinicalTrials.gov NCT00393679; Pan African Clinical Trials Registry PACTR2009010000911750

*Please see later in the article for the Editors' Summary*

## Abstract 2

The Four Artemisinin-Based Combinations (4ABC) Study Group reports a randomized, non-inferiority trial comparing the efficacy and safety of four ACTs in children with mild *Plasmodium falciparum* malaria from seven sub-Saharan African countries.

## Editors' Summary

**Background:**

Malaria is a global public-health problem. Half the world's population is at risk of this mosquito-borne parasitic disease, which kills a million people (mainly children living in sub-Saharan Africa) every year. Although several parasites cause malaria, *Plasmodium falciparum* is responsible for most of these deaths. During the second half of the 20th century, the main treatments for malaria were inexpensive “monotherapies” such as chloroquine and sulfadoxine-pyrimethamine. Unfortunately, the malaria parasite quickly developed resistance to many of these monotherapies, and in the 1990 s, there was a widespread upsurge in *P. falciparum* malaria. To combat this increase, the World Health Organization (WHO) now recommends artemisinin-based combination therapy (ACT) for first-line treatment of *P. falciparum* malaria in all regions where there is drug-resistant malaria. In ACT, artemisinin derivatives (new, fast-acting antimalarial drugs) are used in combination with another antimalarial drug (a partner drug) to reduce the chances of *P. falciparum* becoming resistant to either drug.

**Why Was This Study Done?:**

WHO currently recommends five ACTs—amodiaquine-artesunate (ASAQ), dihydroartemisinin-piperaquine (DHAPQ), artemether-lumefantrine (AL), artesunate-mefloquine, and artesunate-sulfadoxine-pyrimethamine—for the treatment of malaria. Its treatment guidelines state that the choice of ACT in a country or region should be based on the local level of resistance to the non-artemisinin-based partner drug in the combination. However, data on resistance levels to these partner drugs are scarce or unavailable for many sub-Saharan African countries. To help these countries make an informed choice about their national antimalarial treatment policies, in this randomized, non-inferiority trial, the researchers compare the efficacy and safety of four ACTs in African children with uncomplicated (mild) *P. falciparum* malaria. In a randomized trial, groups of randomly chosen patients with a specific disease are given different treatments and then followed to compare the outcomes of these interventions. A non-inferiority trial investigates whether one treatment is not worse than another treatment.

**What Did the Researchers Do and Find?:**

Each of twelve sites in seven sub-Saharan African countries compared three ACTs out of ASAQ, DHAPQ, AL, and chlorproguanil-dapsone-artesunate (CD+A). Overall, 4,116 young children with uncomplicated malaria were treated with ACT, actively followed up for 28 days (their parents brought them back to the site for pre-arranged check-ups), and passively followed up for six months (parents brought their children back if they developed any illnesses). At each visit, blood samples were examined for the presence of parasites, and a technique called PCR was used to determine which cases of malaria were new infections and which were recurrences of the original infection. The researchers then calculated the percentage of patients with no infection or with a new infection (the PCR-adjusted adequate clinical and parasitological response [ACPR]) and the percentage of patients with no infection (the PCR-unadjusted ACPR). For the PCR-adjusted efficacy, three pair-wise comparisons (DHAPQ versus AL, DHAPQ versus ASAQ, and ASAQ versus AL) showed non-inferiority at 28 days. That is, for example, similar percentages of patients given DHAPQ or AL (97.3% and 95.5%, respectively) had either no infection or a new infection. CD+A was less efficacious than the other three treatments. For the PCR-unadjusted efficacy, AL was significantly less efficacious than DHAPQ and ASAQ; DHAPQ had a higher efficacy than ASAQ, but non-inferiority could not be excluded. That is, the difference in efficacy of these two drugs might have happened by chance.

**What Do These Findings Mean?:**

These findings suggest that AL, ASAQ, and DHAPQ are all efficacious for the treatment of uncomplicated malaria in children; CD+A was withdrawn partway through the trial because of side effects, but these findings also suggest that it was less efficacious than the other ACTs. Importantly, the PCR-unadjusted results indicate that the risk of children becoming re-infected with malaria parasites soon after treatment was lowest for DHAPQ, followed by ASAQ, and then AL. Because these findings are based on pooled results from seven sub-Saharan African countries, they are likely to be generalizable and thus of use in setting national antimalarial drug policies throughout the region. AL and ASAQ are already included in the antimalarial drug policies of many sub-Saharan African countries, note the researchers, but these findings support the WHO recommendation that DHAPQ should also be considered for the treatment for uncomplicated *P. falciparum* malaria.

**Additional Information:**

Please access these websites via the online version of this summary at http://dx.doi.org/10.1371/journal.pmed.1001119.

- Information is available from WHO on malaria (in several languages); the 2010 World Malaria Report provides details of the current global malaria situation; the WHO Guidelines for the Treatment of Malaria and the report Assessment and Monitoring of Antimalarial Drug Efficacy for the Treatment of Uncomplicated Malaria are available
- The US Centers for Disease Control and Prevention provide information on malaria (in English and Spanish), including a selection of personal stories about malaria
- Information is available from the Roll Back Malaria Partnership on the global control of malaria including fact sheets about ACTs and about malaria in Africa
- MedlinePlus provides links to additional information on malaria (in English and Spanish)

## Introduction

The burden of malaria has declined substantially in several areas of sub-Saharan Africa, particularly in the past 3–5 y [1]. Such a change has been attributed to a combination of factors [2], including large scale indoor residual spraying campaigns [3],[4], massive distribution of insecticide-treated bed nets [5], and the introduction of artemisinin-based combination treatments (ACTs) [6],[7]. The scale-up of the interventions has been possible thanks to the availability of more funding, especially from the Global Fund to Fight AIDS, Tuberculosis and Malaria [8], that has allowed an increasing number of countries to include ACTs in their national treatment guidelines as first and, in some cases, second-line treatments, and to the massive scale-up implementation of treatment programs [9]. In addition, increased funding for research, often through effective public–private partnerships [10], has resulted in the availability of several ACTs [11]. However, data to guide individual countries in choosing the most appropriate ACTs are limited. The World Health Organization (WHO), which recently produced revised guidelines for the treatment of malaria, states that the choice of ACT in a country or region should be based on the level of resistance to the medicine partnered to the artemisinin derivative in the combination [12]. However, up-to-date treatment efficacy data for the partner medicine to the artemisinin derivative are scarce.

The WHO recommends five ACTs, namely artemether-lumefantrine (AL), amodiaquine-artesunate (ASAQ), mefloquine-artesunate, sulfadoxine-pyrimethamine-artesunate, and, most recently included, dihydroartemisinin-piperaquine (DHAPQ) [12]. Each of these combinations may have different advantages and disadvantages that vary according to a number of factors, including malaria endemicity, safety, tolerability, dosing, post-treatment prophylactic effect, resistance to the partner drug of the prevailing parasites in the area, and price. Accordingly, we carried out a head-to-head comparison of the safety and efficacy of several ACTs, with the aim of providing the information necessary to make an informed choice for the formulation of relevant national antimalarial treatment policies. The ACTs tested included three of those recommended by the WHO, namely AL, ASAQ, DHAPQ, and one that was under development, chlorproguanil-dapsone-artesunate (CD+A).

AL was the first co-formulated ACT to become available and, together with ASAQ, is the most common ACT used in sub-Saharan Africa. DHAPQ has been recently submitted for registration to the European Medicines Agency under the orphan drug legislation [13] following two phase III trials that were carried out in Africa [14] and Asia [15]. DHAPQ is currently used as a recommended treatment only in Asia [16], and a formulation approved by a stringent drug regulatory authority, such as the European Medicine Agency, or pre-qualified by the WHO is not yet available. At the time our trial started, combining chlorproguanil-dapsone (Lapdap, GlaxoSmithKline) with artesunate (CD+A) was considered to be a promising combination. Lapdap was on the market until 2008, when the producer withdrew it following the results of several studies showing that it caused significant reductions in hemoglobin (Hb) levels in patients with glucose-6-phosphate dehydrogenase deficiency [17]. Therefore, the CD+A arm was stopped (because the drug was no longer in development owing to concerns over safety), and our trial continued with the other three ACTs under evaluation.

## Methods

### Study Design, Sites, and Concealment of Patient Allocation

Between 9 July 2007 and 19 June 2009, a randomized, open-label, multicenter, non-inferiority clinical trial was carried out at 12 sites located in seven African countries (Nanoro, Burkina Faso; Fougamou and Lambaréné, Gabon; Afokang and Pamol, Nigeria; Mashesha and Rukara, Rwanda; Jinja, Tororo, and Mbarara, Uganda; Ndola, Zambia; and Manhiça, Mozambique). See protocol (Text S1) and amendments (Texts S3–S5), and CONSORT checklist (Text S2).

Each site compared three of the four ACTs under investigation, ASAQ, DHAPQ, AL, or CD+A. The decision of which treatments to test at a given site was made by considering the current first-line treatments, the known antimalarial resistance profile, and local malaria endemicity (Table 1). Patients were individually randomized according to a 1∶1∶1 scheme, with six sites testing ASAQ versus DHAPQ versus AL, four testing DHAPQ versus CD+A versus AL, and two testing ASAQ versus CD+A versus DHAPQ (Table 1). A randomization list was produced for each recruiting site by the National Institute for Health Research Medicines for Children Research Network Clinical Trials Unit, University of Liverpool, UK, with each treatment allocation concealed in opaque sealed envelopes that were opened only after the patient's recruitment.

**Table 1:** Study treatment to be tested by country.

Country	Sites	Transmission (Entomological Inoculation Rate)	Percent with Chloroquine Resistance	Percent with Sulfadoxine-Pyrimethamine Resistance	Study Treatments		
Burkina Faso	Nanoro	Seasonal, high (50–60)[46]	24 [46]	4 [46]	ASAQ	DHAPQ	AL
Gabon	Fougamou, Lambaréné	Perennial, high (50)	100 [47]	23 [48]	ASAQ	DHAPQ	AL
Nigeria	Afokang, Pamol	Perennial, high	45 [49]	30 [49]	ASAQ	DHAPQ	AL
Zambia	Ndola	Seasonal, mesoendemic	High	19 (in adults) [50]	ASAQ	DHAPQ	AL
Rwanda	Rukara	Seasonal, high	40 [51]	36 [51]	DHAPQ	CD+A	AL
Rwanda	Mashesha	Seasonal, high	50 [51]	12 [51]	DHAPQ	CD+A	AL
Uganda	Jinja	Perennial, low (6) [34]	28 [52],[53]	49 [52],[53]	DHAPQ	CD+A	AL
Uganda	Tororo	Perennial (>563) [34]	45 [52],[53]	9–15 [52],[53]	DHAPQ	CD+A	AL
Mozambique	Manhiça	Perennial, mesoendemic [54]	78 [55]	22 [55]	ASAQ	CD+A	DHAPQ
Uganda	Mbarara	Mesoendemic	81 [56]	25 [56]	ASAQ	CD+A	DHAPQ

Entomological inoculation rate is infective bites/person/year.

Children 6–59 mo old (12–59 mo old at sites where CD+A was used) attending the health facilities with suspected uncomplicated malaria were included in the study if they fulfilled all the following inclusion criteria: body weight >5 kg, microscopically confirmed *Plasmodium falciparum* mono-infection with asexual parasite densities between 2,000 and 200,000/µl, fever (axillary temperature ≥37.5°C) or history of fever in the preceding 24 h, and Hb ≥7.0 g/dl. Patients were not recruited if they met at least one of the following exclusion criteria: participation in any other investigational drug study during the previous 30 d; known hypersensitivity to the study drugs; severe malaria [18] or other danger signs, e.g., not able to drink or breast-feed, vomiting (more than twice in 24 h), recent history of convulsions (more than once in 24 h), unconscious state, or unable to sit or stand; severe malnutrition (weight for height <70% of the median National Center for Health Statistics/WHO reference) or any other concomitant illness or underlying disease, including known glucose-6-phosphate dehydrogenase deficiency; contra-indication to receive the trial drugs; or ongoing prophylaxis with drugs having antimalarial activity. Patients satisfying these eligibility criteria were enrolled if the parent/guardian gave informed consent to participate and signed the corresponding form.

Three treatments were co-formulated (ASAQ, DHAPQ, and AL), while CD+A consisted of separate tablets of chlorproguanil-dapsone and artesunate. All drugs were administered under direct supervision during three consecutive days, according to the patient's body weight. ASAQ (Coarsucam, Sanofi Aventis) was given once daily, at the standard dose of 2.8–5.5 mg/kg and 7.5–15 mg/kg of artesunate and amodiaquine, respectively. Three formulations were used (25 mg artesunate +67.5 mg amodiaquine; 50 mg artesunate +135 mg amodiaquine; and 100 mg artesunate +270 mg amodiaquine) and given at the dosage of one tablet/day according to body weight (<9 kg, 9–17.9 kg, and 18–35.9 kg, respectively). DHAPQ (Eurartesim, Sigma-Tau) was given once daily, at the standard dosage of 2.25 mg/kg and 18 mg/kg of dihydroartemisinin and piperaquine, respectively, rounded up to the nearest half tablet. Two formulations were used (20 mg dihydroartemisinin +160 mg piperaquine and 40 mg dihydroartemisinin +320 mg piperaquine). AL (Coartem, Novartis) was administered twice a day (at enrollment and at 8, 24, 36, 48, and 60 h) according to the following dosage: weight = 5–14 kg: one tablet per dose; weight = 15–24 kg: two tablets per dose; weight = 25–34 kg: three tablets per dose. Chlorproguanil-dapsone (Lapdap pediatric tablets, GlaxoSmithKline) was administered once a day at the dose of 2.0 mg/kg chlorproguanil and 2.5 mg/kg dapsone. The daily dose of the formulation used (15 mg chlorproguanil and 18.75 mg dapsone) was given according to body weight, i.e., 4–5.9 kg: 0.5 tablet; 6–9.9 kg: 1 tablet; 10–13.9 kg: 1.5 tablet; 14–15.9 kg: 2 tablets; 16–19.9 kg: 2.5 tablets; 20–24.9 kg: 3 tablets; 25–30.9 kg: 4 tablets. Arsumax (Guilin Pharmaceutical for Sanofi-Synthelabo) contained 50 mg artesunate per tablet and was administered with chlorproguanil-dapsone according to the following dosage: 5–8.3 kg: 0.5 tablet; 8.4–16.7 kg: 1 tablet; 16.8–20.8 kg: 1.5 tablet; 20.9–29.2 kg: 2 tablets. In case of vomiting, a full dose was repeated if this occurred within 30 min, or half a dose if it occurred between 30 min and 1 h. AL was administered concomitantly with a fatty meal (as recommended by the manufacturer), e.g., milk or groundnuts, while for the other three treatments no specific instructions regarding co-administration with food were given. For infants, medicines were crushed, mixed with water, and administered as a slurry. Treatment was administered by a study nurse, with the clinician or other staff following up the patient and assessing the end points blinded to the treatment assignment whenever possible.

### Treatment Follow-Up and Clinical and Laboratory Procedures

Treatment was directly observed and children stayed at the health facility at least 1 h to check for any vomiting. At some sites children were kept at the health facility for the whole 3-d dosing period. The parent/guardian was asked to return with the child for scheduled visits on days 3, 7, 14, 21, and 28, or if any symptoms occurred (active follow-up period). Field workers traced patients who missed any visit. In addition, after day 28 and for the next 6 mo, the parent/guardian was encouraged to attend the health facility whenever the child was sick, so as to detect any malaria episode(s) (passive follow-up period). In case of clinical malaria, the child received the same treatment as for the primary episode and was actively followed up for the following 28 d (unpublished data). For each visit, a physical examination was performed by the study clinicians, vital signs were recorded, and axillary temperature was measured with an electronic thermometer. Adverse events (AEs) and serious adverse events (SAEs) were recorded and monitored throughout the study. Rescue treatment for recurrent infections identified during the 28-d follow-up, as well as for severe malaria, was according to local national guidelines.

Capillary or venous blood was taken at every visit. Thick and thin blood films were prepared, dried, and Giemsa-stained, and parasite density estimated by counting the number of asexual parasites in 200 white blood cells, assuming a standard white blood cell count of 8,000/µl. Quality control was performed in blind conditions on 10% of all the slides. Samples for hematology (full blood count) were taken at enrollment and at days 3, 7, 14, and 28, while biochemistry (liver and renal functions) was performed at enrollment and days 7 and 28. Laboratory tests were also performed at any other visit if judged necessary by the clinician. For PCR analysis, blood samples were collected on filter paper (Whatman 3MM) at enrollment and at any visit after day 7. Each filter paper was dried and individually stored in a plastic bag containing silica gel. All filter papers were subsequently transported to the Institute of Tropical Medicine, Antwerp, Belgium, where centralized genotyping according to international recommendations was conducted [19]. Briefly, purification of DNA was conducted as previously described [20] and three polymorphic genetic markers were genotyped sequentially, starting with GluRP, followed by MSP2, and ending with MSP1. Capillary electrophoresis was used for MSP2. Whenever a genetic marker showed a new infection, i.e., no common allele between day of recurrent infection and day 0, this was taken as the final result and the analysis was stopped. For samples showing a recurrent infection, i.e., at least one identical allele between day 0 and day of recurrent infection, the analysis was carried out until MSP1. If the latter showed also at least one identical allele between day of recurrent infection and day 0, then the infection was classified as a recrudescence [19]. All results were double read and discrepancies resolved.

### Outcome Classification

Treatment outcomes were classified according to the WHO guidelines [21], as early treatment failure, late clinical failure, late parasitological failure, and adequate clinical and parasitological response (ACPR). An AE was defined as any untoward medical occurrence, irrespective of its suspected relationship to the study medication, in accordance with the International Conference of Harmonization guidelines, and graded as mild, moderate, severe, or life-threatening.

The primary end points were PCR-adjusted and unadjusted ACPR at day 28; secondary efficacy outcomes included PCR-adjusted and unadjusted ACPR at day 63 (day 28 plus days 29–63, the latter with passive detection of clinical malaria cases) [22], parasite and fever clearance times, presence and clearance of gametocytes, Hb changes from baseline to days 3, 7, 14, and 28, and safety profiles. All standard safety outcomes, such as incidence of AEs, changes from baseline on hematology and clinical chemistry parameters, and vital sign variations during the study were evaluated. PCR-unadjusted ACPR included as treatment successes only patients classified as ACPR at the end of the follow-up period while PCR-adjusted ACPR also included as successes patients classified as either late clinical failure or late parasitological failure for whom the recurrent infection was identified by genotyping as a new infection. An independent end point adjudication committee comprising three individuals (not authors or investigators in the trial) blind to both treatment allocation and PCR results reviewed records with discrepancies between the outcome registered by the investigators and the information collected during follow-up and assigned a final outcome. The end point adjudication committee also reviewed the patients lost to follow-up or withdrawn, to determine any possible relation with the study drug or malaria.

### Ethical Considerations and Patient Safety

The study protocol and successive amendments were approved by the Institutional Review Board of the Institute of Tropical Medicine, Antwerp; the Ethical Committee of the Antwerp University Hospital; the national Ethics Review Committee or Institutional Review Board at each trial site; and the national competent authorities, as appropriate, e.g., Ministry of Health and national drug regulatory authorities. The trial was conducted under the provisions of the Declaration of Helsinki and in accordance with Good Clinical Practices guidelines set up by the WHO and by the International Conference on Harmonization. An independent Data Safety and Monitoring Committee was created prior to the beginning of the trial and regularly reviewed both the safety data and the quality of the information collected. The trial was registered prior to the enrollment of the first patient in the ClinicalTrials.gov registry (NCT00393679, http://clinicaltrial.gov/ct2/show/NCT00393679) and in the Pan African Clinical Trials Registry (/PACTR2009010000911750, http://www.pactr.org/).

### Statistical Analysis

A Statistical Analysis Plan was finalized before data extraction, and statistical analysis was performed accordingly. For the primary outcome, four analysis approaches were adopted: intention to treat (ITT), per protocol, and two sensitivity analyses. Patients lost to follow-up or withdrawn for reasons unrelated to malaria, as determined by the end point adjudication committee, were excluded from the ITT analysis. Major protocol violators defined prior to analysis (no blood slide or parasite density >200,000/µl at day 0, no fever or history of fever and parasite density <2,000/µl, Hb at day 0<7.0 g/dl, signed informed consent not available, any of the exclusion criteria at day 0 except known hypersensitivity to the study drug, study drug either not taken or <80% of the dose taken, administration of a treatment with antimalarial activity during the follow-up) were excluded from the per protocol analysis. All secondary outcomes were analyzed using the ITT approach, with patients having received rescue treatment or lost to follow-up censored at their last visit. Because CD+A was discontinued partway through the study and the lower age limit for inclusion changed at that time, the analyses involving CD+A include only patients randomized prior the discontinuation.

The primary hypothesis was that the four treatments are clinically non-inferior to each other as measured by the proportion of children with PCR-adjusted and unadjusted ACPR at day 28. This was tested for each of all possible (six) pair-wise comparisons and the two primary end points, with the limit of non-inferiority on an odds ratio (OR) scale corresponding to a 10% absolute difference in ACPR. Data from sites testing the same two treatments were included and combined into a meta-analysis using the study site as unit.

The meta-analysis was performed using ORs rather than risk differences as the latter usually display more heterogeneity than ORs [23]. Nevertheless, for each site, we also report the risk differences and 95% confidence intervals (CIs) calculated using Wilson's score method, together with the non-inferiority limit [24]. Heterogeneity was examined by visually inspecting the OR and the 95% CI in a forest plot. Additionally, qualitative heterogeneity was declared if there was at least one site with a statistically significant treatment difference in one direction (CI for measure of effect entirely above point of no difference) and at least one site with a difference in the opposite direction (CI for measure of effect entirely below point of no difference). For this assessment, a clinical significance level of 10% (i.e., 90% CIs in the forest plot) was used. For each pair-wise comparison, provided no qualitative heterogeneity was present, the pooled OR and corresponding 95% CI were computed, with the treatment having the highest ACPR taken as control.

For the secondary outcomes, including ACPR at day 63, differences between treatments were estimated using differences in means, ORs, or hazard-ratios, as appropriate, and the corresponding 95% CIs. Parasite and fever clearance were defined as two consecutive days without parasite or fever, respectively, and analyzed using Cox proportional hazard models stratified by study site. Gametocyte prevalences were assessed by calculating the number of gametocyte carriage days for each patient as an area under the curve. The number of gametocyte carriage days was analyzed using zero-inflated Poisson models with the number of days in active follow-up as offset, and summarized as ORs (for zero gametocyte carriage days) and ratios of the expected number of gametocyte carriage days between groups. This last ratio is expressed as the percentage of the reduction in gametocyte carriage days.

Safety was assessed through AE reporting; all individuals having received at least one dose of the treatment were included and analyzed according to the treatment actually received.

### Sample Size Calculation

This study was designed as a non-inferiority trial, and on an expected ACPR at day 28 of at least 90%. Though the primary intention was to carry out a pooled analysis able to provide overall head-to-head comparisons between the different treatments, the sample size was based on the individual sites to also produce locally meaningful results. Therefore, for each individual site, 170 children per arm should be able to show that the difference in efficacy between treatments was less than 10%, at 5% significance level and 90% power, assuming equal true ACPR rates.

## Results

### Trial Profile and Baseline Characteristics

**Figure 1:**
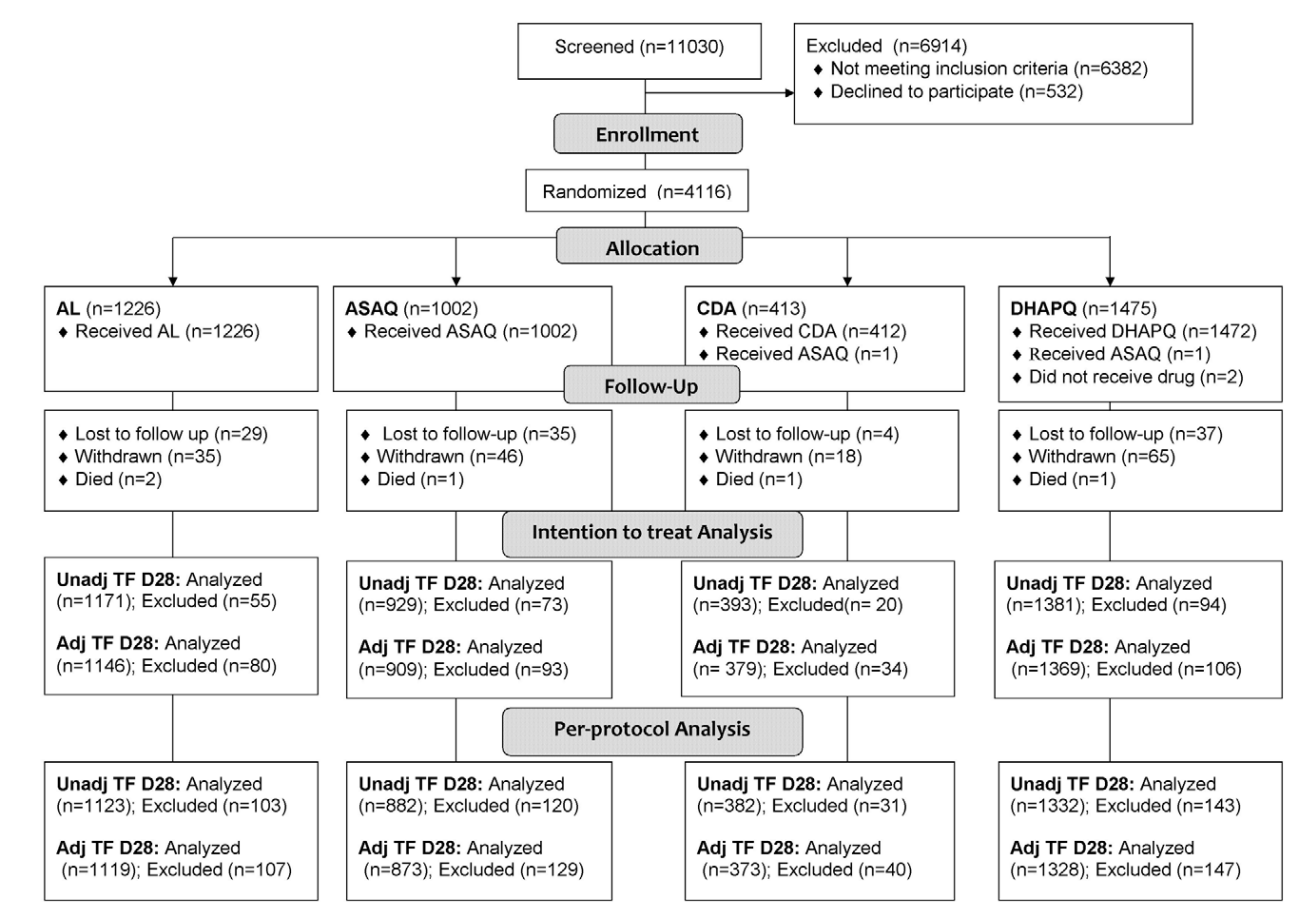
Trial profile up to day 28. Adj TF, adjusted treatment failure; Unadj TF, unadjusted treatment failure.

Overall, 11,030 patients were screened, 6,382 did not meet the inclusion/exclusion criteria, 532 declined to participate, and 4,116 were recruited and randomized to receive the study drugs (1,226 to AL, 1,002 to ASAQ, 413 to CD+A, and 1,475 to DHAPQ) (Figure 1). The smaller sample size in the CD+A group is explained by the decision of the manufacturer to stop the drug's development [17] and the subsequent closure of this treatment arm in February 2008. Therefore, at sites where CD+A was one of the study treatments, patients enrolled after 17 February 2008 were randomized to the remaining two study drugs and the lower age limit was changed from 12 to 6 mo. For the ITT analysis of the PCR-unadjusted ACPR at day 28, 3,874 patients were included, while 105 (2.6%) (35 for ASAQ, 37 for DHAPQ, 29 for AL, and 4 CD+A,) were lost to follow-up and 137 (3.3%) (38 for ASAQ, 57 for DHAPQ, 26 for AL, and 16 for CD+A) were withdrawn for reasons definitely or probably not related to malaria or treatment. In addition, for the PCR-adjusted ACPR (ITT population), 71 additional patients were excluded because of either indeterminate PCR results or unavailable blood sample (20 for ASAQ, 12 for DHAPQ, 25 for AL, and 14 for CD+A). Randomization generated comparable groups within countries and overall (Table 2).

**Table 2:** Baseline characteristics (ITT population).

Variable	Treatment Group			
	ASAQ (*n* = 1,002)	DHAPQ (*n* = 1,475)	AL (*n* = 1,226)	CD+A (*n* = 413)
Male/female: number (percent)	558/444 (55.7/44.3)	780/695 (52.9/47.1)	627/559 (51.1/48.9)	214/199 (51.8/48.2)
Age (mo), mean (SD)	30.2 (14.7)	30.2 (14.1)	29.5 (14.0)	30.7 (12.8)
Weight (kg), mean (SD)	11.4 (3.0)	11.4 (2.8)	11.1 (2.8)	11.6 (2.6)
Fever, *n* (percent)	611 (61.0)	918 (62.2)	704 (57.4)	244 (59.1)
Temperature (° C), mean (SD)	38.0 (1.3)	38.0 (1.2)	37.8 (1.2)	37.9 (1.3)
Parasite density (10^3^), median (IQR)	30 (10–65)	31 (10–68)	27 (10–58)	32 (12–65)
Presence of gametocytes, *n* (percent)	104 (10.4)	166 (11.3)	94 (7.7)	68 (16.5)
Hb (g/dl), mean (SD)	9.1 (1.4)	9.4 (1.5)	9.2 (1.5)	9.7 (1.4)
Leucocytes (10^9^/l), mean (SD)	9.7 (3.9)	9.8 (3.8)	9.5 (3.9)	9.8 (3.8)
Splenomegaly, *n* (percent)	49 (4.9)	88 (6.0)	80 (6.5)	27 (6.5)
Hepatomegaly, *n* (percent)	6 (0.6)	5 (0.3)	8 (0.7)	1 (0.2)
ALAT (IU/l), median (IQR)	23 (16–33)	23 (17–33)	24 (16–33)	25 (19–36)
Bilirubin (µmol/l), median (IQR)	15 (10–24)	15 (9–24)	14 (9–21)	16 (8–27)
Creatinine (µmol/l), median (IQR)	38 (31–44)	44 (34–58)	45 (35–62)	40 (31–60)

ALAT, alanine aminotransferase; IQR, interquartile range; SD, standard deviation.

The safety analysis included all other randomized patients apart from eight patients: two (randomized to DHAPQ) were withdrawn before receiving the study drug (one because of violation of entry criteria, one withdrawal of consent) and six were withdrawn because they vomited their first dose twice (four for DHAPQ, one for AL, and one for CD+A).

### Efficacy Results

**Figure 2:**
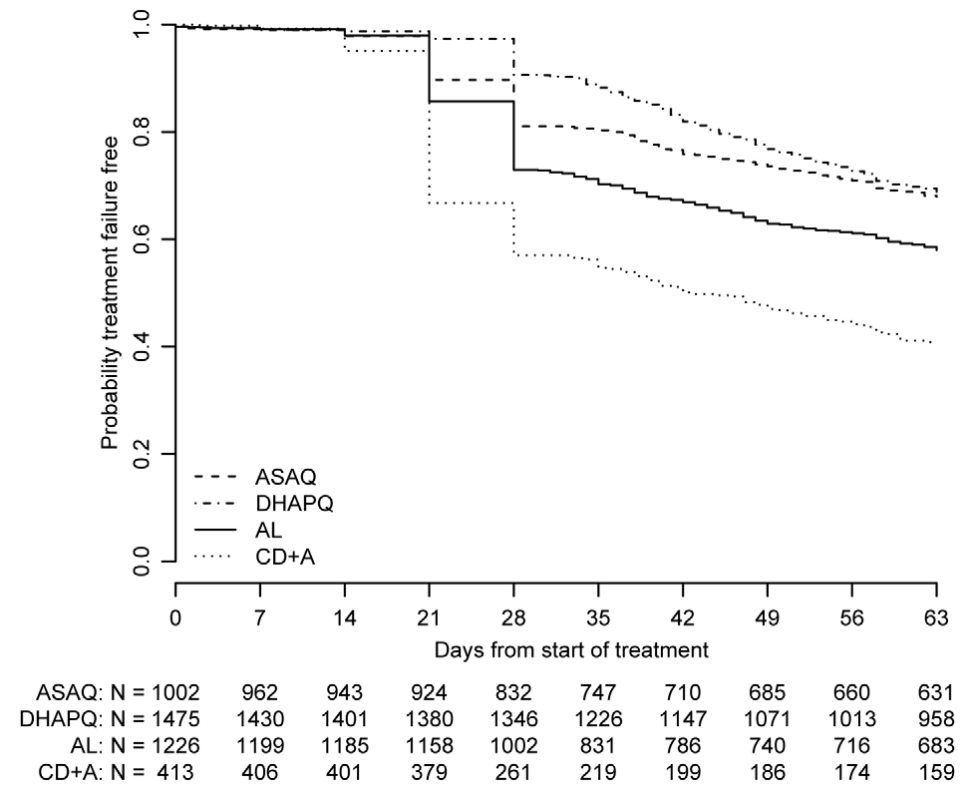
Proportion of patients whose treatment was failure-free by day of follow-up. ITT population; data pooled over all sites.

**Figure 3:**
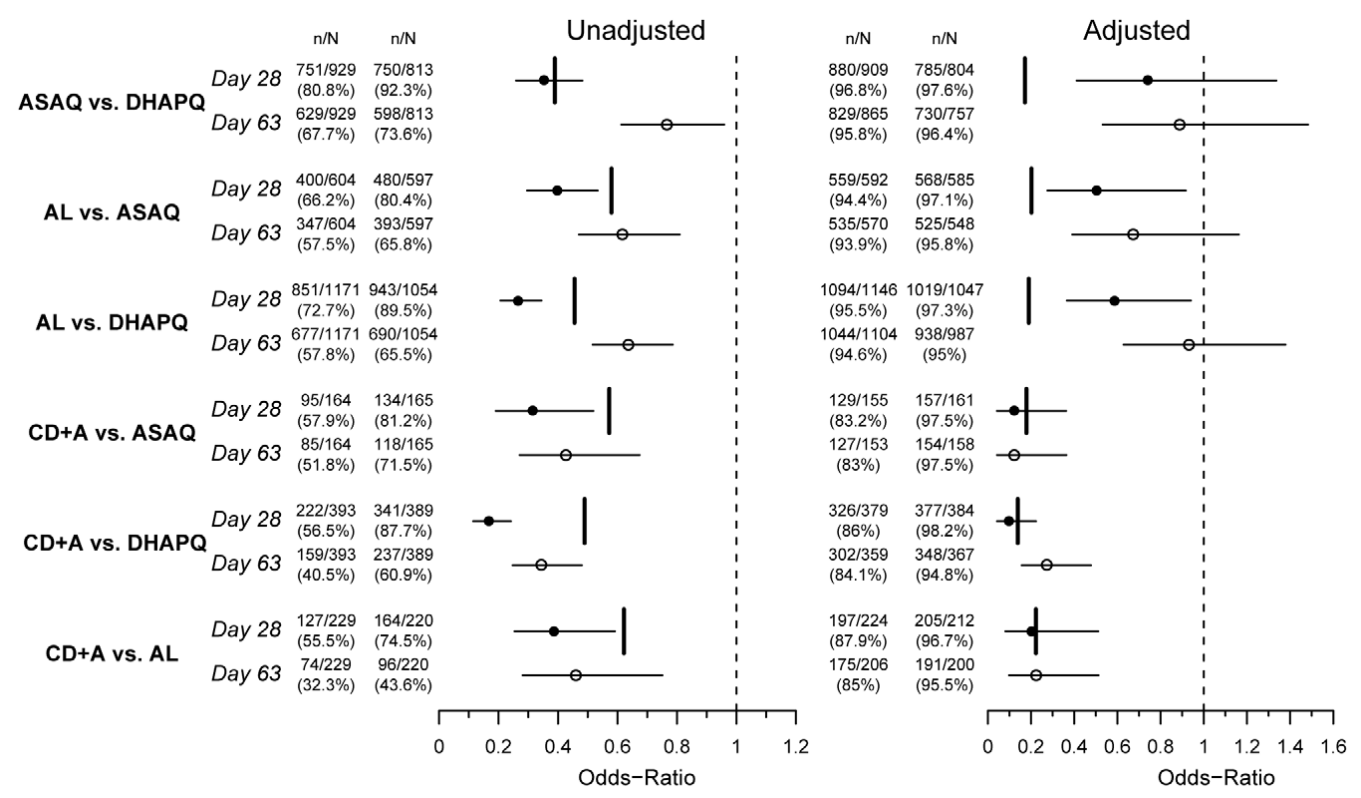
Six pair-wise comparisons at day 28 and day 63. OR (circles), 95% CI (horizontal bars), and non-inferiority limit (vertical bars) for each pair-wise analysis for PCR-unadjusted (left panel) and -adjusted (right panel) ACPR at days 28 (filled circles) and 63 (open circles) (ITT population). An OR and 95% CI<1 indicate a significantly higher efficacy of the second versus the first treatment of the pair-wise comparison. The non-inferiority limits (day 28 only) correspond to a 10% difference in efficacy, recalculated to an OR scale. Non-inferiority is established if the 95% CI lies completely above the non-inferiority limit.

All results presented below refer to the ITT analysis unless specified otherwise. As an indication of the overall efficacy of the different treatments, the pooled results are reported below. However, the analysis of each of the six pair-wise comparisons was stratified by site. The pooled (all sites) PCR-adjusted ACPR was high, around 95%, for ASAQ, DHAPQ, and AL, both at day 28 and 63, while for CD+A this was around 85%. In addition, CD+A had the lowest PCR-unadjusted ACPR, followed by AL, ASAQ, and then DHAPQ, with the majority of recurrences due to new infections (Table 3). At day 63, DHAPQ and ASAQ had similar PCR-unadjusted ACPR, though for the latter, recurrent infections occurred earlier (Figure 2). None of the pair-wise comparisons showed important qualitative heterogeneity. For the PCR-adjusted ACPR at day 28, non-inferiority could be established for the following pair-wise comparisons: AL versus DHAPQ (95.5% [1,094/1,146] versus 97.3% [1,019/1,047]; OR: 0.59, 95% CI: 0.37–0.94), ASAQ versus DHAPQ (96.8% [880/909] versus 97.6% [785/804]; OR: 0.74, 95% CI: 0.41–1.34), and AL versus ASAQ (94.4% [559/592] versus 97.1% [568/585]; OR: 0.50, 95% CI: 0.28–0.92). CD+A had a significantly lower efficacy than the other ACTs, but non-inferiority could not be ruled out (Figure 3). For the day 28 PCR-unadjusted ACPR, none of the pair-wise comparisons could establish the predefined non-inferiority (Figure 3). AL was significantly less efficacious than DHAPQ (72.7% [851/1171] versus 89.5% [943/1054]; OR: 0.27, 95% CI: 0.21–0.34) and ASAQ (66.2% [400/604] versus 80.4% [480/597]; OR: 0.40, 95% CI: 0.30–0.53), while in the comparison between ASAQ and DHAPQ, the latter had a higher efficacy, but non-inferiority could not be excluded (80.8% [751/929] versus 92.3% [750/813]; OR: 0.35, 95% CI: 0.26–0.48) (Figure 3). DHAPQ, ASAQ, and AL were significantly more efficacious than CD+A, with the 95% CI well below the limit of non-inferiority (Figure 3). The analyses in the per protocol population and all sensitivity analyses confirmed the robustness of these results (data not shown).

**Table 3:** Treatment outcomes by treatment.

Outcome	Details	ASAQ (*n* = 1,002)	DHAPQ (*n* = 1,475)	AL (*n* = 1,226)	CD+A (*n* = 413)
**Excluded from ITT analysis, percent (** ***n*** **)**	Withdrawal unrelated to study treatment or malaria	3.8 (38)	3.9 (57)	2.1 (26)	3.9 (16)
	Lost to follow-up	3.5 (35)	2.5 (37)	2.4 (29)	1.0 (4)
	Day 28: no PCR results	2.0 (20)	0.8 (12)	2.0 (25)	3.4 (14)
	Day 63: no PCR results	6.4 (64)	5.6 (83)	5.5 (67)	8.2 (34)
**ACPR, percent (** ***n/N*** **)**	Day 28: PCR unadjusted	80.8 (751/929)	90.5 (1,250/1,381)	72.7 (851/1,171)	56.5 (222/393)
	Day 28: PCR adjusted	96.8 (880/909)	97.6 (1,336/1,369)	95.5 (1,094/1,146)	86.0 (326/379)
	Day 63: PCR unadjusted	67.7 (629/929)	68.8 (950/1,381)	57.8 (677/1,171)	40.5 (159/393)
	Day 63: PCR adjusted	95.8 (829/865)	95.7 (1,242/1,298)	94.6 (1,044/1,104)	84.1 (302/359)
**Treatment failures, ** ***n***	ETF	2	2	0	1
	LTF (day 28): new infections	149	98	268	118
	LTF (day 28): recrudescences	18	22	41	49
	Deaths (up to day 63)	1	1	3	1
	Withdrawal related to treatment or malaria	8	8	9	2
	LTF (day 29–63): new infections	115	277	166	59
	LTF (day 29–63): recrudescences	7	23	8	4

ITT population; results pooled from all sites.

ETF, early treatment failure; LTF, late treatment failure (includes late parasitological and clinical failures; after day 28, only late clinical failures were detected).

**Figure 4:**
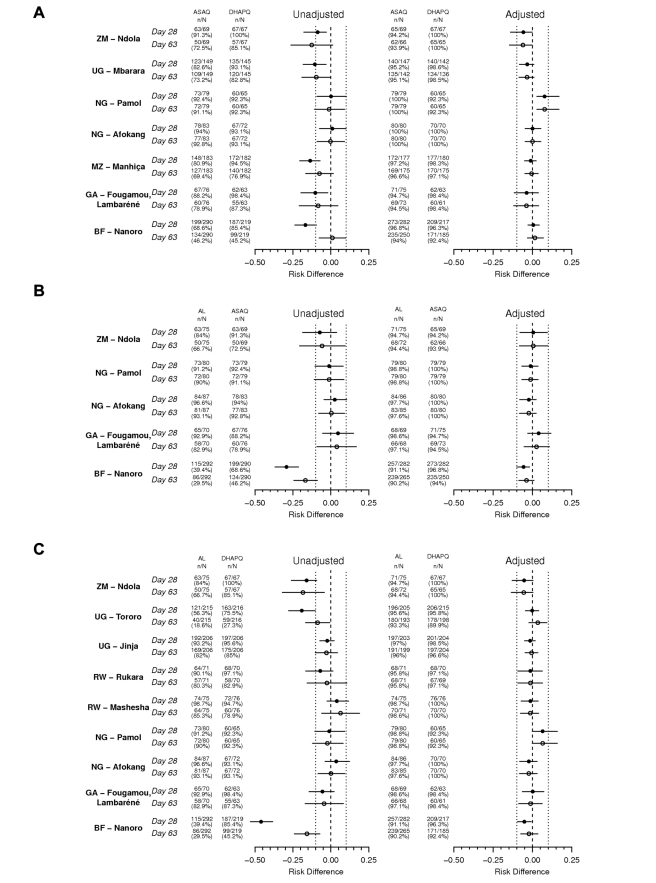
Treatment efficacy by pair-wise comparison and by site. Risk difference and 95% CI for three pair-wise analyses by site for PCR-unadjusted (left panel) and -adjusted (right panel) ACPR at days 28 (filled circles) and 63 (open circles) (ITT population) (A) ASAQ versus DHAPQ; (B) AL versus ASAQ; (C) AL versus DHAPQ. BF, Burkina Faso; GA, Gabon; MZ, Mozambique; NG, Nigeria; RW, Rwanda; UG, Uganda; ZM, Zimbabwe.

Day 63 results were similar to those observed at day 28. For the PCR-corrected efficacy, DHAPQ, ASAQ, and AL had high (≥94.6%) and similar efficacy. CD+A, in each pair-wise comparison, had a significantly lower efficacy (<85%) than any of the other three treatments. PCR-unadjusted efficacy was significantly higher for DHAPQ than for ASAQ (73.6% [598/813] versus 67.7% [629/929]; OR: 0.77, 95% CI: 0.61–0.96) and AL (65.5% [690/1054] versus 57.8% [677/1171]; OR: 0.64, 95% CI: 0.52–0.79), while that for ASAQ was significantly higher than for AL (65.8% [393/597] versus 57.5% [347/604]; OR: 0.62, 95% CI: 0.47–0.81). CD+A had a significantly lower efficacy than the other three treatments (Figure 3). Pair-wise comparisons of ACPR by site, both PCR-adjusted and unadjusted, followed a similar trend (Figure 4). However, when looking at the individual sites, PCR-unadjusted efficacy of DHAPQ as compared to AL was significantly higher in Ndola (Zambia), Tororo (Uganda), and Nanoro (Burkina Faso), while at the other sites it was similar or the difference did not reach statistical significance (Figure 4C); for the comparison with ASAQ, DHAPQ performed significantly better at most sites, with the exception of Pamol and Afokang (Nigeria) (Figure 4A). In addition, the PCR-unadjusted efficacy of ASAQ was significantly higher than that of AL in Nanoro (Burkina Faso), while at the other sites the risk difference was close to zero (Figure 4B).

DHAPQ had a PCR-adjusted efficacy similar to that of AL at most sites, with the exception of Pamol (Nigeria), where AL efficacy both at day 28 and 63 tended to be higher, and in Nanoro (Burkina Faso), with a significantly higher efficacy of DHAPQ at day 28 but not at day 63 (Figure 4C). PCR-adjusted efficacy for DHAPQ and ASAQ was similar at most sites, with the exception of Pamol (Nigeria), where ASAQ performed significantly better than DHAPQ (Figure 4A). The PCR-adjusted efficacy of ASAQ and AL were similar at most sites, with the exception of Nanoro (Burkina Faso), where at day 28 ASAQ performed significantly better than AL (Figure 4B).

Parasite clearance was rapid in all treatment groups as the large majority of patients had no detectable infection at day 3, with no major differences in the time of clearance between treatment groups (Table 4). Similarly, about 60% of patients had fever at baseline (Table 2), while at day 3 more than 95% of patients were afebrile, with no major differences among treatment groups in terms of fever clearance time (Table 4).

**Table 4:** Secondary outcomes by pair-wise comparison.

Outcome	ASAQ versus DHAPQ	AL versus ASAQ	AL versus DHAPQ	CD+A versus ASAQ	CD+A versus DHAPQ	CD+A versus AL
Parasite clearance time (days)^a^	1.00 (0.91, 1.10)	0.92 (0.82, 1.03)	0.91 (0.84, 0.99)	1.04 (0.84, 1.28)	1.03 (0.90, 1.19)	1.10 (1.02, 1.20)
Fever clearance time (days)^a^	1.08 (0.99, 1.19)	0.88 (0.79, 0.99)	0.95 (0.87, 1.03)	0.95 (0.77, 1.17)	0.99 (0.87, 1.14)	1.10 (0.81, 1.32)
**Gametocyte carriage: all patients** b						
OR (for excess of patients with zero gametocytes)	0.88 (0.70, 1.11)	1.39 (1.05, 1.82)	1.26 (1.01, 1.57)	1.11 (0.63, 1.96)	0.81 (0.57, 1.14)	0.50 (0.31, 0.81)
Ratio of gametocyte carriage days	1.05 (0.97, 1.14)	0.59 (0.52, 0.66)	0.68 (0.62, 0.76)	0.82 (0.66, 1.02)	0.89 (0.76, 1.04)	0.95 (0.75, 1.20)
**Gametocyte carriage: patients without gametocytes at enrollment** b						
OR (for excess of patients with zero gametocytes)	0.75 (0.56, 1.00)	1.29 (0.93, 1.79)	1.02 (0.77, 1.36)	0.94 (0.38, 2.29)	0.74 (0.44, 1.27)	0.38 (0.18, 0.82)
Ratio of gametocyte carriage days	1.16 (1.00, 1.33)	0.53 (0.44, 0.63)	0.63 (0.53, 0.74)	0.44 (0.31, 0.87)	1.08 (0.76, 1.54)	1.19 (0.72, 1.96)
Change Hb (g/dl) day 0–day 3^c^	−0.04 (−0.13, 0.05)	0.05 (−0.06, 0.16)	0.02 (−0.06, 0.10)	−0.24 (−0.47, −0.01)	−0.38 (−0.51, −0.25)	−0.26 (−0.43, −0.08)
Change Hb (g/dl) day 0–day 7^c^	−0.05 (−0.16, 0.05)	0.07 (−0.06, 0.21)	0.11 (0.01, 0.20)	−0.41 (−0.67, −0.15)	−0.48 (−0.64, −0.32)	−0.49 (−0.70, −0.27)
Change Hb (g/dl) day 0–day 14^c^	0.12 (−0.04, 0.27)	−0.05 (−0.23, 0.13)	0.11 (−0.02, 0.24)	−0.34 (−0.85, 0.17)	−0.27 (−0.52, −0.03)	−0.22 (−0.51, 0.06)
Change Hb (g/dl) day 0–day 28^c^	0.05 (−0.10, 0.20)	−0.17 (−0.35, 0.01)	−0.03 (−0.16, 0.10)	−0.26 (−0.61, 0.09)	−0.31 (−0.53, −0.09)	−0.13 (−0.45, 0.18)

Values are hazard ratio (95% CI) or OR (95% CI), as indicated.

a Hazard ratio of treatment (first listed) versus control.

b Analysis based on zero-inflated Poisson models reported as the OR of excess gametocytes during follow-up, and the ratio of gametocyte carriage days (for those with gametocytes).

c Mean change (difference) between day 0 and day of follow-up.

**Figure 5:**
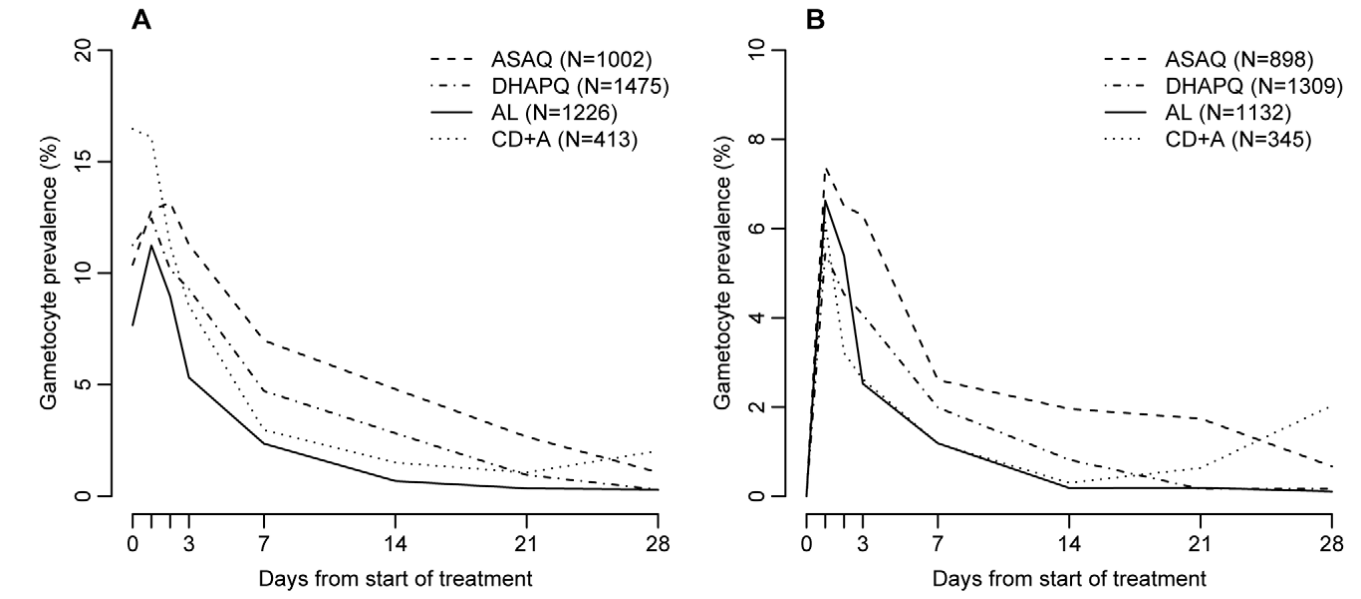
Gametocyte prevalence by treatment and day of follow-up. (A) All patients regardless of gametocytemia at enrollment; (B) patients with gametocytes at enrollment excluded.

The evolution of gametocyte prevalence by treatment group is shown in Figure 5. Gametocyte prevalence during follow-up was significantly lower in patients treated with AL than in those treated with DHAPQ (OR: 0.79, 95% CI: 0.64–0.99), ASAQ (OR: 0.72, 95% CI: 0.55–0.95), or CD+A (OR: 0.50, 95% CI: 0.31–0.82) (Table 4). However, when excluding patients with gametocytes at enrollment, AL had significantly lower gametocyte prevalence only for the pair-wise comparison with CD+A (Table 4). The time (in days) of gametocyte carriage was significantly shorter in the AL group than in the ASAQ and DHAPQ groups, both when including all patients and excluding those with gametocytes at enrollment (Table 4).

**Figure 6:**
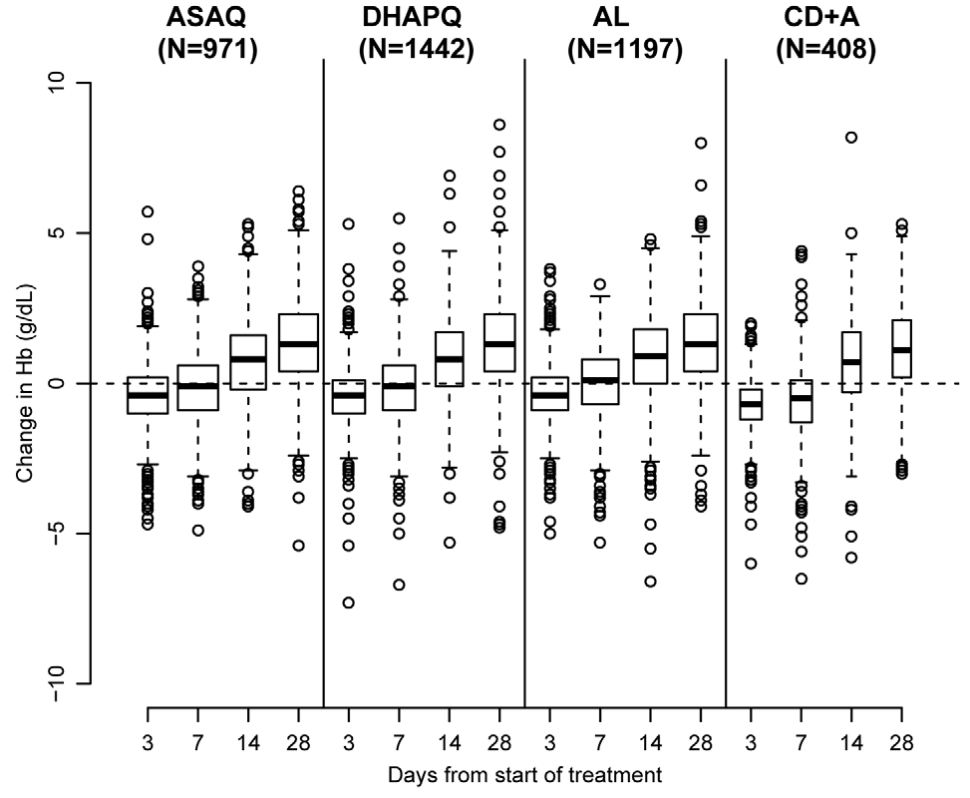
Hematological recovery by treatment and day of follow-up. Hematological recovery determined by Hb changes compared to day 0. The boxplots contain the following information: median (bold line), first and third quartile (box), whiskers extending to 1.5× interquartile range, and all more extreme values.

Hb increased in all treatment groups and attained the day 0 levels at day 7, except in patients treated with CD+A (Figure 6). In the three pair-wise analyses without CD+A, i.e., AL versus DHAPQ, AL versus ASAQ, and ASAQ versus DHAPQ, no important differences in Hb change were found. Conversely, in all pair-wise analyses involving CD+A, Hb recovery was significantly slower in the CD+A than in the other treatment group, with significant differences at days 3 and 7 (Table 4).

### Safety Results

A total of 4,108 patients were included in this analysis. Up to day 28, 37 patients experienced SAEs, their occurrence being relatively more frequent in patients treated with ASAQ or CD+A than among those treated with DHAPQ or AL (Table 5). Most SAEs judged by the site investigator to be related to the study treatment occurred during the first week of follow-up, including four anemia cases, two of them in the CD+A group. There were five deaths, none of them considered related to treatment, a severe malaria case in the ASAQ group, one death due to diarrheal disease in the DHAPQ group, a sudden death in the CD+A group, and two deaths in the AL group (severe malaria and unknown cause) (Table 5). In addition, between days 28 and 63, a severe malaria death occurred in the AL group.

**Table 5:** Summary of adverse events up to day 28 (for patients having received at least one dose).

Safety Population	ASAQ (*n* = 1,003)	DHAPQ (*n* = 1,468)	AL (*n* = 1,225)	CD+A (*n* = 412)
**At least one AE**	669 (66.7)	963 (65.6)	758 (61.9)	314 (76.2)
**Most common AEs** a				
Anemia	143 (14.3)	141 (9.6)	38 (3.1)	71 (17.2)
Diarrhea	112 (11.2)	166 (11.3)	142 (11.6)	40 (9.7)
Vomiting	106 (10.6)	123 (8.4)	102 (8.3)	52 (12.6)
Pyrexia	215 (21.4)	371 (25.2)	339 (27.7)	173 (42.0)
Hb decreased	40 (4.0)	103 (7.0)	83 (6.8)	70 (17.0)
Anorexia	94 (9.4)	130 (8.9)	121 (9.9)	55 (13.3)
Cough	314 (31.3)	470 (32.0)	387 (31.6)	155 (37.6)
**At least one related AE**	233 (23.2)	291 (19.8)	200 (16.3)	101 (24.5)
**Most common related AEs** a				
Anemia	98 (9.8)	96 (6.5)	31 (2.5)	61 (14.8)
Vomiting	59 (5.9)	49 (3.3)	36 (2.9)	15 (3.6)
**SAE**	15 (1.5)	10 (0.7)	6 (0.5)	6 (1.5)
**Related SAE** b	4 (0.4)	4 (0.3)	2 (0.2)	2 (0.5)
**At least one AE that caused discontinuation**	2 (0.2)	2 (0.1)	1 (0.1)	1 (0.2)
**At least one SAE that caused death**	1 (0.1)	1 (0.1)	2 (0.2)	1 (0.2)

Values are *n* (percent).

a AEs and related AEs recorded in, respectively, at least 10% and 5% of patients in any treatment group.

b A related SAE is a SAE that the investigator classified as possibly, probably, or definitely related to the study drug.

During the first 28 days of follow-up, over 60% of patients experienced at least one AE (Table 5). Vomiting occurred in about 10% of patients (ASAQ: 10.6%; DHAPQ: 8.4%; AL: 8.3%; CD+A: 12.6%), while decreased Hb identified as an AE occurred more frequently in the CD+A group (Table 5). Similarly, anemia was diagnosed more frequently in the CD+A group, while the AL group had the lowest occurrence (Table 5).

The median levels of alanine aminotransferase and creatinine before treatment, as well as the proportion of patients with values above the normal range (both clinically and non-clinically significant, the latter not shown), were similar between the four study arms, and this did not change during the follow-ups at day 7 and 28 (Table 6).

**Table 6:** Biochemistry tests for liver and renal function before and after treatment.

Test	Treatment Group			
	ASAQ	DHAPQ	AL	CD+A
**ALAT with clinically significant values above normal range (** ***n/N*** **)**				
Day 0	1.8 (17/930)	0.7 (10/1403)	1.4 (16/1165)	1.5 (6/406)
Day 7	1.1 (9/808)	0.2 (3/1315)	0.4 (4/1077)	0.0 (0/395)
Day 28	1.0 (7/713)	0.3 (4/1248)	0.1 (1/913)	0.0 (0/290)
**ALAT, median (IQR)**				
Day 0	23.0 (16.0–33.0)	23.3 (17.0–33.0)	23.8 (16.4–33.3)	25.0 (19.1–36.4)
Day 7	22.0 (15.4–31.0)	22.3 (16.6–30.2)	23.0 (16.9–31.1)	23.0 (17.9–33.9)
Day 28	21.0 (15.0–29.0)	22.0 (16.4–30.0)	23.0 (16.2–31.0)	23.0 (18.0–30.7)
**Creatinine with clinically significant values above normal range (** ***n/N*** **)**				
Day 0	0.2 (2/903)	0.1 (2/1372)	0.0 (0/1165)	0.0 (0/406)
Day 7	0.1 (1/782)	0.0 (0/1292)	0.0 (0/1072)	0.0 (0/401)
Day 28	0.0 (0/680)	0.0 (0/1199)	0.2 (2/909)	0.7 (2/293)
**Creatinine, median (IQR)**				
Day 0	38.0 (31.4–44.2)	44.2 (34.0–58.3)	45.1 (35.4–62.0)	40.0 (31.0–60.0)
Day 7	37.1 (31.0–44.2)	44.2 (34.3–60.1)	45.1 (36.0–60.1)	38.0 (26.5–53.0)
Day 28	38.0 (30.3–44.2)	44.2 (34.3–60.1)	49.5 (40.8–62.8)	41.5 (31.0–61.0)

Liver function: alanine aminotransferase (ALAT; IU/l); renal function: creatinine (µmol/l). Sample size indicated in the denominator of proportions of values above the normal range.

IQR, interquartile range.

## Discussion

This large multicenter trial aimed to collect information that would assist national malaria control programs in sub-Saharan African countries in choosing the most appropriate ACTs for their specific setting. The four ACTs most likely to be considered for this purpose at the time the study was conceived and implemented, namely AL, ASAQ, DHAPQ, and CD+A, were compared at 12 sites distributed over seven countries. The development of CD+A was discontinued partway through the study, following the results of a phase III clinical trial that showed a higher risk for severe and clinically concerning Hb decrease [25], while a new ACT, pyronaridine-artesunate, is likely to become an additional ACT to be considered [26].

Each site tested three of the four ACTs, i.e., in ten sites both AL and DHAPQ were tested; in six of these the third arm was ASAQ and in the other four, CD+A; two additional sites tested ASAQ, DHAPQ, and CD+A. Following the discontinuation of the CD+A arm, six sites continued testing only two study treatments. Such study design does not allow the use of the pooled results of the four study treatments, as the efficacy of a given ACT tested in one site cannot be directly compared with that of another ACT tested only in another site. Therefore, it is necessary to analyze the six pair-wise comparisons and to include in each comparison only sites in which the two study treatments were actually tested.

When considering the pooled data across all sites, non-inferiority could be established for the three pair-wise comparisons without CD+A, indicating that ASAQ, AL, and DHAPQ had similar and high PCR-adjusted efficacy, both at day 28 and 63; efficacy was lower for CD+A. Conversely, the unadjusted efficacy estimates were higher for DHAPQ, followed by ASAQ and then AL, though at day 63 the difference between DHAPQ and ASAQ seemed to disappear; also in this case CD+A had a much lower efficacy than the other three treatments. It is important to mention that beyond day 28, the detection of recurrent infections was passive, i.e., only sick children attending the health facility had a blood slide prepared, so that some asymptomatic infections may not have been detected. Nevertheless, the important difference in the PCR-unadjusted efficacy between day 28 and 63 suggests that during this period a substantial proportion of children had a recurrent malaria infection, mostly a new infection, as indicated by the small difference between the PCR-adjusted efficacy at day 28 and 63.

The site-specific estimates mirror the overall results, with some notable and surprising exceptions. DHAPQ had the highest PCR-unadjusted efficacy in several sites known for their intense malaria transmission, e.g., Nanoro (Burkina Faso) and Tororo (Uganda), so that the difference observed can be explained by the long post-treatment prophylactic period related to the slow elimination of piperaquine. This is also the reason why DHAPQ performed better than ASAQ at the only high-transmission site where it was tested, i.e., Nanoro (Burkina Faso). Nevertheless, it is surprising that PCR-unadjusted efficacy for DHAPQ was significantly better than that of AL and ASAQ in Ndola (Zambia), where transmission has decreased dramatically over the past few years, to the extent that the target sample size for this trial could not be attained. Unlike Tororo (Uganda), where the PCR-adjusted efficacies for AL and DHAPQ were similar, in Ndola (Zambia), such efficacy tended to be higher for DHAPQ, and the difference probably did not reach statistical significance because of the small sample size, though AL efficacy was almost 95%. Similarly, at this site the difference between DHAPQ and ASAQ PCR-adjusted efficacy was of borderline significance, though ASAQ efficacy was about 94%. It is unclear what such differences mean, as all treatments, with the exception of CD+A, had a PCR-adjusted efficacy >90% and the trial aimed at showing non-inferiority at a 10% difference threshold. Non-inferiority was demonstrated for the three pair-wise comparisons involving DHAPQ, ASAQ, and AL at most sites, with a few exceptions where the individual sites' 95% CI cross the non-inferiority limit.

In west Africa, ASAQ continues to have excellent efficacy, comparable to that of AL [27],[28], while in east Africa doubts about its use have been expressed [29]. Indeed, in a study carried out in Kampala, Uganda, ASAQ had a lower efficacy than AL, a result confirmed in subsequent years [30]. The superior efficacy of AL compared to ASAQ was explained by the presence of amodiaquine resistance in east Africa that may render this ACT increasingly less efficacious, similar to what has been observed for sulfadoxine-pyrimetamine-artesunate in east Africa [29]. In Tanzania, AL was significantly better than ASAQ, but this was an effectiveness study, i.e., the treatment administration was not supervised, and ASAQ was not co-formulated, two important factors that may have influenced the treatment outcome [31]. In our study, although ASAQ was tested mainly in west Africa, it also had excellent efficacy at sites located in eastern and southern Africa, namely Mbarara (Uganda), Ndola (Zambia), and Manhiça (Mozambique). However, the choice of the ACTs to test at the individual sites was influenced by their known drug resistance profile, i.e., ASAQ was not tested in sites with known high amodiaquine resistance. Therefore, although ASAQ is a possible option for some countries in east Africa, it should be not be deployed where amodiaquine resistance is known to be high. For the PCR-unadjusted efficacy at days 28 and 63, none of the pair-wise comparisons could show non-inferiority. Instead, DHAPQ was the best treatment, followed by ASAQ, AL, and then CD+A, which was consistently inferior to the three other ACTs. Considering that most of the recurrent infections were due to new infections and that the risk of re-infection depends on the activity of the non-artemisinin component, this result is largely expected. Indeed, piperaquine has the longer elimination half-life (about 23–28 d), followed by amodiaquine (3 wk), lumefantrine (3.2 d) [32], chlorproguanil (35 h), and dapsone (27 h) [33]. The length and efficacy of the post-treatment prophylaxis may also be influenced by the transmission intensity. In Tororo (Uganda), a site with very high entomological inoculation rates (>500 infective bites/person/year) [34], the difference in the cumulative risk of recurrent malaria between AL and DHAPQ decreased when the follow-up period was extended to 63 d, suggesting that piperaquine's long elimination half-life could do little against the overwhelming risk of recurrent malaria [35]. Nevertheless, in our study and at the same site, the risk of recurrent infections at day 63, though still extremely high, was lower in the DHAPQ than in the AL arm. When considering the forest plot comparing AL and DHAPQ, the OR tended to be lower at sites with the highest transmission intensity, suggesting that piperaquine's longer post-treatment prophylaxis still had an effect despite the high transmission. It should also be noted that places with an intensity of transmission as high as or higher than that of Tororo are not common, particularly in the current context of decreasing malaria burden in sub-Saharan Africa [1]. Though delaying the occurrence of a second clinical attack, via a long post-treatment prophylactic effect, represents an advantage at the individual level, the increased risk of selecting resistant parasites among the new infections should be considered. Such risk occurs during a specific period, the “window of selection,” whose opening and duration is proportional to the drug terminal elimination half-life [36]. Therefore, according to this model, such window would be shorter for lumefantrine (3–5 wk) than for piperaquine. Though this should not be a deterrent for the large-scale deployment of DHAPQ, setting up a reliable early warning system for the detection of resistance, possibly by both in vivo and in vitro tests, would be essential.

In children treated with AL, gametocyte prevalence during follow-up and gametocyte carriage time were significantly lower than in children treated with either DHAPQ or ASAQ. The difference remained significant for gametocyte carriage time even when excluding patients with gametocytes at enrollment. Higher gametocyte carriage after treatment with DHAPQ, when compared to either AL or mefloquine-artesunate, has already been reported in some [14],[35],[37]–[39] but not all trials [40]. Similarly, gametocyte carriage was higher after treatment with ASAQ than with AL in some [29],[41] but not all studies [27]. The meaning of such differences in terms of transmission potential is unclear. Compared to molecular methods, microscopy detects only a small fraction of gametocyte carriers, both in individuals with asymptomatic infections [42] and in patients treated with an antimalarial [39]. Children with microscopically detectable gametocytes are more likely to be infectious but those with sub-microscopic gametocytes can also transmit, albeit less efficiently [42]. The gametocyte prevalence as determined by molecular methods has been observed to be higher in patients treated with DHAPQ than with AL [39]. However, about 60% of children treated with AL and without microscopically detectable gametocytes were infectious to mosquitoes, with little difference between treatments, though the probability of a mosquito becoming infected was significantly lower for the ACT (AL and sulfadoxine-pyrimetamine-artesunate) than for monotherapy or non-ACT combinations [43]. This indicates the difficulty of determining the transmission potential on the basis of the gametocyte carriage time as determined by microscopy, so that the differences in gametocyte carriage observed in our trial may not necessarily relate to a significantly different transmission potential. Considering that ACTs reduce the production of gametocytes by both decreasing the asexual reservoir and destroying a substantial proportion of immature, developing gametocytes, still sequestered in the microvasculature [44], and that parasite clearance was similar in the four study arms, the difference observed between the three ACTs may relate to their ability to clear almost mature forms not released in the blood stream yet.

Hematological recovery up to day 28 post-treatment was similar for all ACTs tested except for CD+A, for which this was significantly slower, with a more marked Hb decrease up to day 7, confirming previous results [25]. In addition, in the CD+A group, anemia was diagnosed more frequently as AE, providing additional evidence for the higher risk of anemia for this treatment. Aside from anemia risk related to CD+A, all regimens were well tolerated.

In conclusion, this is, to our knowledge, the largest head-to-head comparison of most of the currently available ACTs for falciparum malaria in sub-Saharan Africa. CD+A was suspended partway through the trial, leaving AL, ASAQ, and DHAPQ under investigation. These three ACTs showed excellent efficacy, up to day 63 post-treatment, but the risk of recurrent infections was significantly lower, even in areas of high transmission, for DHAPQ, followed by ASAQ, and then AL. Although the gametocyte carriage rate differed between regimens, with those treated with AL having the lowest carriage rate and those treated with ASAQ having the highest carriage rate, the meaning of these different carriage rates with relation to transmission potential is unclear. The possibility of adding a single dose of primaquine to any of these three ACTs, with the objective of further reducing gametocyte carriage, should be explored [41],[45]. AL and/or ASAQ are already included in the antimalarial drug policies of many sub-Saharan African countries. This study confirms that DHAPQ is a valid third option for the treatment of uncomplicated *P. falciparum* malaria, as its efficacy is excellent and comparable to the other ACTs, while its long post-treatment prophylaxis could be an additional advantage.

## Supporting Information

Text S1 **Study protocol.**
(PDF)

Text S2 **CONSORT checklist.**
(PDF)

Text S3 **Protocol amendment 1.**
(PDF)

Text S4 **Protocol amendment 2.**
(PDF)

Text S5 **Protocol amendment 3.**
(PDF)

## Abbreviations

ACPR: adequate clinical and parasitological response
ACT: artemisinin-based combination therapy
AE: adverse event
AL: artemether-lumefantrine
ASAQ: amodiaquine-artesunate
CD+A: chlorproguanil-dapsone-artesunate
CI: confidence interval
DHAPQ: dihydroartemisinin-piperaquine
Hb: hemoglobin
OR: odds ratio
ITT: intention to treat
SAE: serious adverse event
WHO: World Health Organization

## References

[1] Prudhomme O'Meara W, Nekesa Mangeni J, Steketee R, Greenwood B (2010). Changes in the burden of malaria in sub-Saharan Africa.. Lancet Infect Dis.

[2] World Health Organization (2009). World malaria report 2009.

[3] Sharp BL, Kleinschmidt I, Streat E, Maharaj R, Barnes KI (2007). Seven years of regional malaria control collaboration—Mozambique, South Africa, and Swaziland.. Am J Trop Med Hyg.

[4] Sharp BL, Ridl FC, Govender D, Kuklinski J, Kleinschmidt I (2007). Malaria vector control by indoor residual insecticide spraying on the tropical island of Bioko, Equatorial Guinea.. Malar J.

[5] Nyarango PM, Gebremeskel T, Mebrahtu G, Mufunda J, Abdulmumini U (2006). A steep decline of malaria morbidity and mortality trends in Eritrea between 2000 and 2004: the effect of combination of control methods.. Malar J.

[6] Bhattarai A, Ali SS, Kachur SP, Mårtensson A, Abbas AK (2007). Impact of artemisinin-based combination therapy and insecticide-treated nets on malaria burden in Zanzibar.. PLoS Med.

[7] Fegan GW, Noor AM, Akhwale WS, Cousens S, Snow RW (2007). Effect of expanded insecticide-treated bednet coverage on child survival in rural Kenya: a longitudinal study.. Lancet.

[8] Nosten F, White NJ (2007). Artemisinin-based combination treatment of falciparum malaria.. Am J Trop Med Hyg.

[9] Bosman A, Mendis KN (2007). A major transition in malaria treatment: the adoption and deployment of artemisinin-based combination therapies.. Am J Trop Med Hyg.

[10] Bathurst I, Hentschel C (2006). Medicines for Malaria Venture: sustaining antimalarial drug development.. Trends Parasitol.

[11] D'Alessandro U (2009). Existing antimalarial agents and malaria-treatment strategies.. Expert Opin Pharmacother.

[12] World Health Organization (2010). Guidelines for the treatment of malaria, 2nd edition.

[13] Medicine for Malaria Venture (2007). Orphan drug status for MMV drug DHA-piperaquine.. http://www.mmv.org/newsroom/news/orphan-drug-status-mmv-drug-dha-piperaquine.

[14] Bassat Q, Mulenga M, Tinto H, Piola P, Borrmann S (2009). Dihydroartemisinin-piperaquine and artemether-lumefantrine for treating uncomplicated malaria in African children: a randomised, non-inferiority trial.. PLoS ONE.

[15] Valecha N, Phyo AP, Mayxay M, Newton PN, Krudsood S (2010). An open-label, randomised study of dihydroartemisinin-piperaquine versus artesunate-mefloquine for falciparum malaria in Asia.. PLoS ONE.

[16] World Health Organization (2010). Global report on antimalarial drug efficacy and drug resistance: 2000–2010.

[17] Luzzatto L (2010). The rise and fall of the antimalarial Lapdap: a lesson in pharmacogenetics.. Lancet.

[18] World Health Organization Communicable Diseases Cluster (2000). Severe falciparum malaria.. Trans R Soc Trop Med Hyg.

[19] World Health Organization (2008). Methods and techniques for clinical trials on antimalarial drug efficacy: genotyping to identify parasite populations.

[20] Plowe CV, Djimde A, Bouare M, Doumbo O, Wellems TE (1995). Pyrimethamine and proguanil resistance-conferring mutations in Plasmodium falciparum dihydrofolate reductase: polymerase chain reaction methods for surveillance in Africa.. Am J Trop Med Hyg.

[21] World Health Organization (2003). Assessment and monitoring of antimalarial drug efficacy for the treatment of uncomplicated falciparum malaria.

[22] Stepniewska K, Taylor WR, Mayxay M, Price R, Smithuis F (2004). In vivo assessment of drug efficacy against Plasmodium falciparum malaria: duration of follow-up.. Antimicrob Agents Chemother.

[23] Engels EA, Schmid CH, Terrin N, Olkin I, Lau J (2000). Heterogeneity and statistical significance in meta-analysis: an empirical study of 125 meta-analyses.. Stat Med.

[24] Newcombe RG (1998). Interval estimation for the difference between independent proportions: comparison of eleven methods.. Stat Med.

[25] Premji Z, Umeh RE, Owusu-Agyei S, Esamai F, Ezedinachi EU (2009). Chlorproguanil-dapsone-artesunate versus artemether-lumefantrine: A randomized, double-blind phase III trial in African children and adolescents with uncomplicated *Plasmodium falciparum* malaria.. PLoS ONE.

[26] Tshefu AK, Gaye O, Kayentao K, Thompson R, Bhatt KM (2010). Efficacy and safety of a fixed-dose oral combination of pyronaridine-artesunate compared with artemether-lumefantrine in children and adults with uncomplicated *Plasmodium falciparum* malaria: a randomised non-inferiority trial.. Lancet.

[27] Ndiaye JL, Randrianarivelojosia M, Sagara I, Brasseur P, Ndiaye I (2009). Randomized, multicentre assessment of the efficacy and safety of ASAQ—a fixed-dose artesunate-amodiaquine combination therapy in the treatment of uncomplicated Plasmodium falciparum malaria.. Malar J.

[28] Faye B, Offianan AT, Ndiaye JL, Tine RC, Toure W (2010). Efficacy and tolerability of artesunate-amodiaquine (Camoquin plus) versus artemether-lumefantrine (Coartem) against uncomplicated Plasmodium falciparum malaria: multisite trial in Senegal and Ivory Coast.. Trop Med Intern Health.

[29] Dorsey G, Staedke S, Clark TD, Ndjama-Meya D, Nzarubara B (2007). Combination therapy for uncomplicated falciparum malaria in Ugandan children: a randomized trial.. JAMA.

[30] Clark TD, Njama-Meya D, Nzarubara B, Maiteki-Sebuguzi C, Greenhouse B (2010). Incidence of malaria and efficacy of combination antimalarial therapies over 4 years in an urban cohort of Ugandan children.. PLoS ONE.

[31] Mutabingwa TK, Anthony D, Heller A, Hallett R, Ahmed J (2005). Amodiaquine alone, amodiaquine+sulfadoxine-pyrimethamine, amodiaquine+artesunate, and artemether-lumefantrine for outpatient treatment of malaria in Tanzanian children: a four-arm randomised effectiveness trial.. Lancet.

[32] Douglas NM, Anstey NM, Angus BJ, Nosten F, Price RN (2006). Artemisinin combination therapy for vivax malaria.. Lancet Infect Dis.

[33] Simpson JA, Hughes D, Manyando C, Bojang K, Aarons L (2006). Population pharmacokinetic and pharmacodynamic modelling of the antimalarial chemotherapy chlorproguanil/dapsone.. Br J Clin Pharmacol.

[34] Okello PE, Van Bortel W, Byaruhanga AM, Correwyn A, Roelants P (2006). Variation in malaria transmission intensity in seven sites throughout Uganda.. Am J Trop Med Hyg.

[35] Arinaitwe E, Sandison TG, Wanzira H, Kakuru A, Homsy J (2009). Artemether-lumefantrine versus dihydroartemisinin-piperaquine for falciparum malaria: a longitudinal, randomized trial in young Ugandan children.. Clin Infect Dis.

[36] Stepniewska K, White NJ (2008). Pharmacokinetic determinants of the window of selection for antimalarial drug resistance.. Antimicrob Agents Chemother.

[37] Smithuis F, Kyaw MK, Phe O, Aye KZ, Htet L (2006). Efficacy and effectiveness of dihydroartemisinin-piperaquine versus artesunate-mefloquine in falciparum malaria: an open-label randomised comparison.. Lancet.

[38] Grande T, Bernasconi A, Erhart A, Gamboa D, Casapia M (2007). A randomised controlled trial to assess the efficacy of dihydroartemisinin-piperaquine for the treatment of uncomplicated falciparum malaria in Peru.. PLoS ONE.

[39] Mens PF, Sawa P, van Amsterdam SM, Versteeg I, Omar SA (2008). A randomized trial to monitor the efficacy and effectiveness by QT-NASBA of artemether-lumefantrine versus dihydroartemisinin-piperaquine for treatment and transmission control of uncomplicated Plasmodium falciparum malaria in western Kenya.. Malar J.

[40] Ratcliff A, Siswantoro H, Kenangalem E, Maristela R, Wuwung RM (2007). Two fixed-dose artemisinin combinations for drug-resistant falciparum and vivax malaria in Papua, Indonesia: an open-label randomised comparison.. Lancet.

[41] Smithuis F, Kyaw KM, Phe O, Win T, Aung PP (2010). Effectiveness of five artemisinin combination regimens with or without primaquine in uncomplicated falciparum malaria: an open-label randomised trial.. Lancet Infect Dis.

[42] Ouédraogo AL, Bousema T, Schneider P, de Vlas SJ, Ilboudo-Sanogo E (2009). Substantial contribution of submicroscopical Plasmodium falciparum gametocyte carriage to the infectious reservoir in an area of seasonal transmission.. PLoS ONE.

[43] Bousema TJ, Schneider P, Gouagna LC, Drakeley CJ, Tostmann A (2006). Moderate effect of artemisinin-based combination therapy on transmission of Plasmodium falciparum.. J Infect Dis.

[44] Okell LC, Drakeley CJ, Ghani AC, Bousema T, Sutherland CJ (2008). Reduction of transmission from malaria patients by artemisinin combination therapies: a pooled analysis of six randomized trials.. Malar J.

[45] Shekalaghe SA, ter Braak R, Daou M, Kavishe R, van den Bijllaardt W (2010). In Tanzania, hemolysis after a single dose of primaquine coadministered with an artemisinin is not restricted to glucose-6-phosphate dehydrogenase-deficient (g6pd a−) individuals.. Antimicrob Agents Chemother.

[46] Tinto H, Sanou B, Erhart A, D'Alessandro U, Ouédraogo JB (2006). In vivo sensitivity of Plasmodium falciparum to chloroquine and sulfadoxine-pyrimethamine in the Bobo Dioulasso region (1998–2001): risk factors associated with treatment failures to the two drugs.. Bull Soc Pathol Exot.

[47] Borrmann S, Binder RK, Adegnika AA, Missinou MA, Issifou S (2002). Reassessment of the resistance of Plasmodium falciparum to chloroquine in Gabon: implications for the validity of tests in vitro vs. in vivo.. Trans R Soc Trop Med Hyg.

[48] Lell B, Lehman LG, Schmidt-Ott JR, Sturchler D, Handschin J (1998). Malaria chemotherapy trial at a minimal effective dose of mefloquine/sulfadoxine/pyrimethamine compared with equivalent doses of sulfadoxine/pyrimethamine or mefloquine alone.. Am J Trop Med Hyg.

[49] Ezedinachi E (1996). In vivo efficacy of chloroquine, halofantrine, pyrimethamine-sulfadoxine and ginghaosu (artesunate) in the treatment of malaria in Calabar, Nigeria.. Cent Afr J Med.

[50] Mulenga M, Malunga P, Bennett S, Thuma P, Shulman C (2006). Folic acid treatment of Zambian children with moderate to severe malaria anemia.. Am J Trop Med Hyg.

[51] Tinto H (2005). Plasmosium falciparum drug resistance: molecular markers, in vivo and in vitro tests [PhD dissertation].

[52] Talisuna AO, Langi P, Bakyaita N, Egwang T, Mutabingwa TK (2002). Intensity of malaria transmission, antimalarial-drug use and resistance in Uganda: what is the relationship between these three factors?. Trans R Soc Trop Med Hyg.

[53] Talisuna AO, Okello PE, Erhart A, Coosemans M, D'Alessandro U (2007). Intensity of malaria transmission and the spread of Plasmodium falciparum resistant malaria: a review of epidemiologic field evidence.. Am J Trop Med Hyg.

[54] Alonso PL, Sacarlal J, Aponte JJ, Leach A, Macete E (2004). Efficacy of the RTS,S/AS02A vaccine against Plasmodium falciparum infection and disease in young African children: randomised controlled trial.. Lancet.

[55] Abacassamo F, Enosse S, Aponte JJ, Gómez-Olivé FX, Quintó L (2004). Efficacy of chloroquine, amodiaquine, sulphadoxine-pyrimethamine and combination therapy with artesunate in Mozambican children with non-complicated malaria.. Trop Med Int Health.

[56] Legros D, Johnson K, Houpikian P, Makanga M, Kabakyenga JK (2002). Clinical efficacy of chloroquine and sulfadoxine-pyrimethamine in children under five from southwestern Uganda with uncomplicated falciparum malaria.. Trans R Soc Trop Med Hyg.

