# Supplementary material for: A Head-to-Head Comparison of Four Artemisinin-Based Combinations for Treating Uncomplicated Malaria in African Children: A Randomized Trial
Source: PLoS Med. 2011 Nov 8;8(11):e1001119. doi: 10.1371/journal.pmed.1001119 (PMC3210754; doi:10.1371/journal.pmed.1001119)

Evaluation of 4 artemisinin-based combinations for treating uncomplicated malaria in African children

Co-ordinating Investigator

Professor Umberto D'Alessandro
Prince Leopold Institute of Tropical Medicine
Nationalestraat 155
B-2000 Antwerp-Belgium

**Protocol No.:**	
**Date:**	October 5th thth , 2006
**Version:**	7th version
**Title:**	Evaluation of 4 artemisinin-based combinations for treating uncomplicated malaria in African children
**Countries :**	7 countries: Burkina Faso, Nigeria, Gabon, Uganda, Rwanda, Zambia and Mozambique
**Sites :**	10 sites: Nanoro (BF) Calabra (NG), Lambaréné (GN), Mbarara, Jinja and Tororo (UG), Rukara and Mashesha (RW), Ndola (ZB), Manhiça (MZ)
**Study drugs**	Dihydroartemisinin+Piperaquine, Amodiaquine+Artesunate, Artemether+Lumefantrine, Chlorproguanil-Dapsone+Artesunate
**Sponsor:**	Prince Leopold Institute of Tropical Medicine, Antwerp, Belgium
**Co-ordinating Investigator:** **Address:**	Professor Umberto D'Alessandro Prince Leopold Institute of Tropical Medicine, Nationalestraat 155, B-2000, Antwerp, Belgium
**Telephone:**	0032 3 247 6354
**Fax:**	0032 3 247 63 59
**Email:**	udalessandro@itg.be

STUDY ACKNOWLEDGMENT/CONFIDENTIALITY

By signing this protocol, the Investigator(s) acknowledges and agrees:

The protocol contains all necessary information for conducting study. The Investigator will conduct this study as detailed herein and will make every reasonable effort to complete the study within the time designated.

The protocol and all relevant information on the drug relating to pre-clinical and prior clinical experience, which was provided by the Sponsor, will be made available to all physicians, nurses and other personnel who participate in conducting this study. The Investigator will discuss this material with them to assure that they are fully informed regarding the drug(s) and the conduct of the study.

This document contains information that is privileged or confidential. As such, it may not be disclosed unless specific permission is granted in writing by the Prince Leopold Institute of Tropical Medicine, Antwerp, Belgium, or such disclosure is required by federal or other laws or regulations. In any event, persons to whom the information is disclosed must be informed that the information is privileged or confidential and may not be further disclosed by them. These restrictions on disclosure will apply equally to all future information supplied which is indicated as privileged or confidential.

The Prince Leopold Institute of Tropical Medicine, Antwerp, Belgium will have access to any source documents from which Case Report Form information may have been generated. The Case Report Forms and any other data pertinent to this study are the property the Prince Leopold Institute of Tropical Medicine, Antwerp, Belgium, who may utilise the data in various ways, such as publication of the results of this multicentre study.

The conduct and results of this study will be kept confidential until all sites have completed the study, unless an interim publication or presentation is agreed upon. The results of this multicentre study will be published. Furthermore, each Investigator may independently publish the data at that particular site. The procedures to be followed for the publication of data are defined in the “Consortium Agreement EDCTP Clinical Trial” at section 10: Publications, Press Releases and Reports to the *EDCTP*.

Approval	Signature	Date
Prof./Dr. ………..……..	________________________________<br>Investigator	____/____/2006___
Prof. Umberto D'Alessandro	________________________________<br>Co-ordinating Investigator	____/____/2006___
Prof. Bruno Gryseels	________________________________<br>Prince Leopold Institute of Tropical Medicine<br>Director	____/____/2006___

Signing this document I declare to have read the paragraph relevant to study acknowledgement and confidentiality and authorise the Prince Leopold Institute of Tropical Medicine, Antwerp, Belgium to record my data on a computerised archive containing all the data pertinent to the study.

Index

Synopsis 6

1. Rationale and background 11
2. Trial objectives and purpose 14
3. Trial design 14
4. Concomitant therapies 25
5. Patient withdrawal criteria 26
6. Protocol violations 26
7. Safety variables 26
8. Case report form 30
9. Monitoring and quality assurance 30
10. Data management 31
11. Investigator responsibility 31
12. Administrative procedures 32
13. Study committee 32
14. Ethical issues 33
15. References 34
16. Appendices 36

SYNOPSIS

Title

Evaluation of 4 artemisinin-based combinations for treating uncomplicated malaria in African children

Methodology

A multicentre, phase IV, randomized, open label, multi-arm study. Each patient will be actively followed for 28 days and then passively until 6 months after treatment. If, during the passive follow up, the patient experiences a second uncomplicated malaria episode, s/he will be treated with the same drug and actively followed up for additional 28 days.

Follow up chart first and second active follow up.

Day	0	1	2	3	4	5	6	7	14	21	28	Any other day[1]
History (symptoms)	X							X	X	X	X	X
Informed consent	X[2]											
Examination (clinical)	X	X	X	X				X	X	X	X	X
Temperature	X	X	X	X				X	X	X	X	X
Blood film	X	X	X	X				X	X	X	X	X
Filter paper PCR	X	X							X	X	X	X
Adverse drug reactions	X	X	X	X	X[5]	X[5]	X[5]	X	X	X	X	X
Concomitant medications	X	X	X	X				X	X	X	X	X
Haematology	X	X[3]	X[3]	X				X	X		X	X
Biochemistry	X							X	X[4]		X	
Treatment	X	X	X									

[1] Spontaneous attendance to health facility; [2] Only at the beginning of the first follow up. [3] In sites where CDA is tested, Hb will be determined also at day 1 and 2 after treatment and for the three arms of the study; [4] If abnormal at day 7. [5] If patient attends spontaneously the health facility.

Study Duration

Calendar of activity.

**YEAR**	**1**				**2**				**3**			
**QUARTERS**	**1**	**2**	**3**	**4**	**1**	**2**	**3**	**4**	**1**	**2**	**3**	**4**
**Preparatory phase**	■											
Meeting of research groups (coordination)		■										
Production of final detailed protocol		■										
CRF and data base design		■										
Ethical Committee approval		■										
Information/agreement with study populations		■										
Training in GCP		■										
Training data entry and data management		■										
**Implementation phase**												
Coordination meetings (teleconferences every 3 months)		■				■				■		
Monitoring initiation visit		■										
Recruitment of patients			■	■	■	■						
(Second) Follow-up					■	■	■	■				
Monitoring visits (3 times per site)					■	■	■	■				
Closure monitoring visit									■	■		
Interim analysis				■	■	■	■	■				
Genotyping blood samples								■	■	■		
Data Analysis											■	■
Publications											■	■

Countries

7 African countries: Burkina Faso, Nigeria, Gabon, Uganda, Mozambique, Zambia, Rwanda.

Study Center(s)

10 sites

Objectives

The main objective is to compare the safety and efficacy of 4 artemisinin-based combinations (ACT) [amodiaquine-artesunate (AQ+AS), dihydroartemisinin-piperaquine (DHAPQ), artemether-lumefantrine (AL) and chlorproguanil/dapsone-artesunate (CDA)] for single and repeat treatments of uncomplicated malaria in children. Safety will be determined by registering adverse events and grading, laboratory, and vital signs evaluations. Their incidence will be compared between the different study arms.

Specific objectives are the following:

1. To evaluate the efficacy of the 4 ACTs for the treatment of children with uncomplicated *P. falciparum* malaria (first active follow-up);
2. To determine after the first active follow-up the incidence rate of a second clinical episode of uncomplicated *P. falciparum* malaria;
3. To evaluate the efficacy of treating the second clinical episode of uncomplicated *P. falciparum* malaria with the same ACT used for the first one (second active follow-up);
4. To evaluate during the active and passive follow up the safety of the 4 ACTs for the treatment of children with uncomplicated *P. falciparum* malaria;

5. To establish the impact of using CDA on the selection of *P. falciparum* genotypes linked to SP resistance.

Number of Subjects

170 patients/arm/site, 510 patients/site, 5100 in total. The total number of patients treated by each study drug will be more than 1000. For the direct comparisons between treatment arms, the highest number of patients per arm will be 1360 (DHAPQ vs AL), and the lowest 340 for the direct comparison between AQ+AS and CDA. (see table below).

Study treatment to be tested by country and institutions.

Country	Numb. sites	Affiliation	Study treatments		
Burkina Faso	1	Centre Muraz/IRSS	AQ+AS	DHAPQ	AL
Nigeria	1	TDRI	AQ+AS	DHAPQ	AL
Zambia	1	TDRC	AQ+AS	DHAPQ	AL
Gabon	1	HAS/Tubingen	AQ+AS	DHAPQ	AL
Uganda	2	EANMAT	DHAPQ	CDA	AL
Uganda	1	EPICENTRE	AQ+AS	CDA	DHAPQ
Rwanda	2	EANMAT	DHAPQ	CDA	AL
Mozambique	1	Manhiça	AQ+AS	CDA	DHAPQ

CQR: Chloroquine resistance; SPR: sulfadoxine-pyrimethamine resistance; AQ+AS: amodiaquine-artesunate; DHAPQ: dihydroartemisinin-piperaquine; AL: artemether-lumefantrine ; CDA : chlorproguanil-dapsone-artesunate;

Diagnosis and Main Inclusion Criteria

Children 6-59 months old (this criterion applies only for the first active follow up) (in sites testing CDA the age group for the 3 study arms will be 12-59 months) with suspected uncomplicated clinical malaria attending the health facility where the study is carried out will be recruited into the study and randomised to one of the study treatments if they fulfil all the following inclusion criteria: 1. Weight > 5 kg; 2. Monoinfection with *P. falciparum* and a parasitaemia of 2,000–200,000 asexual parasites per µl; 3. Fever (axillary temperature ≥37.5 °C) or history of fever in the preceding 24 hours; 4. Haemoglobin value ≥ 7.0 g/dl; 5. Signed informed consent by the parents or guardians; 6. Parents' or guardians' willingness and ability to comply with the study protocol for the duration of the trial.

They will be excluded if they have at least one of the following criteria:

1. Participation in any other investigational drug study (antimalarial or others) during the previous 30 days
2. Known hypersensitivity to the study drugs
3. Severe malaria*.
4. Danger signs: not able to drink or breast-feed, vomiting (> twice in 24hours), recent history of convulsions (>1 in 24h), unconscious state, unable to sit or stand;
5. Presence of intercurrent illness or any condition (cardiac, renal, hepatic diseases) which would place the subject at undue risk or interfere with the results of the study, including known G6PD deficiency.
6. Severe malnutrition (defined as weight for height <70% of the median NCHS/WHO reference)
7. Ongoing prophylaxis with drugs having antimalarial activity such as cotrimoxazole for the prevention of *Pneumocisti carini* pneumonia in children born to HIV+ women.

*WHO 2000: Severe falciparum malaria. *Trans. R. Soc. Trop. Med. Hyg.* **94**, 1-90.

Children infected with other malaria species and those with mixed malaria infections will be excluded from the study but adequate treatment will be provided. Cases of severe and complicated malaria will be referred to hospital for treatment with intravenous quinine and other supportive therapy.

Study endpoints

Primary end points

1. PCR unadjusted treatment failure (TF28U): all treatment failures detected during the active follow up, regardless of genotyping.
2. PCR adjusted treatment failure up to day 28 (TF28A): all early failures before day 14 plus the recurrent parasitaemias detected at day 14 or later and classified by genotyping as recrudescence.

Secondary endpoints (first and second active follow-up)

- PCR unadjusted treatment failure up to day 63 (TF63U): TF28U plus all cases of recurrent parasitaemia (symptomatic or asymptomatic) detected between day 29 and day 63 by passive follow up, regardless of genotyping
- PCR adjusted treatment failure for the whole period of passive surveillance (TFAPS): TF28A plus all episodes of recurrent parasitaemia identified as recrudescence by genotyping.
- Fever clearance time.
- Asexual parasite clearance time.
- Gametocytaemia (prevalence and density) at day 7, 14, 21 and 28 after treatment (for both active follow-ups);
- Hb changes day 3, 7, 14 and 28 (first and second follow up);
- Clinical malaria after first active follow-up;
- Clinical malaria after second active follow-up;
- TF second clinical episode (D28 and D63);
- Changes in the frequency of mutations in the dihydrofolate reductase (DHFR) gene at day 0 first follow-up and day re-appearance of parasitaemia (for patients treated with CDA).
- Safety profiles including significant changes in relevant laboratory values.

Statistical analysis

For the proposed trials each pairwise comparison of treatments will be considered, stratified by site. Heterogeneity due to patient-level and site-level characteristics will be investigated. Finally, all data will be entered into a regression model and a network meta-analytic approach adopted[1]. For each pairwise comparison, both an intention-to-treat, a per-protocol and a Compliance Adjusted Causal Effect (CACE) approach will be adopted. Within each site patients in each treatment group will be described separately with respect to baseline characteristics. The clinical importance of any imbalance will be noted though statistical tests of significance will not be undertaken. For treatment failure, the relative treatment effect will be summarised in terms of the odds ratio and confidence interval, provided no significant unexplained heterogeneity is evident on this scale. If appropriate, logistic regression will be

[1] Lumley T. Network meta-analysis for indirect treatment comparisons. *Statistics in Medicine* 2002; **21**: 2313-2324.

used to examine the effect of prognostic factors on this outcome. Tests of interaction between baseline characteristics and treatment will be kept to a minimum. Adverse events will be grouped according to a pre-specified side-effect coding system and tabulated. The number (and percentage) of patients experiencing each adverse event will be compared across treatment groups. The number (and percentage) of occurrences of each adverse event will also be compared across treatment groups. No formal statistical testing will be undertaken.
Every effort will be made to minimise the amount of missing data in the trial. However it is realistic to assume a priori that missingness may be informative. Whenever possible, information on the reason for missing data will be obtained. Sensitivity analyses, including imputation, will be considered to assess the robustness of the conclusions to the missing data.

Interim analyses will be undertaken by the trial statistician and presented to the DSMB every six months during recruitment, i.e. approximately after every 500 patients per group have been entered. A detailed analysis plan will be drawn up prior to the first interim analysis.

Institutions involved

- Institute of Tropical Medicine, Antwerp, Belgium: Coordination
- Liverpool School of Tropical Medicine and Centre for Medical Statistics and Health Evaluation, University of Liverpool, UK
- East African Network for Monitoring Antimalarial Treatment (EANMAT).
- Centre Muraz, Bobo Dioulasso, Burkina Faso.
- Department of Paediatrics, University of Calabar, Cross River State, Nigeria.
- Tropical Diseases Research Centre, Ndola, Zambia
- Institute of Tropical Medicine, Department of Parasitology, University of Tuebingen, Germany and Medical Research Unit, Albert Schweitzer Hospital, Lambaréné, Gabon.
- Uganda Malaria Surveillance Project (UMSP), Kampala, Uganda.
- Epicentre, Paris, France and Mbarara University of Science and Technology, Faculty of Medicine, Mbarara, Uganda
- Programme National de Lutte contre le Paludisme, Kigali, Rwanda.
- Fundacio Clinic per a la Recerca Biomèdica/Centre for International Health, University of Barcelona, Spain and Manhiça Health Research Center, Mozambique.

1. RATIONALE AND BACKGROUND

Resistance to commonly used antimalarial drugs represents the major drawback and obstacle for controlling malaria in endemic countries. Currently, the geographic distribution of *P. falciparum* resistance to chloroquine (CQ) almost corresponds to that of the parasite itself and it is severe in many countries.[1] Several countries have changed their first line treatment from CQ to other regimens, usually sulfadoxine-pyrimethamine (SP), but resistance to the latter has rapidly reached critical levels in some areas of East and Central Africa and a public health disaster might be imminent.[1] Unfortunately, only few available alternative drugs are ready for deployment. It is generally agreed that endemic countries should opt for combination treatment, i.e. the association of 2 or more antimalarial drugs with different metabolic targets. Artemisinin derivatives (AS) and consequently artemisinin-containing combinations (ACT) have several advantages as they produce a rapid clinical and parasitological cure, reduce gametocyte carriage rate and are generally well tolerated. In Africa, studies on ACT (artesunate with either CQ, amodiaquine (AQ) or SP) have been carried out in a few thousands children with uncomplicated malaria. Good safety and efficacy were reported when the partner drug was also efficacious.[2] However, at least 2 of the 3 drugs mentioned above (CQ and SP) can no longer be considered as useful partner drugs for combination with AS because of their confirmed low efficacy.[3] Several African countries have already opted for ACT (for example: Burundi for AQ+AS, Zanzibar for AQ+AS and artemether + lumefantrine (AL), Zambia for AL; Kenya, Rwanda, Uganda and Tanzania might or are about to change to AL), but additional information for their optimal deployment is still required. Besides a non-co-formulated AQ+AS, the only other possible alternative is AL, which is moderately expensive and has a complex dosage. The need to provide African Ministries of Health with reliable information on ACT safety, efficacy and effectiveness (NB, the latter is not addressed in this proposal) is obvious. We therefore propose to test 4 combinations, namely amodiaquine + artesunate (AQ+AS), dihydroartemisinin + piperaquine (DHAPQ), artemether + lumefantrine (AL) and chlorproguanil-dapsone + artesunate (CDA), the first 3 being co-formulated. The studies will be carried out in 7 African countries covering a variety of epidemiological situations. All partners involved are experienced in the conduct of clinical trials and most of them have been working in this field for several years. Moreover, the close relationship of institutions such as the EANMAT to the countries' Ministries of Health is a guarantee for the rapid uptake of the project's results for the review and modification of their national antimalarial drug policy.

1.1 Drugs to be tested

Chlorproguanil–dapsone + artesunate (CDA)

Chlorproguanil–dapsone (CD) is a fixed-dose antifolate combination that has been developed jointly by GlaxoSmithKline and WHO/TDR (Tropical Disease Research). CD received approval from the UK Regulatory Agency in July 2003 for the treatment of uncomplicated falciparum malaria in non-pregnant adults and children and has recently become available in several countries in Africa. Unlike sulfadoxine-pyrimethamine (SP), CD is rapidly eliminated from the body (approximate half-lives of chlorproguanil and dapsone are 24h and 12h), resulting in low selection pressure for drug resistance.[10] Data from the Phase III clinical programme conducted in children in sub-Saharan Africa demonstrated that CD achieved significantly higher cure rates compared to SP (96% vs 89%; $p<0.001$) and was well

tolerated.[4, 9, 11-13] Importantly, SP-resistant infections acquired in Africa have been responsive to CD treatment.[4] In contrast to pyrimethamine, chlorproguanil retains therapeutic activity against parasites with the common *dhfr* mutation conferring resistance against pyrimethamine: ser-108-asn, asn-51-ile and cys-59-arg.[14]

CD is well tolerated in young children and most of the serious adverse events recorded are more likely to be disease-related than drug-related.[9] One percent of patients taking CD and 1% of those taking SP reported serious adverse events, most commonly red blood cell disorders. Dapsone is known to cause methaemoglobinaemia and haemolysis[15] (especially in G6PD deficient people).[16,17] Clinically significant increases in methaemoglobin were not seen in SP-treated patients, but were observed in 4.2% of those given CD (all mild cases and 1 moderate case, all of limited duration). None of the children showed dyspnoea, the main symptom of high methaemoglobinaemia, and no action was required by attending physicians[9]. Severe hemolysis is of concern because it is a feature of falciparum malaria, making it difficult to identify cases of drug-induced haemolysis. G6PD-deficient children in the CD group were more likely than similar patients in the SP group to have a > 2g/dL haemoglobin drop when compared to those without G6PD deficiency, but by day 14 Hb values in both groups had been restored to baseline. Larger haemoglobin falls after treatment with CD than after SP have been reported previously.[11] Other genetic polymorphisms, such as those in candidate genes responsible for metabolism of CD, are also potential determinants of the risk of adverse events.

Although Lapdap exerts a significantly lower selective pressure than SP (Nzila et al, 2000), and it is currently highly effective, activity will be greatly reduced by the selection of one further DHFR mutation[14]. This mutation, at codon 164 was selected by SP use in both South America and South-East Asia, and has been found at low frequency in Tanzania[14]. The use of CD as monotherapy may therefore increase selective pressure for the 'quadruple DHFR mutant'. Associating CD with artesunate can considerably increase its antimalarial activity[19] and at the same time might delay the selection of the quadruple DHFR mutants. A trial investigating the safety and efficacy of non co-formulated CD and artesunate in children 6-59 months old with uncomplicated malaria is ongoing in Rwanda. Preliminary results show a relatively good efficacy and no major safety problem.

Artemether –lumefantrine (AL)

This is a fixed-dose combination of artemether (a semi-synthetic artemisinin derivative) and lumefantrine (a slowly eliminated drug also referred to as benflumetol). The registered indications and branding for AL cover treatment of uncomplicated malaria caused by mono or mixed *Plasmodium* infections. The combination is expected to confer mutual protection against resistance and prevent recrudescence after artemether therapy. The components of this combination were originally studied and developed in China by the Academy of Military Medical Sciences (AMMS), Beijing and Kunming Pharmaceutical Factory (KPF), Kunming. The fixed combination has been registered in China since 1992 and has undergone further development when Novartis signed a collaborative agreement in 1994 with AMMS, KPF and CITITEC, the technology arm of the China International Trust and Investment Corporation (CITIC). Studies for the international registration started in 1995. AL was registered in Switzerland in 1999 and has since received marketing authorisation in several endemic and non-endemic countries. Recently, an agreement was reached between Novartis and WHO for AL to be made available to the public sector of developing countries at a preferential price. Few clinical trials, mostly with the four-dose regimen, have been conducted both with the

original Chinese combination product and the subsequent product used for international registration. A Cochrane review [20] has, in 2003, identified eight randomized, controlled trials comparing AL with standard treatment for uncomplicated falciparum malaria (2117 participants). From the meta-analysis, it was concluded that the four-dose AL regimen was superior to CQ and equivalent to SP in areas of CQ resistance but inferior to mefloquine and mefloquine-artesunate in areas of multidrug resistance. The six-dose regimen was also equivalent to mefloquine-artesunate but was better tolerated [21, 22]. A collaborative project between Novartis and WHO has led to a more user-friendly packaging of the six-dose treatment, which is now being field tested. A paediatric formulation is also being developed and trials have been conducted for regulatory submission to extend the label to cover children who weigh less than 10 kg [23].

Artesunate-amodiaquine (AQAS)

Several clinical trials involving AQAS have been completed in Africa. Two trial in Rwanda comparing non-coformulated AQAS with either AQ+SP and with DHAPQ showed that the former is a well tolerated combination with an efficacy at day 28 after treatment above 90% [8, 24,]. A blister pack of AQAS has been developed and several African countries have chosen AQAS as first line treatment. AQAS has now been developed as fixed-dose combination and is undergoing registration after the relevant pre-clinical and clinical studies.

Dihydroartemisinin-piperaquine (DHAPQ)

DHAPQ was developed in China and is registered in China and Cambodia. Non GMP DHAPQ has been evaluated extensively in clinical trials in Thailand, Vietnam, Cambodia and China[25], efficacy has been high and tolerability uniformly good in all trials in multidrug-resistant areas, where piperaquine-resistance was common after its extensive use for mass prophylaxis. In Africa, DHAPQ has been evaluated in Rwanda in children 6-59 months old with uncomplicated malaria. The treatment was highly efficacious (95% adequate clinical and parasitological response at day 28 after treatment) and well tolerated [8]. Initially, the co-formulation included primaquine and trimethoprim (CV8), which is still part of national policy in Vietnam. Piperaquine is an orally active bisquinoline discovered by Rhône-Poulenc in the early 1960s and developed for clinical use in China in 1973. Piperaquine is approximately equivalent to chloroquine against sensitive parasites, but is significantly more effective than chloroquine against resistant *P. falciparum.* Piperaquine replaced chloroquine as the recommended treatment for falciparum malaria in China in 1978. Overall, 194 140 kg of piperaquine phosphate, equivalent to 140 000 000 adult doses, were used for mass prophylaxis and treatment. Surveillance at the time found no adverse events other than rare cases of a rash. However, reports about emerging parasite resistance to piperaquine from southern China, an area of intensive use of piperaquine, are of concern. Dihydroartemisinin is the active metabolite of artesunate and artemether. It has equivalent clinical efficacy to the more widely used artesunate. A development programme has been agreed between Holleykin Pharmaceuticals and Guangzhou University (China), The University of Oxford, MMV, and Sigma-Tau Industrie Farmaceutiche Riunite SpA to support the international registration of the drug. A multi-centre phase III clinical trial comparing DHAPQ with AL in Africa and with mefloquine+artesunate in Asia is ongoing.

2. TRIAL OBJECTIVES AND PURPOSE

The main objective is to compare the safety and efficacy of 4 artemisinin-based combination therapies (ACT), i.e. amodiaquine-artesunate (AQ+AS), artemether-lumefantrine (AL), dihydroartemisinin-piperaquine (DHAPQ), chlorproguanil-dapsone-artesunate (CDA), for single and repeat treatments of uncomplicated malaria in African children. Safety will be determined by registering adverse events and grading, laboratory, and vital signs evaluations. Their incidence will be compared between the different study arms.

Specific objectives are the following:

1. To evaluate the efficacy of the 4 ACTs for the treatment of children with uncomplicated *P. falciparum* malaria (first active follow-up);
2. To determine after the first active follow-up the incidence rate of a second clinical episode of uncomplicated *P. falciparum* malaria;
3. To evaluate the efficacy of treating the second clinical episode of uncomplicated *P. falciparum* malaria with the same ACT used for the first one (second active follow-up);
4. To evaluate during the active and passive follow up the safety of the 4 ACTs for the treatment of children with uncomplicated *P. falciparum* malaria;
5. To establish the impact of using CDA on the selection of *P. falciparum* genotypes linked to SP resistance.

3. TRIAL DESIGN

3.1 *Study Design*

This is a multi- centre, randomized, open label, multi-armed study involving 10 investigational centres that will enrol 5100 patients, 510 per site, 170 per arm. The 4 combinations will be tested and compared in different countries, with different malaria transmission and resistance characteristics (Table 1). A three-arm trial maximises the amount of information collected and, at the same time, keeps the individual studies manageable.

The total number of patients treated by each study treatment will be between 1700 (DHAPQ) and 1020 (AQ+AS and CDA). For the comparisons between treatment arms, the highest number of patients per arm will be 1360 (DHAPQ vs AL), ad the lowest 340 for the comparison between AQ+AS and CDA. This is slightly different from what had been planned in the proposal submitted to the EDCTP. The Rwandan collaborators have requested not to test AQ+AS on the ground that this combination will not be used as first or second line treatment. Indeed, resistance to AQ in Rwanda is already substantial, particularly in the site of Rukara. This is why the arm AQ+AS has been replaced by DHAPQ.

The main justification for not performing a double-blind study is the requirement to use the double-dummy technique requiring placebos for 3 arms drugs per site, resulting into a treatment schedule which would be very laborious for the patient. In order to ensure concealment of treatment allocation and avoid other biases, the following requirements will be assured:

1 The randomization list will be generated prior the beginning of the study. Treatment allocation will be concealed until the completion of the screening and the final recruitment of the patient (see section 3.10);
2 The interpretation of the PCR reading will be blinded/masked with regard to the treatment allocation of the patients (see section 3.9);
3 An independent Data Monitoring Board will review all efficacy and safety data (see section 13.2).

Table 1. Study treatment to be tested by country.

Country	Sites	Transmission (EIR)	CQR %	SPR%	Study treatments		
Burkina Faso	Nanoro	Seasonal high (50-60)	66	27	AQ+AS	DHAPQ	AL
Nigeria	Calabar	Perennial high	45	30	AQ+AS	DHAPQ	AL
Zambia	Ndola	Seasonal mesoendemic	High	>20 in adults	AQ+AS	DHAPQ	AL
Gabon	Lambarené	Perennial high (50)	100	21	AQ+AS	DHAPQ	AL
Uganda	Mbarara	Mesoendemic	81	61	AQ+AS	CDA	DHAPQ
Uganda	Jinja	Seasonal low (6)	28	49 (CQ+SP)	DHAPQ	CDA	AL
Uganda	Tororo	Seasonal high (>70)	45	9-15	DHAPQ	CDA	AL
Rwanda	Rukara	Seasonal high	40	35 (CQ+SP)	DHAPQ	CDA	AL
Rwanda	Mashesha	Seasonal high	50	12	DHAPQ	CDA	AL
Mozambique	Manhiça	Perennial mesoendemic (15)	78	22	AQ+AS	CDA	DHAPQ

EIR: Entomological Inoculation rate; CQR: Chloroquine resistance; SPR: sulfadoxine-pyrimethamine resistance; AQ+AS: amodiaquine-artesunate; DHAPQ: dihydroartemisinin-piperaquine; AL: artemether-lumefantrine ; CDA : chlorproguanil-dapsone-artesunate;

3.2 *Primary Endpoint*

There will be several primary end points:

1. PCR unadjusted treatment failure up to day 28 (TF28U): all treatment failures detected during the first active follow up, regardless of genotyping.
2. PCR adjusted treatment failure up to day 28 (TF28A): all early failures before day 14 plus the recurrent parasitaemias detected at day 14 or later and classified by genotyping as recrudescence.

The TF is defined according to the WHO criteria (WHO 2003) as the sum of early* and late** treatment failures.

*** Early Treatment Failure (ETF) (one of the following)**

(i) Development of danger signs or severe malaria (see Appendix V) on Day 0, Day 1, Day 2 or Day 3, in the presence of parasitaemia,

(ii) Parasite density on Day 2 > Day 0 count, irrespective of axillary temperature,

(iii) Presence of parasitaemia on Day 3 with fever (axillary temperature ≥ 37.5°C),

(iv) Parasitaemia on Day 3 ≥ 25 % of count on Day 0.

**** Late treatment failure (LTF)**

LTF is divided in late clinical and late parasitological failure.

Late Clinical Failure (LCF):

(i) Development of danger signs or severe malaria after Day 3 in the presence of parasitaemia, (See Appendix V for the criteria of severe malaria/danger signs).

(ii) Presence of parasitaemia and fever on any day from Day 4 to Day 28, without having previously meet the criteria of ETF.

Late Parasitological Failure (LPF):

Reappearance of parasitaemia after day 3 in the absence of fever (axillary temperature <37.5°C) without having previously meet the criteria of ETF or LCF.

The adequate clinical and parasitological response (ACPR) is 1-TF (28 or 63, unadjusted or adjusted). It is defined as absence of parasitaemia at the end of the follow up period (day 28-63), irrespective of axillary temperature without previously meeting any of the criteria of early and late treatment failure. In the adjusted estimates, patients with late asexual parasite reappearance (with or without fever) will be considered ACPR if the PCR analysis shows a new infection rather than a recrudescence.

3.3 *Secondary Endpoints*

The secondary endpoints will be:

- PCR unadjusted treatment failure up to day 63 (TF63U): TF28U plus all recurrent parasitaemias (symptomatic or asymptomatic) detected between day 29 and day 63 by passive follow up, regardless of genotyping.

- PCR adjusted treatment failure for the whole period of passive surveillance (TFAPS): TF28A plus all episodes of recurrent parasitaemia identified as recrudescence by genotyping.

- Fever clearance time (FCT): Fever clearance time will be defined as the time (in days) from the time of randomization to the first two consecutive measurements on 2 different days of axillary temperature below 37.5°C.

- Asexual parasite clearance time (PCT): Asexual parasite clearance time will be defined as the time (in days) from time of randomization to 2 consecutive negative blood slides (collected at different days). The time to the event will be taken as the time to the first negative slide.

- Gametocytaemia (prevalence and density) at day 7, 14, 21 and 28 after treatment (for both active follow-ups);

- Hb changes day 3, 7, 14 and 28 (first and second follow up);
- Clinical malaria after first active follow-up;
- Clinical malaria after second active follow-up;
- TF second clinical episode (D28 and D63);
- DHFR mutations at day 0 first follow-up and day re-appearance of parasitaemia (for patients treated with CDA).
- Safety profiles: Subjects will be monitored for 28 days (first and second follow up) for possible development of adverse events. All adverse events will be recorded on the specific form in the CRF. Vital signs, blood chemistry and haematology will be monitored and changes in relevant laboratory parameters will be assessed.

3.4 Interpretation PCR results

Genotyping of the recurrent infection will be done by characterizing MSP1, MSP2 and GLURP, single-copy genes in the *Plasmodium falciparum* genome. PCR-amplification of DNA from a single parasite clone results in a single amplification product. For the three genes, each PCR-amplification product of a different size is considered to originate from a different clone of *Plasmodium falciparum* and reflects a different genotype. For the samples collected from the same patient at day 0 and day of recurrent parasitaemia, the length polymorphism of MSP1, MSP2 and GLURP will be determined, i.e. the number of bands in each PCR reaction and their respective size. Results will be interpreted as follows:

Recrudescence: For each marker (MSP1, MSP2 and GLURP), at least one identical length polymorphism is found in the sample collected at day 0 and day of recurrent parasitaemia.

New infection: For at least one marker, length polymorphism is different between the sample collected at day 0 and that at day of recurrent parasitaemia.

Indeterminate: Samples that failed to produce a result due to an inability to amplify DNA at day 0 and/or day of recurrent parasitaemia.

3.5 ***Sample size***

Sample size is estimated according to the expected efficacy at day 28 after treatment as in Africa there is no information beyond this point. Efficacy of each study treatments is likely to be at least 90%. A sample of 155 children per arm will be able to show, in each site, at the 5% significance level with 90% power, that the difference in efficacy between treatments is not more than 10%. Allowing for a loss to follow–up around 10%, the final sample size per arm will be 170 children. Moreover, when combined, the studies will be able to detect a significant difference in efficacy between treatments of 4-6% and major adverse events occurring at the frequency of at least 1-2%.

3.6 *Statistical analysis*

Since the trial has been powered for equivalence at each site, interim analyses will be undertaken for each of the sites separately. Interim analyses will be undertaken by the trial statistician and presented to the DSMB every 6 months during recruitment while safety monitoring will be continuous. A detailed analysis plan will be drawn up prior to the first interim analysis.

For each analysis, both an intention-to-treat, a per-protocol and a CACE approach will be adopted. Patients in each treatment group in each site will be described separately with respect to baseline characteristics. The clinical importance of any imbalance will be noted though statistical tests of significance will not be undertaken.

For the analysis at each site, and for the primary outcomes relating to treatment failure, the relative treatment effect will be summarised in terms of the risk difference and associated confidence interval for each pairwise combination of treatments.

For the combination of data across sites, a meta-analytic approach will be employed. The six pairwise comparisons of treatments will be undertaken, stratified by site. Heterogeneity due to patient-level and site-level characteristics will be investigated. Finally, all data will be entered into a regression model and a network meta-analytic approach adopted[2].

If appropriate, logistic regression will be used to examine the effect of prognostic factors on the primary outcome. Tests of interaction between baseline characteristics and treatment will be kept to a minimum.

Adverse events will be grouped according to a pre-specified side-effect coding system and tabulated. The number (and percentage) of patients experiencing each adverse event will be compared across treatment groups. The number (and percentage) of occurrences of each adverse event will also be compared across treatment groups.

Every effort will be made to minimise the amount of missing data in the trial. However it is realistic to assume a priori that missingness may be informative. Whenever possible, information on the reason for missing data will be obtained. Sensitivity analyses, including imputation, will be undertaken to assess the robustness of the conclusions to the missing data.

3.7 *Duration of patient follow up*

Each patient will be followed up actively after recruitment for 28 days (first follow up) and then passively for 6 months afterwards. If, within the 6-month period the patient will re-attend the health facility because of clinical malaria, he/she will be actively followed up for 28 days (second follow up) if inclusion and exclusion criteria are fulfilled, otherwise s/he will be treated with the first line drug or any other appropriate antimalarial. From the third episode of clinical malaria onwards, patients will be treated with the first line drug or any other appropriate antimalarial but will not be actively followed up.

3.8 *Follow-up Chart*

All children 6-59 months attending the outpatient clinic with fever or history of fever in the preceding 24 hours will have a thick and thin blood smear done. In sites where CDA is one of

the study drugs, the age range will be 12-59 months old. The parents or guardians of those with a *P. falciparum* infection of the required density will be informed by the health facility staff about the malaria clinical trial and will be requested to freely consent to participate in the trial. Unwillingness or inability to participate will result in the prescription and administration of the local standard treatment regime for uncomplicated *P. falciparum* malaria. The parents or guardians who accept their child to participate in the study will receive detailed explanations about the trial from the study staff. Specifically, they will be informed that three antimalarial drugs are being tested and the option given to the child will be decided randomly (by chance). They will be asked to sign (or thumb-print if illiterate) the informed consent form (see Appendix IV).

For children meeting the inclusion/exclusion criteria, the following steps will be taken:

1. A Case Report Form consisting of demographic and physical/clinical information will be completed;

2. Body weight and axillary temperature will be measured (the latter by using a digital electronic thermometer) and the results will be recorded on the Case Report Form. If the initial temperature value is less than 36°C the measurement will be repeated.

3. A thick blood smear will be obtained for measuring parasite density and a thin blood film for malaria species determination (the sample collected for screening can be used).

4. A blood sample will be collected on Whatman filter paper number 3MM for subsequent genotyping of the infective parasite strains. An additional blood sample to be later analysed for the determination of the G6PD status will be collected from all children in the sites where CDA is tested.

5. Blood sample (2 ml) for haematology/biochemistry will be taken.

At enrolment, children will be assigned a sequential study number. During the first 3 study days (days 0-2) patients will be admitted for observation and study drug administration. Parents/guardians will then be encouraged to return to the clinic for follow up assessments on days 3, 7, 14, 21, 28 and on any unscheduled day if the child is not well.

All medication will be administered under observation by an authorised member of the study team (physician, clinician, or study nurse).

After the child has reached a study outcome, before or at the end (day 28) of the first active follow up, the parents or guardians will be encouraged to attend the health facility when the child is sick. If the child is not diagnosed with clinical malaria, s/he will be treated accordingly and parents or guardians will be asked to come back again whenever the child is sick. If the child has clinical malaria and meets the inclusion/exclusion criteria, s/he will be treated with the same drug received at the beginning of the first follow up and actively followed for 28 days according to the procedure outlined above. Re-treatment with the same study drug will be done at an interval of at least 29 days from the first treatment, i.e. after the first active follow up has finished. At the end of this second follow up, when one of the study

outcomes is reached, the child will be passively followed up until completion of the 6-month period started at the end of the first active follow up. Any malaria episode will be recorded and the patient treated with the standard antimalarial treatment. No active follow up will be carried out.

Patients will be assessed as summarized in the following flow-chart.

Figure 1. Follow up chart first and second active follow up.

Day	0	1	2	3	4	5	6	7	14	21	28	Any other day[1]
History	X							X	X	X	X	X
Informed consent	X[2]											
Examination (clinical)	X	X	X	X				X	X	X	X	X
Temperature	X	X	X	X				X	X	X	X	X
Blood film	X	X	X	X				X	X	X	X	X
Filter paper PCR	X	X							X	X	X	X
Filter paper G6PD**	X											
Adverse drug reactions	X	X	X	X	X[5]	X[5]	X[5]	X	X	X	X	X
Concomitant medications	X	X	X	X				X	X	X	X	X
Haematology	X	X[3]	X[3]	X				X	X		X	X
Biochemistry*	X							X	X[4]		X	
Treatment	X	X	X									

[1] Spontaneous attendance to health facility; [2] Only at the beginning of the first follow up. [3]In sites where CDA is tested, Hb will be determined also at day 1 and 2 after treatment and for the three arms of the study; [4] If abnormal at day 7. [5] If the patient attends spontaneously the health facility.

*Not all sites; **Only in sites where CDA is tested.

3.9 *Selection of the Patients (for the first and second active follow up)*

Inclusion criteria

In order to be eligible, patients should satisfy the following inclusion criteria:

1. Males and Females aged between 6 months and 59 months inclusive. In the sites where CDA is tested all recruited children will be aged between 12 months and 59 months inclusive. This criterion applies only for the recruitment in the first follow up. For the second follow up, children having been included in the first follow up are eligible, regardless of their age.

2. Body weight of 5 Kg and above.

3. Microscopically confirmed, monoinfection of *Plasmodium falciparum* (parasitaemia ≥ 2,000/μL to 200,000/μL).

4. Fever (axillary temperature at ≥ 37.5°C) or history of fever in the previous 24 hours.

5. Haemoglobin value ≥ 7.0 g/dl;

6. Signed (or thumb-printed whenever parents/guardians are illiterate) informed consent

by the parents or guardians. Note the informed consent will be asked only at recruitment and will cover the whole period of the study, including second active follow up and passive case detection.

7. Parents' or guardians' willingness and ability to comply with the study protocol for the duration of the trial.

Exclusion criteria

Patients with any of the following criteria will not be admitted to the study:

1. Participation in any other investigational drug study (antimalarial or others) during the previous 30 days.

2. Known hypersensitivity to the study drugs.

3. Severe malaria.

4. Danger signs: not able to drink or breast-feed, vomiting (> twice in 24hours), recent history of convulsions (>1 in 24h), unconscious state, unable to sit or stand.

5. Presence of intercurrent illness or any condition (cardiac, renal, hepatic diseases) which in the judgement of the investigator would place the subject at undue risk or interfere with the results of the study, including known G6PD deficiency.

6. Severe malnutrition (defined as weight for height <70% of the median NCHS/WHO reference).

7. Ongoing prophylaxis with drugs having antimalarial activity such as cotrimoxazole for the prevention of *Pneumocisti carini* pneumonia in children born to HIV+ women.

3.10 ***Study Procedures***

The critical steps for the study period are described in Appendix VII.

3.10.1. ***Active follow up (first and second)***

Day 0: screening visit/ administration of the study medication

1 Demographic Data and Medical History
Demographic data and a general history of past and present illnesses will be recorded.

2 Informed consent
A signed informed consent (or thumb-printed whenever the parents/guardians are illiterate) from the parent/guardian shall be obtained before any tests or evaluations related to the study eligibility are carried out. However, a thick blood film before the informed consent can be done as this can be considered a normal procedure for the management of patients suspected having clinical malaria.

3 Physical and Clinical Examination

A general physical examination will be performed (see Appendix I).

A clinical examination will be performed (see Appendix I): symptoms and axillary temperature (electronic thermometer).

4 Vital Signs and Weight

Vital signs (heart rate) and weight will also be measured.

5 Blood Slide

A thick and thin blood smear will be obtained from the subject to verify the presence of *P. falciparum* and to calculate the parasite density. Thick and thin blood films will be prepared, dried and stained with Giemsa stain according to standard operating procedures.

Parasite density will be calculated by counting the number of asexual parasites per 200 leukocytes in the thick blood film, based on an assumed WBC of 8,000 /µl by light microscopy at 1000xmagnification. One hundred high-powered fields (HPF) will be examined (independent of presence or absence of asexual parasite stages). The parasite density per microlitre will be calculated using the following formula:

$$\text{Parasite density} / \mu l = \frac{\text{Number of parasites counted x 8,000}}{\text{Number of leukocytes counted}}$$

6 PCR

A blood sample will be collected on filter paper (Whatmann 3MM) at day 0 before treatment and on day 1 and, from day 14 onwards, every time a blood slide is done. Samples from patients classified as late treatment failure will be subsequently used for parasite genotyping. An additional blood sample at day 0 will be collected for later determination of the G6PD status. The collection will be carried out only in sites where CDA is tested.

7 Laboratory Tests

Blood haemoglobin, red blood cell count, total white blood cell count, differential count.

Total bilirubin, ALAT and creatinine will be measured.

8 Administration of the Study Drugs

9 Adverse Events Report

All adverse events will be recorded. See section 7.1.4 for the details on the information collected.

10 Concomitant Medications

Any medications taken by the study subject will be recorded in the CRF.

Day 1 and 2: *Open Label Treatment Period*

1 Physical and Clinical Examination

A general physical examination and a clinical examination will be performed: symptoms, axillary temperature (electronic thermometer).

2 Vital Signs
Vital signs (heart rate) will be measured.

3 Blood Slide
A thick and thin blood smear will be obtained to verify the presence and to determine the density of asexual and sexual stages of *P. falciparum*.

4 PCR
A blood sample will be collected at day 1 on filter paper (Whatmann 3MM) for later genotyping.

5 Concomitant Pharmacological Treatments
Concomitant medications being taken by the patients will be recorded. For a list of allowed and disallowed medications, see section 4.

6 Adverse Events Report
All adverse events will be recorded. See section 7.1.4 for the details on the information collected

7 Laboratory Tests
Blood haemoglobin in sites where CDA is one of the study arms.

8 Administration of the Study Drugs

Day 3: *Open Label Treatment Period*

1 Physical and Clinical Examination
A general physical examination and a clinical examination will be performed: symptoms, axillary temperature (electronic thermometer).

2 Vital Signs
Vital signs (heart rate) will be measured.

3 Blood Slide
A thick and thin blood smear will be obtained to determine the presence and the density of asexual and sexual stages of *P. falciparum.*

4 Concomitant Pharmacological Treatments
Concomitant medications being taken by the patients will be recorded. For a list of allowed and disallowed medications, see section 4.

5 Adverse Events Report
All adverse events will be recorded. See section 7.1.4 for the details on the information collected

6 Laboratory Tests
Blood haemoglobin, red blood cell count, total white blood cell count, differential count.

Day 7, 14 and 21: *Open Label Treatment Period*

As Day 3. In addition, the following information will be collected:

1 Medical History
Symptoms and other relevant episodes between visits will be recorded.

2 Laboratory Tests
Total bilirubin, ALAT and creatinine will be measured. At day 14 only if abnormal at day 7. No haematology or biochemistry done at day 21.

3 PCR
A blood sample will be collected on filter paper (Whatmann 3MM) starting from day 14 onwards for later genotyping.

Day 28: *Open Label Treatment Period*

As Day 7.

1 PCR
A blood sample will be collected on filter paper (Whatmann 3MM) for later genotyping.

Unscheduled visits throughout follow up

During this visits, the same procedures as day 21 will be applied. Hb will be measured if the patient is classified as treatment failure.

The PCR readings will be centralised and masked to the treatment allocation of study subjects, i.e. by personnel different from the treating physician/investigator. In addition, a centralized and independent double-check of a significant percentage of blood slides and filter paper blood samples (PCR) will be carried-out. The percentages of slides and films to be reviewed and the corresponding statistical justifications will be specified in the Statistical Analysis Plan.

3.10.2. *Passive follow up (6 months after the first active follow up)*

Patients identified as treatment failures (clinical and parasitological) within the first follow up of 28 days will be treated with a full course of quinine or any other appropriate oral treatment (unrelated to the study drugs). Parents/guardians of children who have either been treated or classified as ACPR, i.e. with no malaria infection at day 28 of the first follow up will be asked to attend the health facility whenever the child is sick for the next 6 months. Each child will be visited monthly at home to keep the contact with the health facility. However, no data or blood slide will be collected unless the child is sick. Any attendance of children belonging to the cohort recruited for the first follow up will be recorded. The child will be clinically assessed and the medical history will be taken. Body temperature and a blood sample for

parasitaemia, Hb and PCR will be systematically collected. A diagnosis will be made and the child treated accordingly. Whenever an episode of uncomplicated malaria is diagnosed, the inclusion and exclusion criteria (section 3.8) will be checked and if these are fulfilled, the child will be re-treated with the same drug received for the first episode and will be followed up for the next 28 days (second active follow-up) with the same methodology as the first active follow-up. After the second active follow up, even if the child attend with an episode of clinical malaria fulfilling the inclusion/exclusion criteria, s/he will be treated with the local first line drug and no active follow up will be carried out after treatment. Children with a malaria infection but not fulfilling the inclusion and exclusion criteria will be treated with the local first line drug and their parents will be informed that they are still part of the cohort under passive surveillance. Children will reach the end of the study at the end of the 6-month passive follow up when they will be visited at home and the parents/guardians will be informed that the study is finished (Appendix VIII).

3.11 Randomisation and treatment allocation

A randomisation list of blocks of varying size and stratified according to the number of recruitment points in each site will be provided by the Centre for Medical Statistics and Health Evaluation/ University of Liverpool. Sealed envelopes labelled with the patients unique code and containing the treatment allocated to the patient will also be provided by Liverpool according to the above mentioned list. This will guarantee concealment as the envelope will be opened only after recruitment.

3.12 Treatment administration

The study treatment will be administered by health workers under the direct supervision of a licensed physician. The correct number of tablets will be determined using the weight dosing chart. If necessary the study drugs (either whole or as fractions) will be crushed, dissolved in water and squirted into the mouth using a syringe without needle. Administration of all treatments, including AL will be directly observed. After drug administration, patients will be kept for one hour in the clinic. A dose will be repeated in full if vomiting occurs within 30 minutes of administration and halved if vomiting is between 30 minutes and 1 hour post dosing. This event will be documented in the case record form (CRF). If vomiting persists, the patient will be withdrawn from the study and treatment changed (e.g. quinine).

4. CONCOMITANT THERAPIES

4.1 *Disallowed Concomitant Drug Therapies during the active follow ups*

Any antimalarial, or antibiotic with antimalarial activity (erythromycin or other macrolides, co-trimoxazole or other sulfonamides, any tetracycline including doxycycline, and quinolones, clindamycin). Intake of these drugs leads to withdrawal of the patient from the ongoing active follow up. However, the patient will be kept in the passive follow up and when presenting with another clinical malaria episode fulfilling the inclusion/exclusion criteria, another active follow up can be started. Drugs known to cause haemolysis (e.g. sulpha drugs) should not be administered in children in the CDA arm. There are no disallowed concomitant therapies during the passive follow up.

4.2 *Allowed Concomitant Drug Therapies*

During the trial patients can be prescribed drugs e.g. paracetamol, and antibiotics with no known antimalarial activity (penicillins, cephalosporins). The dose, route, time and duration of any concomitant medical treatment will recorded in the CRF.

4.3 *Special Conditions*

Parents or guardians will be discouraged from obtaining drugs from any other source such as private pharmacies, markets or clinics. Parents/guardians will be encouraged to bring their children to the study clinic if their child is unwell or if they are worried about their child's health.

4.4 *Rescue Treatments*

Active follow up of patients with treatment failure, including parasitological failure, will be stopped and they will be treated with rescue treatment. This consists in quinine 10 mg/kg orally three times a day for 7 days or an antimalarial treatment according to the country's national guidelines. Any patient, who is diagnosed with severe malaria or danger signs during follow-up, will be referred for treatment with parenteral quinine and supportive measures at the local facility or hospital. After the rescue treatment the patient will still be considered in the passive follow up until completion of the 6-month period.

5. PATIENT WITHDRAWAL CRITERIA

Patients will be excluded from further assessment if there is withdrawal of informed consent. Severe adverse events related to the study drug are also a reason for withdrawal from the study drug but not withdrawal from study follow-up.

6. PROTOCOL VIOLATIONS

A protocol violation occurs when an event happens that does not allow for accurate interpretation of response to treatment. Protocol violations will be defined in the statistical analysis plan. All patients should be followed up until the end of the passive follow up unless fulfilling the study withdrawal criteria.

7. SAFETY VARIABLES

Safety and tolerability of the treatments will be evaluated by recording Adverse Events (AEs) and grading, laboratory, and vital signs evaluations.

7.1 *Adverse Events*

At each visit, the Investigator will ascertain the occurrence of any adverse events since the last visit. Any event must be recorded on the CRF.

7.1.1 <u>Definition of an adverse event</u>

An AE is any untoward medical occurrence in a patient or clinical investigation subject administered a pharmaceutical product and which does not necessarily have a causal relationship with this treatment.

An AE can therefore be any unfavourable and unintended sign (that could include a clinically significant abnormal laboratory finding), symptom or disease temporally associated with the use of a medicinal product, whether or not considered related to the medicinal product.

7.1.2 *Severity, relationship of event to study drug, and outcome*

The severity of a clinical adverse event is to be scored according to the following scale:

1	Mild	Awareness of sign or symptom, but easily tolerated
2	Moderate	Discomfort enough to cause interference with usual activity
3	Severe	Incapacitating with inability to work or perform usual activity
4	Life-threatening	Patients at risk of death at the time of the event

The relationship of an adverse event to study drug is to be assessed according to the following definitions:

1 Definitely unrelated

Should be reserved for those events which occur prior to test drug administration (e.g., washout or single-blind placebo) or for those events which cannot be even remotely related to study participation (e.g. injury caused by a third party).

2 Unlikely

There is no reasonable temporal association between the study drug and the suspected event and the event could have been produced by the subject's clinical state or other modes of therapy administered to the subject.

3 Possible

The suspected adverse event may or may not follow a reasonable temporal sequence from study drug administration but seems to be the type of reaction that cannot be dismissed as unlikely. The event could have been produced or mimicked by the subject's clinical state or by other modes of therapy concomitantly administered to the subject.

4 Probable

The suspected adverse event follows a reasonable temporal sequence from study drug administration, abates upon discontinuation of the drug, and cannot be reasonably explained by the known characteristics of the subject's clinical state.

5 Definitely related

Should be reserved for those events which have no uncertainty in their relationship to test drug administration: this means that a rechallenge was positive.

The outcome of each AE must be assessed according to the following classification:

- completely recovered :	The patient has fully recovered with no observable residual effects
- not yet completely recovered :	Improvement in the patient's condition has occurred, but the patient still has some residual effects
- deterioration :	The patient's overall condition has worsened
- permanent damage :	The AE has resulted in a permanent impairment
- death :	The patient died due to the AE
- ongoing :	The AE has not resolved and remains the same as at onset
- unknown :	The outcome of the AE is not known because the patient did not return for follow-up (lost to follow-up)

7.1.3 *Definition of a serious adverse event*

A serious adverse event (experience) (SAE) or reaction is any untoward medical occurrence that at any dose fulfils at least one of the following criteria:

* results in death;
* is life-threatening;
* requires hospitalization (other than for drug administration) or prolongation of existing hospitalization;
* results in persistent or significant disability/incapacity;
* requires a specific medical or surgical intervention to prevent one of the outcomes listed above;

All serious adverse events, whether or not deemed drug-related, or expected, must be reported immediately or within 24 hours (one working day), using the Serious Adverse Event Notification Form , by telefax or email to :

The chairman of the IRB
Institute of Tropical Medicine
Nationalestraat 155
B-2000 Antwerp, Belgium

Tel: +32 3 247 63 25
Fax: +32 3 247 63 59
Email: DVMelle@itg.be

Fax should state "Urgent Serious Adverse Event" on cover page.

All other AEs not fulfilling the criteria of immediate reporting must be recorded on the Case Report Form. This AE information will be collected on a regular basis during the clinical trial. Considering that the study drugs have already been tested in several hundreds of patients and that the study is not a double-blinded one, a Local Safety Monitor is not needed.

7.1.4 *Reporting of adverse events*

For all adverse events identified, an adverse event report form will be completed.

For each possible adverse event identified and considered as **serious**, a serious adverse event notification form will be completed.

The following information will be recorded for all adverse events:

1) Study randomization number
2) Date of birth
3) Sex
4) Description of event
5) Date of event onset
6) Date event reported
7) Severity of the event
8) Study medication including start/stop dates
9) Relationship of the event to study medication
10) Is the event serious?
11) Initials of the person reporting the event
12) Was the event episodic or intermittent in nature?
13) Outcome of adverse event
14) Action taken
15) Date event resolved.

A severity grading scale, based on toxicity grading scales developed by the WHO and the National Institutes of Health, Division of Microbiology and Infectious Diseases, will be used to grade severity of all symptoms, physical exam findings, and haemoglobin results (see Appendix I). Any new event, or an event present at baseline that is increasing in severity, will be considered as an adverse event.

7.1.5 *Length of follow-up for adverse events*

AEs presenting during the active follow-up period: A patient still experiencing an AE at the end of the active follow up, i.e. at day 28 will be managed as follows:

- If the AE has been detected and reported before the last visit and
  - it is mild (Grade 1), the patient will be managed according to good medical practice and the active follow up will be stopped. The end date for the AE will be recorded as Day 28.
  - Its grade is more than, the patient will be followed until the AE resolves, improves, or stabilizes.
- If the AE is new, the AE will be reported and the patient will be followed until the AE resolves, improves, or stabilizes.

7.2 *Laboratory Evaluations*

Blood samples will be properly labelled with patients' initials, randomisation number, protocol number, the study day and the date the sample is taken. Haematology and Clinical chemistry assessments will be performed locally at sites.

All laboratory results will be reported in Standard International Units or in conventional units.

Haematology and Blood Chemistry Tests

Haemoglobin, red blood cells count, total white blood cell count, differential count, total bilirubin, ALAT, creatinine.

Blood samples collected on filter paper for PCR genotyping will be analysed at the Institute of Tropical Medicine, Antwerp, Belgium. Sample will be collected according to standard operating procedures.

Abnormal Laboratory Test Results

The Investigator will mark in the CRF the laboratory values out of normal ranges (the biochemistry results will be specific to each site) and will indicate those of clinical importance. These will be considered as AEs, and the proper AE reporting procedure should be followed by the Investigator.

8. CASE REPORT FORM (CRF)

Presentation of the CRF

The CRF to be used for the study consists of pages headed with the study code and other relevant information. It is composed of an introductive part for the selection and inclusion of patients in the study and special forms for the different evaluation times; at the end of the CRF are the forms for registration of possible adverse events and for any suspension of the study. Each page includes a header containing information for the identification on the study subject (Study number and Initials; to be completed by the Investigator) and the study code.

How to use the CRF

It is recommended that the CRF be filled out using a ballpoint pen with black ink.
All requested information must be entered on the CRFs. If an item is not available or is not applicable, this fact should be indicated; there should be no blank spaces. A correction should be made by striking through the incorrect entry with a single line and by entering the correct information adjacent to it. The correction must be initialled, dated and explained if necessary by the Investigator or by a qualified individual specifically designated by the Investigator. Each completed Case Report Form must be reviewed, signed and dated by the Investigator.

9. MONITORING AND QUALITY ASSURANCE

The task of the Monitor is to verify the best conduct of the study through frequent contacts by phone and in person with the Principal Investigator and site staff, in accordance with the Standard Operating Procedures and Good Clinical Practice, with the purposes of facilitating the work and attaining the objectives of the study. These visits will enable the Monitor to maintain current, personal knowledge of the study through review of the records, comparison with source documents, observation and discussion of the conduct of the study with the Investigator. Each site will be visited 3 times during the conduct of the trial plus a set up visit before the start of clinical activities and a closure visit after the last patient has completed the follow up. The monitor will carry out 30% source data verification.

The investigator must maintain source documents for each patient in the study, consisting in case and visit notes (hospital or clinic medical records) containing demographic and medical information, laboratory data, and the results of any other tests or assessments. All information on the CRF must be traceable to these source documents in the patient's file. The investigator must keep the original informed consent form signed by the patient. The investigator must give the monitor access to all relevant source documents to confirm their consistency with the CRF entries.

The Investigator by signing this protocol declares that he/she will permit trial-related monitoring, audits, Independent Ethic Committee review, and regulatory inspections, providing direct access to source data/documents.

The Investigator agrees to conduct the present study in full agreement with the principles of the "Declaration of Helsinki" and subsequent relevant amendments (see Appendix III).

10. DATA MANAGEMENT

Clinical data, as requested in the protocol, will be collected and recorded on appropriate paper Case Report Forms (CRF's). Laboratory data, as requested in the protocol will be registered at the laboratory and recorded onto the CRF. All data will then be processed from the CRF's into a Clinical Data Management System. During the conduct of the study data will be verified and reviewed to produce and maintain high quality data. All unresolved issues will be queried and resolved before locking the database. Data transfer and handling is done with appropriate security measures and with regard to rights, safety and well-being of trial subjects. A report on data management process will be produced. The report will include

- A full field listing and description of the file structure of the electronic data
- Reference ranges and units for laboratory data
- A list a brief description of all programs run on the data
- Level of errors found at each stage of checking the data
- General comments on data quality and significant problems encountered with the data
- A detailed list of any unresolved data queries
- A statement of any queries/errors which have not been corrected on the database
- A statement of the storage location of the electronic database

The statistical team (Liverpool) will review the database prior to finalisation. They will report on any problem encountered during the analysis. Any changes to the database after the lock can only be made by joint written agreement between the statistical team, the data manager and the study coordinator.

11. INVESTIGATOR RESPONSIBILITY

Except where the Principal Investigator's signature is specifically required, it is understood that the term "Investigator" as used in this protocol and on the CRFs refers to the Principal Investigator or a member of the staff that the Investigator designates to perform a certain duty under this protocol. The Investigator is ultimately responsible for the conduct of all aspects of the study.
For all other relevant Investigator responsibilities see "CPMP/ICH/135/95 Topic E6 - Guideline for Good Clinical Practice", Chapter 4.

12. ADMINISTRATIVE PROCEDURES

12.1 *Regulatory Authorities and Ethical Review Committee*
This study will be reported to the respective National Health Authority.
The clinical protocol will be submitted for approval to the relevant Independent Ethic Committee (IEC) or Institutional Review Board (IRB) before patients can be enrolled.
Copy of the IEC/IRB approval will be transmitted from the Investigator to the Sponsor before starting the study.

12.2 *Informed Consent*
All interviews will be conducted in the native language of the patients by the study personnel. Consent forms in the local language will be provided to the parents or guardians for their review (see Appendix IV). The parents or guardians will be asked to sign (or thumb-print whenever the parents/guardians are illiterate) consent to participate in a research study. The informed consent will describe the purpose of the study, the procedures to be followed, and the risks and benefits of participation. If a parent or guardian is unable to read or write, a signature from a witness to the informed consent discussion will be obtained. Parents or guardians will be informed that participation in the study is completely voluntary and that they may withdraw their child from the study at any time without any negative consequences.

12.3 *Confidentiality and Publication of Results*
All study documents are provided by the Sponsor in confidence to the investigators and his/her appointed staff. None of this material may be disclosed to any part not directly involved in the study without written permission from the Institute of Tropical Medicine, Antwerp.
Presentation and publication of the study results will be carried out by Investigators jointly with the Sponsor that will be informed at least 3 months before disclosure of the data, in order to discuss the content of the presentation or manuscript.

12.4 *Protocol Amendments*
Once the final clinical protocol has been issued and signed by the Investigator and the authorised signatories, it must not be informally altered. Clinical protocol amendments are alterations to a legal document (the clinical protocol) and have the same legal status and must pass through the appropriate steps before being implemented. In general, any change must be approved by the IEC prior to be effective. Administrative changes need only notification to the IEC without approval.
Any subsequent amendments must be made on separate sheet and must pass through the approval process. It must be clear to the Investigator that s/he can not change the clinical protocol without prior discussion with the Sponsor, which should give its approval.

12.5 Insurance
A liability insurance has been taken and will cover all trial sites.

13 STUDY COMMITTEE

13.1 *Consortium Secretariat*
The Consortium Secretariat (CS) acts as a steering committee. It comprises at least one investigator from each partner and will assess the progress of the trial. The members of the CS will address policy and operational issues related to the protocol. The CS has responsibility for protecting the scientific conduct and integrity of the trial. Its functions include:

- Review of the protocol before ethic committee approval,

- Formulation of recommendation for any change in the design and operations of the trial during the course of the trial, when needed,
- Exclusion of patients from the per protocol analysis.

The members of the CS are specified in the Consortium Agreement document prepared following the first face-to-face meeting held in Antwerp in February 2006.

13.2 ***Data Safety and Monitoring Board***
A Data and Safety Monitoring Board (DSMB) will be established for the purpose of providing an independent advice on safety of the treatments tested. The DSMB will be composed of three members with expertise in malaria, biostatistics and other appropriate disciplines. The DSMB will function as an independent body: it will regularly review interim analysis reports and will be informed by the sponsor on any serious adverse event occurring during the trials. The DSMB will be able to make decisions on whether the trial, or arms of the trial, need to be stopped. A Trial Monitor will inspect the site and trial documents/records.

The members of the DMB will be identified prior to enrolling the first patient.

14. ETHICAL ISSUES

AQ+AS and AL have been successfully used for the treatment of falciparum malaria in Phase III studies though the former in a non-coformulated form[6,7]. AL is widely used to treat uncomplicated malaria in Africa. DHAPQ has been used for several years in Asia, including safety studies in children. Recent trials in Rwanda and Peru (initiated and followed up by the ITM, Belgium) do not show any specific safety problem[8]. The combination is well tolerated. Chlorproguanil-dapsone has been used in young children, it is well tolerated and most of the serious adverse events recorded were more likely to be disease-related than drug-related[9]. Dapsone is known to cause methaemoglobinaemia and haemolysis (especially in G6PD deficiency). Clinically significant increases in methaemoglobin were not seen in SP-treated patients, but were observed in 7% of those given chlorproguanil-dapsone (all mild cases and 1 moderate case, all of limited duration)[9]. None of the children showed dyspnoea, the main symptom of high methaemoglobinaemia, and no action was required by attending physicians. Severe hemolysis is of concern because it is a feature of falciparum malaria, making it difficult to identify cases of drug-induced haemolysis. G6PD-deficient children in the chlorproguanil-dapsone group were more likely than similar patients in the SP group to have a $\geq$ 2g/dL Hb drop. However, by day 14 Hb values in both groups had been restored to baseline[9]. Larger Hb falls after treatment with CD than after SP have already been reported. There is no reason to think that the addition of artesunate will increase such risk. Nevertheless, children receiving CDA will be carefully monitored and Hb tested every day until day 3 after treatment. If needed, blood transfusion can be rapidly done in all sites where CDA is tested. Moreover, the recruitment of children having an Hb $\geq$ 7g/dL guarantees that, even if there is a drop, it will not reach life-threatening values.
The study will be presented for ethical clearance to the local ethical committees where the study is carried out and to the corresponding committees of the northern institutions directly involved in a specific site. Prior to the start of the project, the study will be explained to the communities involved. Written informed consent will be obtained from the guardians for all children before entering the study.

15. REFERENCES

1. AO. Talisuna, P. Bloland, U. D'Alessandro (2004) History, dynamics and public health importance of malaria parasite resistance. *Clinical Microbiology Review*. **17**:235-254.
2. International artemisinin group. (2004) Aretsunate combinations for treatment of malaria: meta-analysis. *Lancet*. 363:9-17.
3. The East African Network for Monitoring Antimalarial Treatment (EANMAT). (2003) The efficacy of antimalarial monotherapies, sulfadoxine-pyrimethamine and amodiaquine in East Africa: implications for subregional policy. *Tropical Medicine and International Health*.**8**:860-867.
4. Mutabingwa T, Nzila A, Mberu E, Nduati E, Winstanley P, Hills E, Watkins W, 2001. Chlorproguanil-dapsone for treatment of drug-resistant falciparum malaria in Tanzania. *Lancet* **358**: 1218-23.
5. Talisuna AO, Langi P, Mutabingwa TK, Van Marck E, Speybroeck N, Egwang T, Watkins WW, Hastings IM, D'Alessandro U. (2003) Intensity of transmission and spread of gene mutations linked to chloroquine and sulphadoxine-pyrimethamine resistance in falciparum malaria. *Int J Parasitol.* **33**: 1051-1058.
6. CE. Rwagacondo, C. Karema, V. Mugisha, A. Erhart, JC. Dujardin, C. Van Overmeir, P. Ringwald, U. D'Alessandro (2004) Is amodiaquine failing in Rwanda? Efficacy of amodiaquine alone or combined with artesunate in children with uncomplicated malaria. *Trop. Med. Int. Health*. **9**: 1091-1098.
7. WH. Wernsdorfer (2004) Coartemether (artemether and lumefantrine): an oral antimalarial drug. *Expert Rev. Anti-Infect. Ther*. **2**: 181-196.
8. C. Karema, CI. Fanello, C. Van Overmeir, JC. Dujardin, JP. Van geertruyden, W. Van Doren, D. Ngamije, U. D'Alessandro. Safety and efficacy of dihydroartemisinin-piperaquine (Artekin®) for the treatment of uncomplicated *Plasmodium falciparum* malaria in Rwandan children. *Trans. R. Soc. Trop. Med. Hyg.* (in press).
9. A. Alloueche, W. Bailey, S. Barton, J. Bwika, P. Chimpeni, CO. Falade, FA. Fehintola, J. Horton, S. Jaffar, T. Kanyok, PG. Kremsner, JG. Kublin, T. Lang, MA. Missinou, C. Mkandala, AMJ. Oduola, Z. Premji, L. Robertson, A. Sowunmi, SA. Ward, PA. Winstanley (2004) Comparison of chlorproguanil-dapsone with sulfadoxine-pyrimethamine for the treatment of uncomplicated falciparum malaria in young African children: double-blind randomized controlled trial. *Lancet*. **363**: 1843-1848.
10. Winstanley P, (2001). Chlorproguanil-dapsone (LAPDAP) for uncomplicated falciparum malaria. *Trop. Med. Int. Health* **6**: 952-4.
11. Sulo J, Chimpeni P, Hatcher J, Kublin JG, Plowe CV, Molyneux ME, Marsh K, Taylor TE, Watkins WM, Winstanley PA, (2002). Chlorproguanil-dapsone versus sulfadoxine-pyrimethamine for sequential episodes of uncomplicated falciparum malaria in Kenya and Malawi: a randomised clinical trial. *Lancet* **360**: 1136-43.
12. Lang T, Greenwood B, (2003). The development of Lapdap, an affordable new treatment for malaria. *Lancet Infect. Dis.* **3**: 162-8.
13. Pincock S, (2003). Drug company to offer new malaria drug cheaply in Africa. *BMJ* **327**: 360.
14. Watkins WM, Mberu EK, Winstanley PA, Plowe C, (1997). The efficacy of antifolate combinations in Africa: a predictive model based on pharmacodynamic and pharmacokinetic analyses. *Parasitology Today* **13**: 459-464.
15. Dollery C, 1999. Dapsone. Dollery C, ed. *Therapeutic drugs*. London, New York, Philadelphia, San Fransisco, Sydney, Toronto: Churchill Livingstone Edinburgh, D13 - D18.

16. Pirmohamed M, Park BK, (2001). Genetic susceptibility to adverse drug reactions. *Trends Pharmacol Sci* **22**: 298-305.
17. Jollow DJ, McMillan DC, (2001). Oxidative stress, glucose-6-phosphate dehydrogenase and the red cell. *Adv. Ex.p Med. Biol.* **500**: 595-605.
18. Mookherjee, S, Howard, V, Nzila-Mouanda, A, Watkins, W, Sibley, CH. (1999) Identification and analysis of dihydrofolate reductase alleles from Plasmodium falciparum present at low frequency in polyclonal patient samples. *Am. J. Trop. Med. Hyg.* **61**: 131-40.
19. Krudsood, S, Imwong, M, Wilairatana, P, Pukrittayakamee, S, Nonprasert, A, Snounou, G, White, NJ, Looareesuwan, S. (2005) Artesunate-dapsone-proguanil treatment of falciparum malaria: genotypic determinants of therapeutic response. *Trans. R. Soc. Trop. Med. Hyg.* **99**: 142-9.
20. Omari, A. A., Preston, C. and Garner, P.Artemether-lumefantrine for treating uncomplicated falciparum malaria.*Cochrane Database Syst. Rev. 2, CD003125, 2003*
21. Vugt, M. V., Wilairatana, P., Gemperli, B., Gathmann, I., Phaipun, L., Brockman, A., Luxemburger, C., White, N. J., Nosten, F. and Looareesuwan, S. (1999) Efficacy of six doses of artemether-lumefantrine (benflumetol) in multidrug-resistant Plasmodium *falciparum* malaria. *Am. J. Trop. Med. Hyg.* **60**: 936-942.
22. van Vugt, M., Looareesuwan, S., Wilairatana, P., McGready, R., Villegas, L., Gathmann, I., Mull, R., Brockman, A., White, N. J. and Nosten, F.(2000) Artemether-lumefantrine for the treatment of multidrug-resistant falciparum malaria.*Trans. R. Soc. Trop. Med. Hyg.* **94**: 545-548.
23. Falade, C, Makanga, M, Premji, Z, Ortmann, CE, Stockmeyer, M, de Palacios, PI. (2005) Efficacy and safety of artemether-lumefantrine (Coartem) tablets (six-dose regimen) in African infants and children with acute, uncomplicated falciparum malaria. *Trans. R. Soc.Trop. Med. Hyg.* **99**: 459-67.
24. Rwagacondo CE, Karema C, Mugisha V, Erhart A, Dujardin JC, Van Overmeir C, Ringwald P, D'Alessandro U. (2004) Is amodiaquine failing in Rwanda? Efficacy of amodiaquine alone and combined with artesunate in children with uncomplicated malaria. *Trop. Med. Int. Health.* **9**:1091-1098.
25. Denis, M. B., Davis, T. M., Hewitt, S., Incardona, S., Nimol, K., Fandeur, T., Poravuth, Y., Lim, C. and Socheat, D. (2002) Efficacy and safety of dihydroartemisinin-piperaquine (Artekin) in Cambodian children and adults with uncomplicated falciparum malaria. *Clin. Infect. Dis.* **35**: 1469-1476.

APPENDICES

APPENDIX I

Guidelines for Grading Patient Symptoms, signs and laboratory findings.

Table A. Guidelines for Grading Patient Symptoms.

	**Grade 1 MILD**	**Grade 2 MODERATE**	**Grade 3 SEVERE**	**Grade 4 LIFE THREATENING**
**Subjective fever in the past 24 h**	N/A	Present (Yes)	N/A	N/A
Weakness	Mild decrease in activity; For children – weak, but still playing	Moderate decrease in activity; For children – weak, and playing limited	Not participating in usual activities; For children – not playing	Prostration
**Muscle and/or joint aches***	Mild and/or localized complaints	Diffuse complaints	Objective weakness; function limited	N/A
**Headache***	Mild, no treatment required	Transient, moderate; treatment required	Severe, constant; requires narcotic therapy	Intractable; requires repeated narcotic therapy
**Anorexia**	Decreased appetite, but still taking solid food	Decreased appetite, avoiding solid food but taking liquids	Appetite very decreased; Refusing to breast feed, no solids or liquids taken (< 2 years ≤ 12 hr; > 2 years ≤ 24 hr)	Appetite very decreased; Refusing to breast feed, no solids or liquids taken (< 2 years > 12 hr; > 2 years > 24 hr)
**Nausea***	Mild, transient feeling of impending vomiting; maintains reasonable intake	Moderate and/or constant feeling of impending vomiting; intake decreased	Severe, constant feeling of impending emesis; intake decreased significantly	N/A
**Vomiting**	1 episode per day	2-3 episodes per day	Orthostatic hypotension or IV fluids required	Hypotensive shock or physiological consequences requiring IV fluid therapy
**Abdominal pain***	Mild (1-3 on a scale of 1 to 10)	Moderate (4-6 on a scale of 1 to 10)	Moderate to severe (≥ 7 on a scale of 1 to 10)	Severe – hospitalization for treatment
**Diarrhea**	Transient 3-4 loose stools/day	5-7 loose stools/day	Orthostatic hypotension or > 7 loose stools/day or IV fluids required	Hypotensive shock or physiological consequences requiring IV fluid therapy
**Cough**	Transient / intermittent	Persistent / constant	Uncontrolled	Cyanosis, stridor, severe shortness of breath
**Pruritis**	Transient pruritis	Pruritis that disturbs sleep	Severe, constant pruritis, sleep disturbed	N/A
**Tinnitus***	Mild, transient ringing or roaring sound	Moderate, persistent ringing or roaring sound	Severe ringing or roaring sound with associated hearing loss	N/A
**Behavioural changes**	Mild difficulty concentrating; mild confusion or agitation; activities of daily living unaffected; no treatment	Moderate confusion or agitation; some limitation of activities of daily living; minimal treatment	Severe confusion or agitation; Needs assistance for activities of daily living; therapy required	Toxic psychosis; hospitalization for treatment
**"Flu" (viral URI)**	Mild nasal congestion, mild rhinorrhea	Moderate nasal congestion, moderate rhinorrhea	N/A	N/A
**Allergic reaction**	N/A	N/A	Urticaria	Severe urticaria anaphylaxis, angioedema
**Convulsion**	N/A	N/A	Localized or generalized seizure	Status epilepticus
*** Assess only in children ≥ 3 years of age. Answer N/A for younger children and those unable to answer.**				

Reference – Based on WHO Toxicity Grading Scale for Determining the Severity of Adverse Events

Table B. Guidelines for Physical Examination

**Dehydration**	Assess skin touch and turgor, mucous membranes, eyes, crying, fontanelle, pulse, urine output
**Jaundice**	Assess for yellowing of the sclera. Also evaluate the palpebral conjunctiva, lips, and skin.
**Chest**	Observe the rate, rhythm, depth, and effort of breathing. Check the patient's colour for cyanosis.<br><br>The maximum acceptable respiratory rate by age: < 2 months = 60, 2-12 months = 50, 1-5 years = 40, above 5 years = 30.<br><br>Inspect the neck for the position of the trachea, for supraclavicular retractions, and for contraction of the sternomastoid or other accessory muscles during inspiration.<br><br>Auscultate the anterior and posterior chest for normal breath sounds and any adventitious sounds (crackles or rales, wheezes, and rhonchi). *Crackles are intermittent, non-musical, fine or coarse sounds that may be due to abnormalities of the lungs (pneumonia, fibrosis, early congestive heart failure) or airways (bronchitis or bronchiectasis). Wheezes are high-pitched and result from narrowed airways. Rhonchi are relatively low-pitched and suggest secretions in large airways.*<br><br>If abnormalities are identified, evaluate for transmitted voice sounds. In addition, palpate the chest to assess for tactile fremitus, and percuss the chest to assess for areas of dullness. *Normal, air-filled lungs emit predominantly vesicular breath sounds, transmit voice sounds poorly with "ee" = "ee", and have no tactile fremitus. Airless lung, as in lobar pneumonia, emits bronchial breath sounds, transmits spoken words clearly with "ee" = "aay" (egophany), and has an increase in tactile fremitus.*
**Abdomen**	Inspection and auscultation of the abdomen. Listen for bowel sounds in the abdomen before palpating it. Palpate the abdomen in all 4 quadrants lightly and then deeply. Assess the size of the liver and spleen. To assess for peritoneal inflammation, look for localised and rebound tenderness, and voluntary or involuntary rigidity.
**Skin**	Inspect the skin for colour, turgor, moisture, and lesions. If lesions are present, note their location and distribution (diffuse or localised), arrangement (linear, clustered, annular, dermatomal), type (macules, papules, vesicles) and colour.
**Tablet test**	For children ≥ 9 months of age, ask the patient to pick a tablet (or equivalent object) up off a flat surface using the thumb and index finger of their dominant hand. *This tests for co-ordination of the upper extremity assessing the function of the motor system, cerebellar system, vestibular system (for coordinating eye and body movements) and the sensory system, for position sense. When testing small children, be aware that they will likely attempt to put the object into their mouth.*

Table C. Grading Physical Examination Findings

	Grade 1<br>MILD	Grade 2<br>MODERATE	Grade 3<br>SEVERE	Grade 4<br>LIFE-THREATENING
**Temperature * (axillary)**	37.5-37.9°C	38.0-39.5°C	> 39.5°C	Sustained fever, equal or greater than 40.0°C for longer than 5 days
**Dehydration**	Less than 2 of the following:<br>Restless, irritable<br>Sunken eyes<br>Drinks eagerly, thirsty<br>Skin pinch goes back slowly	2 of the following:<br>Restless, irritable<br>Sunken eyes<br>Drinks eagerly, thirsty<br>Skin pinch goes back slowly	Two of the following:<br>Lethargic or unconscious<br>Sunken eyes<br>Not able to drink or drinking poorly<br>Skin pinch goes back very poorly	Two of the following + shock:<br>Lethargic or unconscious<br>Sunken eyes<br>Not able to drink or drinking poorly<br>Skin pinch goes back very poorly
**Jaundice**	Slight yellowing of sclera and conjunctiva	Moderate yellowing of sclera and conjunctiva, yellowing of mucous membranes	Severe yellowing of sclera and conjunctiva, yellowing of skin	N/A
**Chest**	Mildly increased RR (for age, temperature), transient or localised adventitious sounds	Moderately increased RR, diffuse or persistent adventitious sounds	Rapid RR (< 2 months > 60, 2-12 months > 50, 1-5 years > 40, adults > 30)* nasal flaring, retractions	Cyanosis
**Abdomen**	Normal bowel sounds, mild localised tenderness, and/or liver palpable 2-4 cm below the right costal margin (RCM), and/or spleen palpable, and/or umbilical hernia present	Normal or mildly abnormal bowel sounds, moderate or diffuse tenderness; and/or mild to moderately enlarged liver (4-6 cm below the RCM) and/or spleen palpable up to half-way between umbilicus and symphysis pubis	Severely abnormal bowel sounds, severe tenderness to palpation. Evidence of peritoneal irritation and/or significant enlargement of liver (> 6 cm below the RCM) and/or spleen palpable beyond half-way between umbilicus and symphysis pubis	Absent bowel sounds. Involuntary rigidity
**Skin†**	Localised rash, erythema, or pruritis	Diffuse, maculopapular rash, dry desquamation	Vesiculation, moist desquamation, or ulceration	Exfoliative dermatitis, mucous membrane involvement or erythema multiforme or suspected Stevens-Johnson or necrosis requiring surgery

	Grade 1<br>MILD	Grade 2<br>MODERATE	Grade 3<br>SEVERE	Grade 4<br>LIFE-THREATENING
**Hearing**	*< 4 years: N/A*<br>≥ 4 years: Decreased hearing in one ear	*< 4 years: N/A*<br>≥ 4 years: Decreased hearing in both ears or severe impairment in one ear	*< 4 years: Any evidence of hearing impairment*<br>≥ 4 years: Severe impairment in both ears	N/A
**Tablet test**	Difficulty grasping tablet but able to pick up	Unable to pick up tablet without dropping	Unable to grasp tablet	N/A
**Clinical symptoms / sign *(not otherwise specified)***	No treatment required; monitor condition	Treatment required	Requires treatment and possible hospitalisation	Requires active medical intervention, hospitalisation, or hospice care

Reference – The Harriet Lane Handbook, 15th edition, 2000
† Reference – WHO Toxicity Grading Scale for Determining the Severity of Adverse Events

TABLE D. Guidelines for Grading of Laboratory Abnormalities

	Grade 1<br>MILD	Grade 2<br>MODERATE	Grade 3<br>SEVERE	Grade 4<br>LIFE-THREATENING
**Haemoglobin** *(g/dL)*	9.0 – 9.9	7.0 – 8.9	5.0 – 6.9	< 5.0

Reference – The Harriet Lane Handbook, 15th edition, 2000†
Reference – WHO Toxicity Grading Scale for Determining the Severity of Adverse Events

APPENDIX II*

Table A. Dihydroartemisinine-piperaquine (DHAPQ)
DHAPQ tablets are green film coated intended for oral use and contain 20/160mg or 40/320mg of dihydroartemisinin (DHA) and piperaquine phosphate (PQ) respectively.

DHAPQ 20/160 mg Tablets Components

Composition

One tablet contains

Component	Amount (mg)
Piperaquine Phosphate (ST 3073)	160
Dehydroartemisinine (ST 3074)	20
Starch Maize	34.5
Dextrin	26.4
Hydroxypropylmethylcellulose	2.4
Sodium carboxymethylcellulose	11.8
Magniesum Stereate	2.4
OY-31074 Opadry Green coating	7.5

The composition of OY-31074 Opadry coating preparation is

Component	(%w/w)
Hydroxypropylmethylcellulose	62.501
Titanium Dioxide	25.450
Macrogol 400	6.249
FD&Blue #1 Brilliant blue A1 lake	0.400
FD&Blue #2 Indigo Carmine A1 lake	0.400
FD&C Yellow #5 Tartrazine A1 lake	5.000

DHAPQ 40/320 mg Tablets Components

Composition
One tablet contains

Component	Amount (mg)
Piperaquine Phosphate (ST 3073)	320
Dehydroartemisinine (ST 3074)	40
Starch Maize	69
Dextrin	52.8
Hydroxypropylmethylcellulose	4.8
Sodium carboxymethylcellulose	23.6
Magniesum Stereate	4.8
OY-31074 Opadry Green coating	15

The composition of OY-31074 Opadry coating preparation is the same as above.

Dihydroartemisinin will be given daily. The number of tablets per day is reported in brackets. One tablet of DHAPQ (pediatric tablets) contains 20/40 mg of DHA and 160/320 mg of PPQ.

Weight in kg	mg of DHA to be given daily	Dose of DHA as mg/kg/d
5 - 6	10 (1/2 tablet with 20 mg)	1.67 - 2.5
7 - 12	20 (1 tablet with 20 mg)	1.67 - 2.86
13 - 23	40 (1 tablet with 40 mg)	1.74 - 3.08
24 - 35	80 (2 tablets with 40 mg)	2.29 - 3.33

One tablet of DHAPQ contains 40 mg of DHA and 320 mg of PPQ for adult patients.

Piperaquine will be given daily. The number of tablets per day is reported in brackets. One tablet of DHAPQ (pediatric tablets) contains 20/40 mg of DHA and 160/320 mg of PPQ.

Weight in kg	mg of PPQ to be given daily	Dose of PPQ as mg/kg/d
5 - 6	80 (1/2 tablet with 160 mg)	13.36 - 20
7 - 12	160 (1 tablet with 160 mg)	13.36 – 22.88
13 - 23	320 (1 tablet with 320 mg)	13.92 – 24.64
24 - 35	640 (2 tablets with 320 mg)	18.32 – 26.4

One tablet of DHAPQ contains 40 mg of DHA and 320 mg of PPQ for adult patients.

Table B: Coartem® dose based on body weight will be given daily. Tablets containing 20 mg of Artemether and 120 mg of Lumefantrine.

Weight in kg	Number of tablet per dose
5 to < 15 kg	1 tablet per dose
15 to < 25 kg	2 tablets per dose
25 to < 35 kg	3 tablets per dose

Table C. Chlorproguanil-dapsone-artesunate (CDA)

CDA
Description
CDA tablets are peanut shaped orange or yellow film coated tablets intended for oral use and contain 12/15/24mg or 60/75/120mg of Chlorproguanil Hydrochloride, Dapsone and Artesunate, respectively.

Composition
CDA **12/15/24 mg** Tablets Component Quantity[mg/tablet]
Chlorproguanil HCl 12.00
Dapsone 15.00
Artesunate 24.00
Microcrystalline Cellulose 48.20
Hypromellose 5.70
Mannitol 70.40
Croscarmellose Sodium 8.05
Magnesium Stearate 1.75
Opadry Yellow 03B267162 8.70
Total Tablet Weight 193.80

Notes: The actual quantity of drug substance may be adjusted depending on the purity of the input drug substance 2. Opadry Brown contains Titanium Dioxide Ph.Eur., Hypromellose Ph.Eur., Macrogol/PEG 400 Ph.Eur., Iron Oxide Yellow and Iron Oxide Red.

Dosage

Weight in Kg	Tablets/day
5 - <7	1
7 - <10	1.5
10 - <13	2

CDA **60/75/120 mg** Tablets Component Quantity[mg/tablet]

Chlorproguanil HCl 60.00
Dapsone 75.00

Artesunate 120.00
Microcrystalline Cellulose 55.35
Hypromellose 16.65
Mannitol 87.80
Croscarmellose Sodium 19.80
Magnesium Stearate 4.30
Opadry Yellow 03B221332 20.63
Total Tablet Weight 459.53

Notes:
1. The actual quantitiy of drug substance may be adjusted depending on the purity of the input drug substance 2. Opadry yellow contains Titanium Dioxide Ph.Eur., Hypromellose Ph.Eur., Macrogol/PEG 400 Ph.Eur., Iron Oxide Yellow and Iron Oxide Red.

Dosage

**Weight in Kg**	**Tablets/day**
13 - <19 kg	0.5
19 - <31 kg	1
31 - <46 kg	1.5
≥ 46 kg	2

.

Table D. Amodiaquine-artesunate (AQAS) (COARSUCAM™)

Description: Round tablet, yellow on one side and white-slightly yellow on the other, with a breaking bar, AS engraved on one side and either 25, 50 or 100 on the other side.

Formulation:

3 dosages As/Aq	25mg/67.5mg	50mg/135mg	100mg/270mg
Artesunate	25.000 mg	50.000 mg	100.000 mg
Amodiaquine hydrochloride	88.160 mg(1)	176.320 mg(2)	352,640 mg(3)
Excipients QSP	175.000 mg	350.000 mg	700.000 mg

(1) corresponds to 67.50 mg of amodiaquine base
(2) corresponds to 135.00 mg of amodiaquine base
(3) corresponds to 270.00 mg of amodiaquine base

The dose should be adapted to the body weight as follows: between 2 and 10 mg/kg of artesunate and 7.5 to 15 mg/kg of amodiaquine once a day for 3 days.

Weight	Formulation	Tablets/day
<9 Kg	COARSUCAM™<br>25 mg/67,5 mg	1
9-17.9Kg	COARSUCAM ™<br>50 mg/135 mg	1
18-35.9 Kg	COARSUCAM ™<br>100 mg/270 mg	1

APPENDIX III

WORLD MEDICAL ASSOCIATION DECLARATION OF HELSINKI

Ethical Principles for Medical Research Involving Human Subjects

Recommendations guiding medical physicians
in biomedical research involving human subjects

Adopted by the 18th WMA General Assembly
Helsinki, Finland, June 1964
and amended by the
29th WMA General Assembly, Tokyo, Japan, October 1975
35th WMA General Assembly, Venice, Italy, October 1983
41st WMA General Assembly, Hong Kong, September 1989
48th WMA General Assembly, Somerset West, Republic of South Africa, October 1996
and the
52nd WMA General Assembly, Edinburgh, Scotland, October 2000
Note of Clarification on Paragraph 29 added by the WMA General Assembly, Washington 2002.

A. INTRODUCTION

1. The World Medical Association has developed the Declaration of Helsinki as a statement of ethical principles to provide guidance to physicians and other participants in medical research involving human subjects. Medical research involving human subjects includes research on identifiable human material or identifiable data.

2. It is the duty of the physician to promote and safeguard the health of the people. The physician's knowledge and conscience are dedicated to the fulfillment of this duty.

3. The Declaration of Geneva of the World Medical Association binds the physician with the words, "The health of my subject will be my first consideration," and the International Code of Medical Ethics declares that, "A physician shall act only in the subject's interest when providing medical care which might have the effect of weakening the physical and mental condition of the subject."

4. Medical progress is based on research which ultimately must rest in part on experimentation involving human subjects.

5. In medical research on human subjects, considerations related to the well-being of the human subject should take precedence over the interests of science and society.

6. The primary purpose of medical research involving human subjects is to improve prophylactic, diagnostic and therapeutic procedures and the understanding of the etiology and pathogenesis of disease. Even the best proven prophylactic, diagnostic, and therapeutic methods must continuously be challenged through research for their effectiveness, efficiency, accessibility and quality.

7. In current medical practice and in medical research, most prophylactic, diagnostic and therapeutic procedures involve risks and burdens.

8. Medical research is subject to ethical standards that promote respect for all human beings and protect their health and rights. Some research populations are vulnerable and need special protection. The particular needs of the economically and medically disadvantaged must be recognized. Special attention is also required for those who cannot give or refuse consent for themselves, for those who may be subject to giving consent under duress, for those who will not benefit personally from the research and for those for whom the research is combined with care.

9. Research Investigators should be aware of the ethical, legal and regulatory requirements for research on human subjects in their own countries as well as applicable international requirements. No national ethical, legal or regulatory requirement should be allowed to reduce or eliminate any of the protections for human subjects set forth in this Declaration.

B. BASIC PRINCIPLES FOR ALL MEDICAL RESEARCH

10. It is the duty of the physician in medical research to protect the life, health, privacy, and dignity of the human subject.

11. Medical research involving human subjects must conform to generally accepted scientific principles, be based on a thorough knowledge of the scientific literature, other relevant sources of information, and on adequate laboratory and, where appropriate, animal experimentation.

12. Appropriate caution must be exercised in the conduct of research which may affect the environment, and the welfare of animals used for research must be respected.

13. The design and performance of each experimental procedure involving human subjects should be clearly formulated in an experimental protocol. This protocol should be submitted for consideration, comment, guidance, and where appropriate, approval to a specially appointed ethical review committee, which must be independent of the Investigator, the Sponsor or any other kind of undue influence. This independent committee should be in conformity with the laws and regulations of the country in which the research experiment is performed. The committee has the right to monitor ongoing trials. The researcher has the obligation to provide monitoring information to the committee, especially any serious adverse events. The researcher should also submit to the committee, for review, information regarding funding, Sponsors, institutional affiliations, other potential conflicts of interest and incentives for subjects.

14. The research protocol should always contain a statement of the ethical considerations involved and should indicate that there is compliance with the principles enunciated in this Declaration.

15. Medical research involving human subjects should be conducted only by scientifically qualified persons and under the supervision of a clinically competent medical person. The responsibility for the human subject must always rest with a medically qualified person and never rest on the subject of the research, even though the subject has given consent.

16. Every medical research project involving human subjects should be preceded by careful assessment of predictable risks and burdens in comparison with foreseeable benefits to the subject or to others. This does not preclude the participation of healthy volunteers in medical research. The design of all studies should be publicly available.

17. Physicians should abstain from engaging in research projects involving human subjects unless they are confident that the risks involved have been adequately assessed and can be satisfactorily managed. Physicians should cease any investigation if the risks are found to outweigh the potential benefits or if there is conclusive proof of positive and beneficial results.

18. Medical research involving human subjects should only be conducted if the importance of the objective outweighs the inherent risks and burdens to the subject. This is especially important when the human subjects are healthy volunteers.

19. Medical research is only justified if there is a reasonable likelihood that the populations in which the research is carried out stand to benefit from the results of the research.

20. The subjects must be volunteers and informed participants in the research project.

21. The right of research subjects to safeguard their integrity must always be respected. Every precaution should be taken to respect the privacy of the subject, the confidentiality of the subject's information and to minimize the impact of the study on the subject's physical and mental integrity and on the personality of the subject.

22. In any research on human beings, each potential subject must be adequately informed of the aims, methods, sources of funding, any possible conflicts of interest, institutional affiliations of the researcher, the anticipated benefits and potential risks of the study and the discomfort it may entail. The subject should be informed of the right to abstain from participation in the study or to withdraw consent to participate at any time without reprisal. After ensuring that the subject has understood the information, the physician should then obtain the subject's freely-given informed consent, preferably in writing. If the consent cannot be obtained in writing, the non-written consent must be formally documented and witnessed.

23. When obtaining informed consent for the research project the physician should be particularly cautious if the subject is in a dependent relationship with the physician or may consent under duress. In that case the informed consent should be obtained by a well-informed physician who is not engaged in the investigation and who is completely independent of this relationship.

24. For a research subject who is legally incompetent, physically or mentally incapable of giving consent or is a legally incompetent minor, the Investigator must obtain informed consent from the legally authorized representative in accordance with applicable law. These groups should not be included in research unless the research is necessary to promote the

health of the population represented and this research cannot instead be performed on legally competent persons.

25. When a subject deemed legally incompetent, such as a minor child, is able to give assent to decisions about participation in research, the Investigator must obtain that assent in addition to the consent of the legally authorized representative.

26. Research on individuals from whom it is not possible to obtain consent, including proxy or advance consent, should be done only if the physical/mental condition that prevents obtaining informed consent is a necessary characteristic of the research population. The specific reasons for involving research subjects with a condition that renders them unable to give informed consent should be stated in the experimental protocol for consideration and approval of the review committee. The protocol should state that consent to remain in the research should be obtained as soon as possible from the individual or a legally authorized surrogate.

27. Both authors and publishers have ethical obligations. In publication of the results of research, the Investigators are obliged to preserve the accuracy of the results. Negative as well as positive results should be published or otherwise publicly available. Sources of funding, institutional affiliations and any possible conflicts of interest should be declared in the publication. Reports of experimentation not in accordance with the principles laid down in this Declaration should not be accepted for publication.

C. ADDITIONAL PRINCIPLES FOR MEDICAL RESEARCH COMBINED WITH MEDICAL CARE

28. The physician may combine medical research with medical care, only to the extent that the research is justified by its potential prophylactic, diagnostic or therapeutic value. When medical research is combined with medical care, additional standards apply to protect the subjects who are research subjects.

29. The benefits, risks, burdens and effectiveness of a new method should be tested against those of the best current prophylactic, diagnostic, and therapeutic methods. This does not exclude the use of placebo, or no treatment, in studies where no proven prophylactic, diagnostic or therapeutic method exists.

30. At the conclusion of the study, every subject entered into the study should be assured of access to the best proven prophylactic, diagnostic and therapeutic methods identified by the study.

31. The physician should fully inform the subject which aspects of the care are related to the research. The refusal of a subject to participate in a study must never interfere with the subject-physician relationship.

32. In the treatment of a subject, where proven prophylactic, diagnostic and therapeutic methods do not exist or have been ineffective, the physician, with informed consent from the subject, must be free to use unproven or new prophylactic, diagnostic and therapeutic

measures, if in the physician's judgment it offers hope of saving life, re-establishing health or alleviating suffering. Where possible, these measures should be made the object of research, designed to evaluate their safety and efficacy. In all cases, new information should be recorded and, where appropriate, published. The other relevant guidelines of this Declaration should be followed.

FOOTNOTE: Note of Clarification on Paragraph 29 of the WMA Declaration of Helsinki

The WMA hereby reaffirms its position that extreme care must be taken in making use of a placebo-controlled trial and that in general this methodology should only be used in the absence of existing proven therapy. However, a placebo-controlled trial may be ethically acceptable, even if proven therapy is available, under the following circumstances:

Where for compelling and scientifically sound methodological reasons its use is necessary to determine the efficacy or safety of a prophylactic, diagnostic or therapeutic method; or

Where a prophylactic, diagnostic or therapeutic method is being investigated for a minor condition and the patients who receive placebo will not be subject to any additional risk of serious or irreversible harm.

APPENDIX IV
RESEARCH PARTICIPANT INFORMED CONSENT FORM

STUDY TITLE

Evaluation of 4 artemisinin-based combinations for treating uncomplicated malaria in African children

- Multicentre Study In Africa -

Please read the background information and informed consent form carefully. The background information explains your rights and our responsibilities to you. If you have any questions concerning the study please do not hesitate to ask any of the doctors. Before you decide, it is important for you to understand why the research is being done and what it will involve. You will be given a copy of this signed document (Informed Consent Form) to take home with you.

YOU MUST KEEP THIS BACKGROUND INFORMATION WITH YOU THROUGHOUT THE STUDY PERIOD.

PURPOSE OF THE STUDY

This research study is being done to learn more about the treatment of malaria. We are carrying out a research study to compare different medicines for the treatment of mild malaria. The medicines we are studying are: dihydroartemisinin-piperaquine, arthemeter-lumefantrine, chlorproguanil-dapsone-artesunate and amodiaquine-artesunate. All these drugs are active against malaria. With this study we want to find out their relative value in curing malaria in terms of efficacy and safety. Five thousand and ten patients from seven different African countries will participate in this study. Each country will test 3 of the medicines mentioned above.

HOW THE STUDY IS DONE

The child under your care will be treated for malaria with one of the above study medicines. After the treatment, your child will be actively followed for 28 days to see if the malaria infection is completely cured. If your child is not completely cured by the study medicines, s/he will then be given treatment according to the standard practice in your country. The study

medicine that your child will receive will be determined by a process of randomization. Randomization means that your child will receive one of the three medicines studied in your country by chance. You are being asked to allow your child (child under your care in the case of a legal guardian), to participate in this study. Your child will be actively followed up for 28 days or until such time as you or the study doctors decide that your child should no longer participate in the study. Afterwards, you will be asked to attend the health facility for the next 6 months any time your child is sick where s/he will be examined by the study doctor and treated accordingly. However, if your child has malaria again, s/he will be treated with the same drug used for the previous episode and actively followed up again for the next 28 days after treatment. You can choose to withdraw your consent to participate in the study any time and without influencing the medical attention your child may need.

The study may be discontinued by the sponsor at any time, and for any reason.

PROCEDURES

1) The study doctors will examine your child today.
2) A blood sample will be collected. A small amount of blood will be taken by fingerprick to examine for malaria parasites, to measure the blood count, to store blood samples on filter paper for future laboratory tests that will not impact on the health care of your child.
3) If the diagnosis of malaria is confirmed, and your child is eligible for the study, treatment with either dihydroartemisinin + piperaquine (DHA+PPQ), arthemeter + lumefantrine (A+L), chlorproguanil-dapsone-artesunate (CDA) or amodiaquine-artesunate. (AQ+AS) will be given at the clinic during the first 3 days. This means that your child will be hospitalised the first 3 days of the study.
4) You will be asked to return to the clinic at least 7 more times over the next month so that the success of the treatment can be judged. At each of the follow-up visits, your child will be examined by the study doctors and, a small amount of blood will taken by fingerprick to examine for malaria parasites and to save on filter paper.
5) If case of missing appointment, the home health visitor will visit your child at your home to find out why you missed the appointment and bring your child to the clinic for assessment.
6) If, at any time, the treatment given to your child does not seem to be working well,

it will be changed to treatment according to the usual standard of care.

7) There will be someone at the study clinic every day from 8:00 am to 5:00 pm and at night. You can come to the clinic for evaluation anytime that your child is ill during the next 28 days.

8) For the haematology there will be at least 5 blood samples: one before the first dose at D0, the second one at D3, the third at D7, i.e. one week after D0, the forth at D14, i.e. two weeks after D0, the fifth at D28, i.e. four weeks after D0. In health centres where CDA is studied, 2 additional samples for haematology will be taken at D1 and D2. The amount of blood collected for each sample will be less than 1mL.

9) For biochemistry, 3 samples will be collected: one before the first dose at D0, the second at D7, i.e. one week after D0 and the third at D28, i.e. four weeks after D0. An additional sample will be collected at D14 if the results at D7 are outside the normal ranges. Each sample will be of 1 mL.

10) Blood samples will be collected from an arm vein or from a finger by an experienced nurse. Blood sampling may cause pain and swelling. In addition small violet spots around the site of injection called hematoma could appear.

RISKS AND DISCOMFORTS

1) Side effects following treatment with the study medications could occur. Generally, side effects (nausea, headache, dizziness…) are expected to be mild and short-lived.

   Your child will be monitored closely after receiving treatment for malaria with the study medications for any possible side effects of the drugs and will receive appropriate medical care for any problem that happens during the course of the study.

2) The medicine CDA contains chlorproguanil-dapsone (CD). This has been previously evaluated in Africa and it is commercialised in several countries. Some serious adverse events have been observed in one or more children out of 100 treated with CD but the frequency is not higher than that for another drug commonly used against malaria, sulfadoxine-pyrimethamine. In a minority of patients the treatment with CD is followed by anaemia (reduction of red blood cells) that disappears after 2 weeks. This is in part due to the destruction of the malaria parasites in the blood cells. Sometimes anaemia can be associated to the deficiency of an enzyme, Glucose-6-Phosphate Dehydrogenase (G6PD). In this study your child will be carefully followed to promptly detect any anaemia that will be immediately treated. Previous studies have not shown any difference between children aged 1 year or more and adults. However, studies on animals have found that young rats from their 4th day of life are more sensitive to CDA and chlorproguanil than adult rats. This is why CDA will not be given to children aged less than 1 year, until additional data are available.

3) Randomization: Your child will be assigned to a treatment group by chance. The treatment your child receives may prove to be less effective or to have more side effects than the other study treatments or than other available treatments. This will not be known until after the study is completed.

4) Severe malaria: Your child may develop malaria that is severe even after receiving treatment with study medications. If your child shows any evidence of severe malaria (including persistent vomiting, low blood (anaemia), convulsions, confusion, or coma) treatment with the usual standard of care will be given and your child will be referred for possible admission to hospital.
5) Blood draws: The risks of drawing blood from a fingerprick include temporary discomfort from the needle stick, bruising, skin infection, and fainting. The amount of blood removed will be too small to affect your child's health.
6) Unknown Risks: The research treatments may have side effects that no one knows about yet. The researchers will let you know if they learn anything that might make you change your mind about your child's participation in the study.
7) Confidentiality: Participation in research may involve a loss of privacy, but information about your child will be handled as confidentially as possible. Medical information related to malaria will be collected on your child and only people taking care of the child and/or the study personnel will have access to this information. Records will be kept as confidential as possible. You will also have the right to request and see the information collected during this study on your child.

BENEFITS

1) The potential benefit to your child is that the treatment received may prove to be more effective than the other study treatments or than other available treatments, although this cannot be guaranteed.
2) Your child will receive clinical care from the medical officers and nurses of the project staff in the study clinic. This will include care for unscheduled sick visits.
3) The knowledge gained from this study will help your country in determining the best treatment for uncomplicated malaria.

COST/PAYMENT

After enrolment in the study, you will not be charged for clinic visits or treatment. Your child will not be paid for participation in the study. We will reimburse any transport costs incurred for clinic visits and meals will be provided when your child is admitted for observation and treatment administration.

ALTERNATIVES TO PARTICIPATION

Your child's participation in this study is completely voluntary. If you decide that you do not want to participate in the study or decide to withdraw your child from the study at any time and for any reason, this will not affect your child's care at the outpatient department, where standard care for all medical problems is available. During the study, you will be informed promptly of any new information that may influence your willingness to continue participation in the study.

CONSEQUENCES OF WITHDRAWAL

Should you decide to withdraw your child from the study before your child has finished the course of study medicines, then your child will receive the local standard treatment for malaria from the study team, but after the standard treatment has been given, medical care will no longer be provided by the study team. If the child is withdrawn from the study after completion of the course of study medicines, then no further care will be provided by the study team.

USE OF THE RESULTS

The findings from this study may be published in a medical journal. The study participants will not be identified by name. After the study is completed, you may request an explanation of the study results.

TREATMENT AND COMPENSATION FOR INJURY

If you are injured or have questions about injuries as a result of being in the study, please contact the doctors in the study clinic. The services at the public health facility will be open to you in case of any such injury.

VOLUNTARY PARTICIPATION

Participation in this study is entirely voluntary. You have the right to refuse your child's participation or to withdraw at any point in this study without negative consequences or loss of benefits to which you and your child are otherwise entitled.

implication of your SIGNATURE OR THUMBPRINT

If you give consent for your child to participate in this study, you should sign or place your thumbprint in the consent form. Your signature or thumbprint below means that you understand the information given to you about your child's participation in the study and in the consent form. You will also be asked to sign another copy of this informed consent form for documentation.

CONSENT FORM

CONSENT FORM FOR PARTICIPATION IN RESEARCH PROJECTS AND CLINICAL TRIALS

Study Title

Evaluation of 4 artemisinin-based combinations for treating uncomplicated malaria in African children

- Multicentre Study In Africa -

Local Investigator: ____

Address:____

Contact number____

I, …………………………………. mother/father/legal representative declare that I have understood the objectives and purposes of this study. I agree that my child………………………………………………………… participates in this study.

I am aware that I can withdraw my child from the study at any time without any consequence to my child or to me.

____
Name of parent/legal representative

____
Signature or Thumbprint * of parent/ legal representative Date/Time

*If the parent or guardian is unable to read and/or write, an impartial witness should be present during the informed consent discussion. After the written informed consent form is read and explained to the parent or guardian, and after they have orally consented to their child's participation in the trial, and have either signed the consent form or provided their fingerprint, the witness should sign and personally date the consent form. By signing the consent form, the witness attests that the information in the consent form and any other written information was accurately explained to, and apparently understood by, the parent or guardian, and that informed consent was freely given by the parent or guardian.

Name of Person Witnessing Consent (printed)

Signature of Person Witnessing Consent Date/Time

APPENDIX V

Criteria for Severe Malaria/Danger Signs

Severe Malaria

- Unarousable coma *(if after convulsion, > 30 min)*
- Repeated convulsions *(> 2 within 24 h)*
- Severe anaemia *(Hb < 5.0 g/dL)*
- Respiratory distress *(laboured breathing at rest)*

Danger Signs

- Recent convulsions *(>1 within 24 h)*
- Altered consciousness *(confusion)*
- Lethargy
- Unable to drink or breast feed
- Vomiting everything
- Unable to stand/sit due to weakness

APPENDIX VI. PARTICIPANT SELECTION AND ENROLLMENT

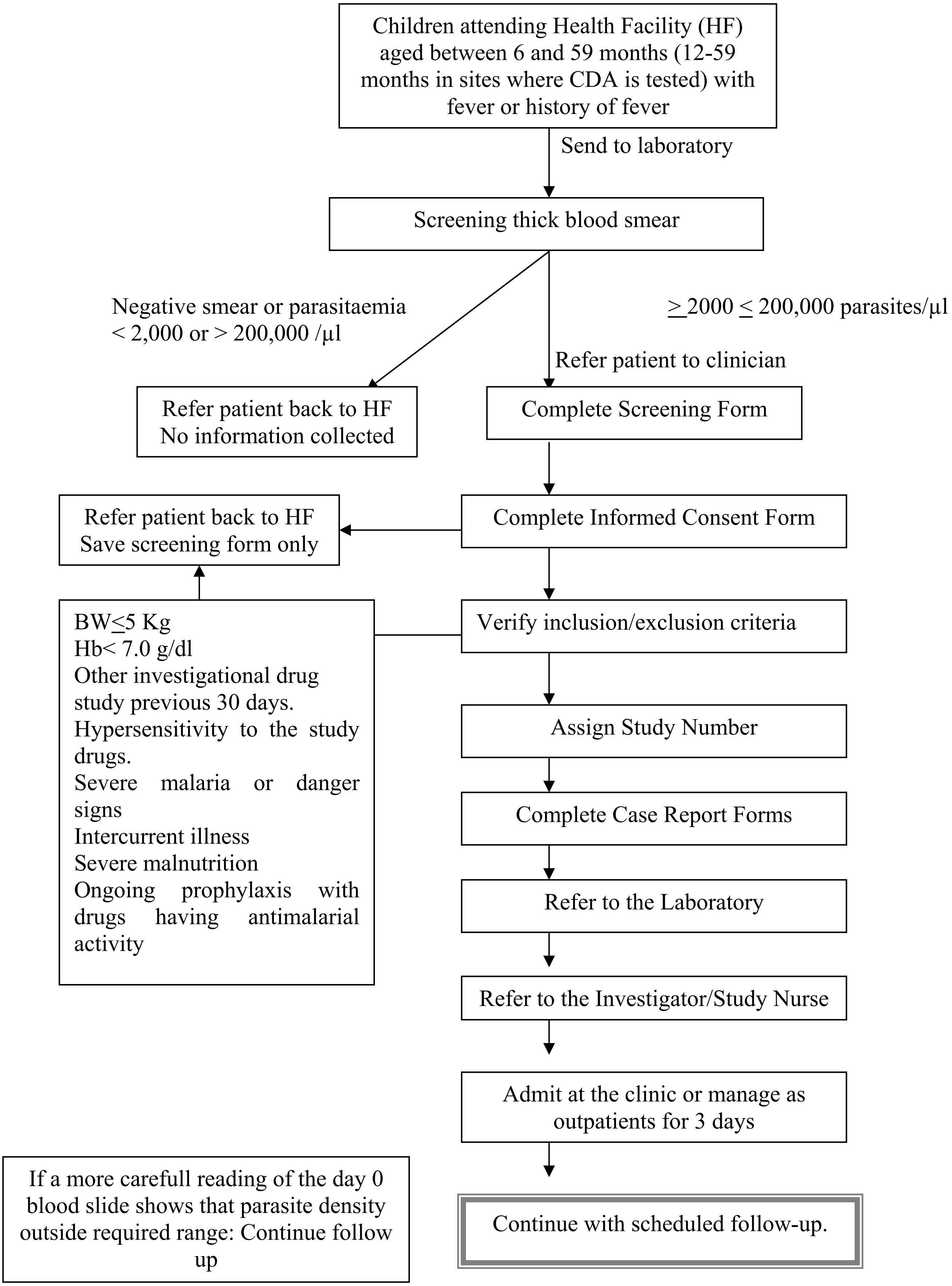

APPENDIX VII. CRITICAL STEPS

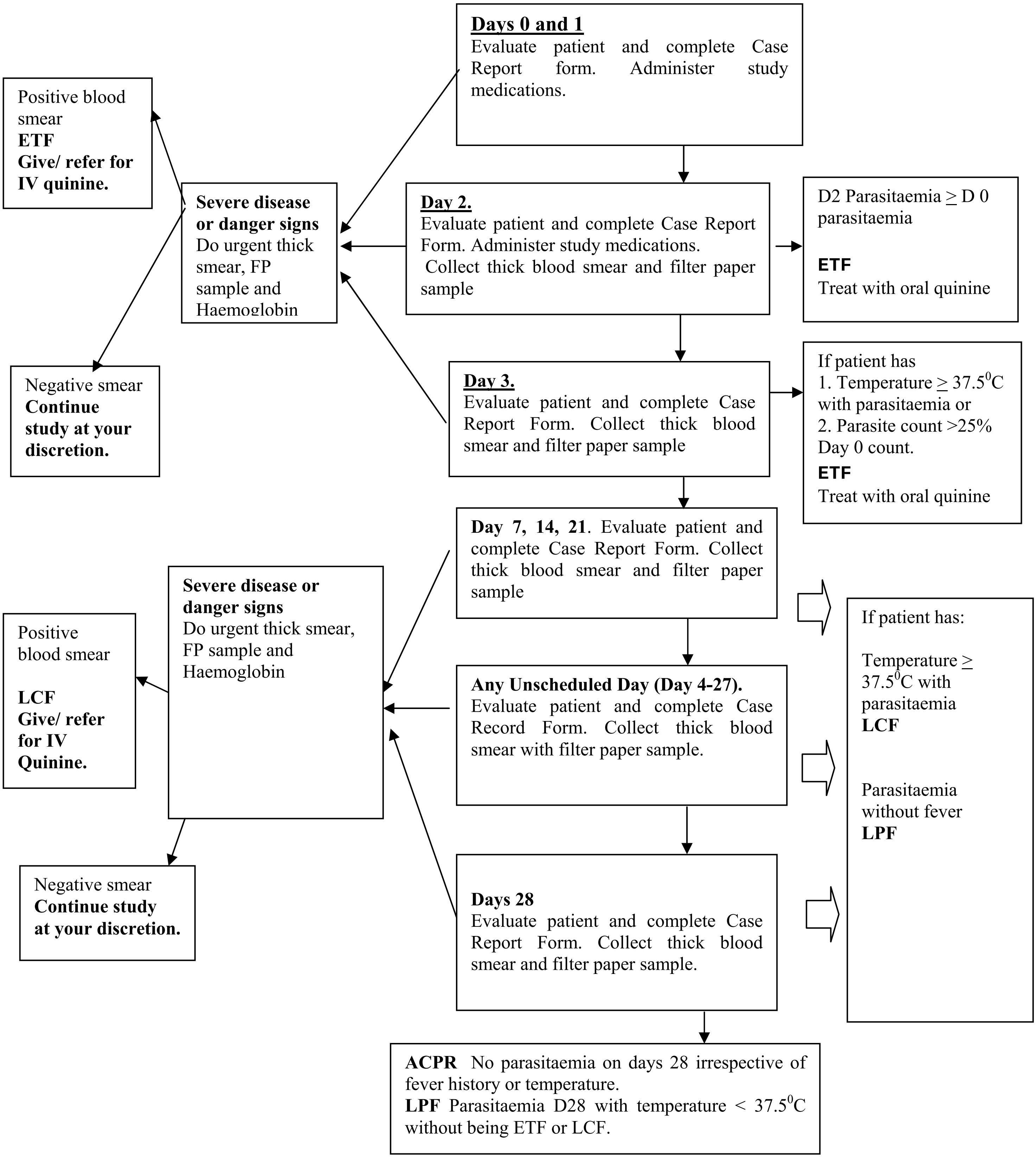

APPENDIX VIII. OVERVIEW OF THE WHOLE STUDY DESIGN

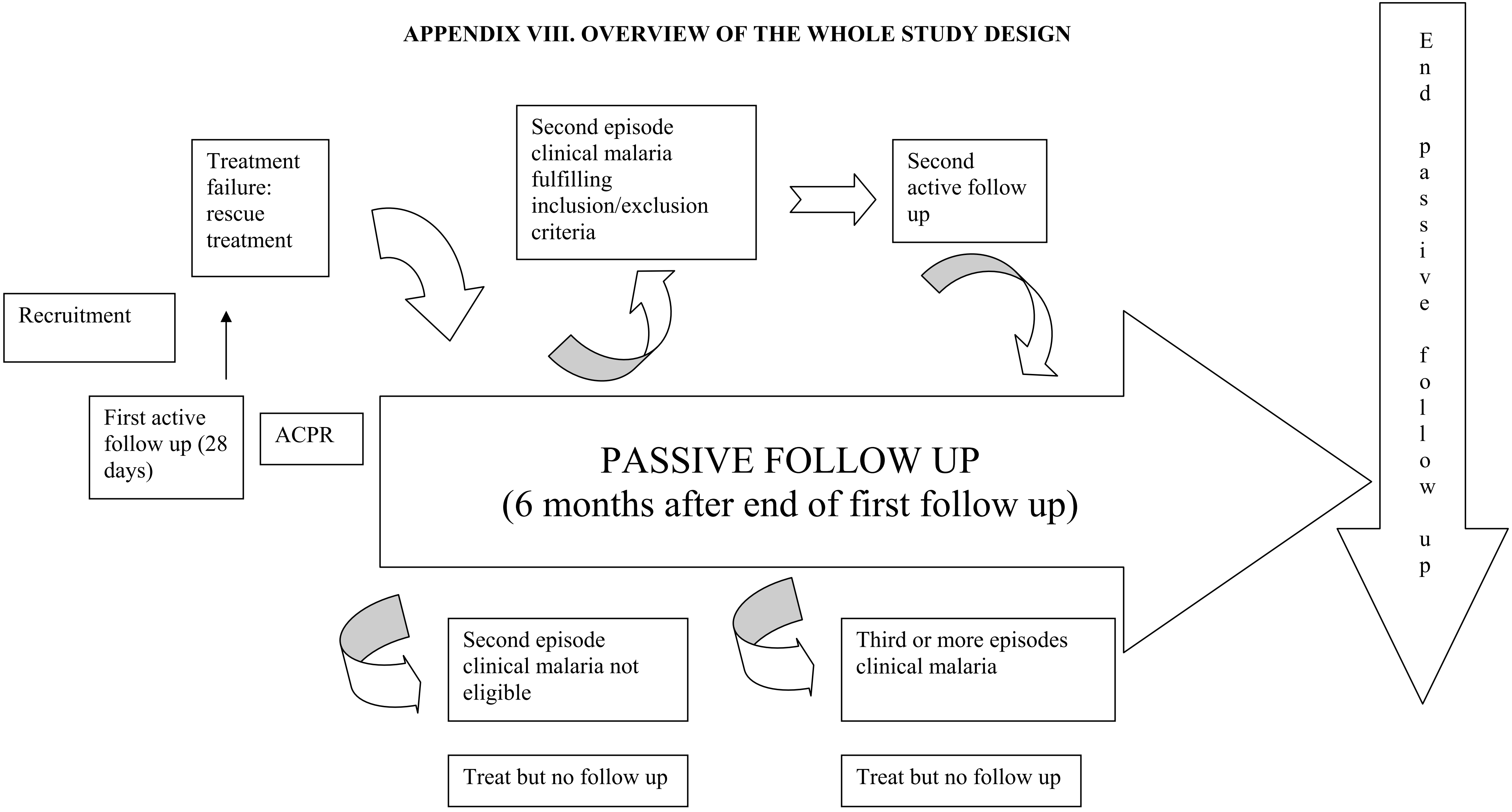

Supplement: Text S1 — Study protocol. (PDF) [file pmed.1001119.s001.pdf]
