# Supplementary material for: A Head-to-Head Comparison of Four Artemisinin-Based Combinations for Treating Uncomplicated Malaria in African Children: A Randomized Trial
Source: PLoS Med. 2011 Nov 8;8(11):e1001119. doi: 10.1371/journal.pmed.1001119 (PMC3210754; doi:10.1371/journal.pmed.1001119)
Supplement: Text S4 — Protocol amendment 2. (PDF) [file pmed.1001119.s004.pdf]

**Protocol No.:**	**Protocol EDCTP-NCT00393679**
**Date:**	April 7th, 2008
**Version:**	Amendment N. 2.0, dated April 7th 2008
**Previous version**	Amendment N.1, dated February 1st 2007
**Title:**	Evaluation of 4 artemisinin-based combinations for treating uncomplicated malaria in African children
**Countries :**	Burkina Faso, Nigeria, Gabon, Uganda, Rwanda, Zambia and Mozambique
**Sites:**	Nanoro (Burkina Faso);<br>Calabar (Nigeria);<br>Lambaréné (Gabon);<br>Mbarara, Jinja and Tororo (Uganda);<br>Rukara and Mashesha (Rwanda);<br>Ndola (Zambia);<br>Manhiça (Mozambique)
**Study drugs**	Dihydroartemisinin-Piperaquine,<br>Amodiaquine-Artesunate,<br>Artemether-Lumefantrine,<br>Chlorproguanil-Dapsone plus Artesunate.<br>The Chlorproguanil-Dapsone plus Artesunate arm was discontinued on 17th February 2008.
**Sponsor:**	Prince Leopold Institute of Tropical Medicine, Antwerp, Belgium
**Co-ordinating Investigator:**	Professor Umberto D'Alessandro
**Address:**	Prince Leopold Institute of Tropical Medicine, Nationalestraat 155, B-2000, Antwerp, Belgium
**Telephone:**	0032 3 247 6354
**Fax:**	0032 3 247 63 59
**Email:**	udalessandro@itg.be

Rationale for the amendment N. 2

The protocol is amended for the second time following the decision of GlaxoSmithKline (GSK) to discontinue the clinical development of the fixed-dose combination Chlorproguanil/ Dapsone/ Artesunate (CDA). Such decision is based on the results of a phase III clinical trial, comparing CDA and Artemether-Lumefantrine (Coartem®) for the treatment of acute, uncomplicated *Plasmodium falciparum* malaria in children. Efficacy was comparable in the two groups; however, there was a significant difference in the haematological safety profile of the compounds. The propensity of CDA to cause clinically significant Haemoglobin (Hb) declines in patients with glucose-6-phosphate dehydrogenase (G6PD) deficiency would preclude its large use in peripheral health facilities, where routine G6PD testing is not available *(see Annex I*).

The discontinuation of CD-A

The decision of GSK led to immediate discontinuation of the CD-A arm, on 17th February 2008, in the present study. The consequences are the following:

- In the sites where CD-A was tested, patients will be randomised into two rather than three treatment arms.
- The lower age limit for inclusion, in the sites where CD-A was tested, will be 6 months instead of 12 months. This is the same lower age limit for inclusion in the other sites not testing CD-A.
- The extra haemoglobin examinations, requested at Day 1 and Day 2 in the sites where CD-A was tested, will be not be required anymore.
- The collection of an additional blood sample for later determination of the G6PD status, requested at Day 0 in the sites where CD-A was tested, will be not be required anymore.
- The children who were treated with CD-A for the first active follow-up, will not be retreated with CD-A, but with the first-line treatment according to the national protocol. Nevertheless, they will be passively followed up as the other children of the study cohort.

The following updated flow-chart, that applies both to the first and second follow up, summarizes the assessments per visit:

Day	0	1	2	3	4	5	6	7 +/-1 day ([6])	14 +/-1 day ([6])	21 +/- 2 days ([6])	28 +/- 2 days ([6])	Any other day[1]
History (symptoms)	X							X	X	X	X	X
Informed consent	X[2]											
Examination (clinical)	X	X	X	X				X	X	X	X	X
Temperature	X	X	X	X				X	X	X	X	X
Blood film	X	X	X	X				X	X	X	X	X
Filter paper PCR	X	X							X	X	X	X
Adverse drug reactions	X	X	X	X	X[4]	X[4]	X[4]	X	X	X	X	X
Concomitant medications	X	X	X	X				X	X	X	X	X
Haematology	X			X				X	X[5]		X	X
Biochemistry	X							X	X[3]		X	
Treatment	X	X	X									

[1] Spontaneous attendance to health facility; [2] Only at the beginning of the first follow up. [3] If abnormal at day 7. [4] If patient attends spontaneously the health facility. [5] Full haematology tests will be performed at visit 14 only if there were abnormalities at visit 7; otherwise, only haemoglobin will be evaluated at visit 14. ([6]) Calendar days (not working days)

The recruitment rate and timelines

The recruitment rate in the different centres was also evaluated. Some centres experience difficulties to get to the foreseen 510 patients/site. The following actions will be undertaken to optimize the overall recruitment;

- The recruitment period will be extended for an additional 6-month period; the end-of-recruitment is now foreseen on December 31st, 2008.
- The sites of Nanoro (three treatment arms) and Tororo (two treatment arms) will recruit 300 additional patients.
- The sites of Jinja, Manhiça and Mbarara will continue recruitment, in order achieve the sample size of 510 patients, but randomised to two instead of three treatment arms (due to the discontinuation of CD-A). The table below shows the likely distribution of patients by site.

Country	Sites	Planned number of patients	Study treatments		
Burkina Faso	Nanoro	810	AQ+AS	DHAPQ	AL
Nigeria	Calabar	510	AQ+AS	DHAPQ	AL
Zambia	Ndola	510	AQ+AS	DHAPQ	AL
Gabon	Lambarené	200	AQ+AS	DHAPQ	AL
Uganda	Mbarara	510	AQ+AS		DHAPQ
Uganda	Jinja	510	DHAPQ		AL
Uganda	Tororo	810	DHAPQ		AL
Rwanda	Rukara	340	DHAPQ		AL
Rwanda	Mashesha	340	DHAPQ		AL
Mozambique	Manhiça	510	AQ+AS		DHAPQ

The original sample size calculation gave n=170/treatment to demonstrate equivalence between two treatments with respect to treatment failure, and each site was powered individually according to this. Clearly one site will not recruit this number, four sites will achieve the desired number and five sites will achieve higher than this. The calculated power to demonstrate equivalence depends on the assumption about the true underlying failure rates.

The assumption of zero difference between true rates for any two treatments made here results in the lowest sample size requirement (n=170/treatment), hence the recruitment of additional patients in some sites allows some robustness to the case when true rates do actually differ by up to 2%. In addition, meta-analysis will be undertaken for each pairwise comparison of treatments, thereby increasing the power.

Hospitalization during the treatment period

Several sites experienced difficulties to comply with the protocol initial requirement concerning the hospitalisation of patients during the first three study days (treatment period).

The main purpose for this requirement was to ensure strict monitoring of the study drug administration; however, it appeared that hospitalization is not mandatory provided that the study medication is administered according to the randomization list under direct observation

by a study nurse or any other research team member not involved in the patient's clinical assessment.

Therefore, the three-day hospitalization is not mandatory anymore, provided that the study medication is administered under direct observation by a study nurse or any other research team member not involved in the patient's clinical assessment.

AMENDMENT SIGNATURE PAGE

Approval	Signature	Date
Prof./Dr. …………………	____________________ Principal Investigator	___/___/___
Prof./Dr. …………………	____________________ Investigator	___/___/___
Prof./Dr. …………………	____________________ Investigator	___/___/___
Prof./Dr. …………………	____________________ Investigator	___/___/___
Prof./Dr. …………………	____________________ Investigator	___/___/___
Prof./Dr. …………………	____________________ Investigator	___/___/___
Prof./Dr. …………………	____________________ Investigator	___/___/___
Prof. Umberto D'Alessandro	[signature] Co-ordinating Investigator	09/04/2008
Prof. Bruno Gryseels	[signature] Prince Leopold Institute of Tropical Medicine Director	11/04/2008

RESEARCH PARTICIPANT INFORMED CONSENT FORM

STUDY TITLE

Evaluation of 4 artemisinin-based combinations for treating uncomplicated malaria in African children

- Multicentre Study In Africa -

Please read the background information and informed consent form carefully. The background information explains your rights and our responsibilities to you. If you have any questions concerning the study please do not hesitate to ask any of the doctors. Before you decide, it is important for you to understand why the research is being done and what it will involve. You will be given a copy of this signed document (Informed Consent Form) to take home with you.

YOU MUST KEEP THIS BACKGROUND INFORMATION WITH YOU THROUGHOUT THE STUDY PERIOD.

PURPOSE OF THE STUDY

This research study is being done to learn more about the treatment of malaria. We are carrying out a research study to compare different medicines for the treatment of mild malaria. The medicines we are studying are: dihydroartemisinin-piperaquine, Artemether-Lumefantrine, and amodiaquine-artesunate. All these drugs are active against malaria. With this study we want to find out their relative value in curing malaria in terms of efficacy and safety. Five thousand and ten patients from seven different African countries will participate in this study. Each country will test *2 or 3* of the medicines mentioned above.

HOW THE STUDY IS DONE

The child under your care will be treated for malaria with one of the above study medicines. After being included in the study, your child will be actively followed for 28 days to see if the malaria infection is completely cured. If your child is not completely cured by the study medicines, s/he will then be given treatment according to the standard practice in your country. The study medicine that your child will receive will be determined by a process of randomization. Randomization means that your child will receive by a study nurse one of the three medicines studied in your country by chance. The treating physician will not know which of the two or three medicines has been given to your child, unless he/she thinks that it is better to know it for giving a better clinical care. You are being asked to allow your child (child under your care in the case of a legal guardian), to participate in this study. Your child will be actively followed up for 28 days or until such time as you or the study doctors decide that your child should no longer participate in the study. Afterwards, you will be asked to attend the health facility for the next 6 months any time your child is sick where s/he will be

examined by the study doctor and treated accordingly. However, if your child has malaria again, s/he will be treated with the same drug used for the previous episode and actively followed up again for the next 28 days after treatment. You can choose to withdraw your consent to participate in the study any time and without influencing the medical attention your child may need.

The study may be discontinued by the sponsor at any time, and for any reason.

PROCEDURES

1) The study doctors will examine your child today.
2) A blood sample will be collected. A small amount of blood will be taken by fingerprick to examine for malaria parasites, to measure the blood count, to store blood samples on filter paper for future laboratory tests that will not impact on the health care of your child.
3) If the diagnosis of malaria is confirmed, and your child is eligible for the study, treatment with either dihydroartemisinin + piperaquine (DHA+PPQ), arthemeter + lumefantrine (A+L), or amodiaquine-artesunate. (AQ+AS) will be given by a study nurse or any other research team member not involved in your child' clinical assessment, at the clinic during the first 3 days. This means that your child can be hospitalised the first 3 days of the study.
4) You will be asked to return to the clinic at least 7 more times over the next month so that the success of the treatment can be judged. At each of the follow-up visits, your child will be examined by the study doctors and, a small amount of blood will taken by fingerprick to examine for malaria parasites and to save on filter paper.
5) If case of missing appointment, the home health visitor will visit your child at your home to find out why you missed the appointment and bring your child to the clinic for assessment.
6) If, at any time, the treatment given to your child does not seem to be working well, it will be changed to treatment according to the usual standard of care.
7) There will be someone at the study clinic every day from 8:00 am to 5:00 pm and at night. You can come to the clinic for evaluation anytime that your child is ill during the next 7 months.
8) For the haematology there will be at least 4 blood samples: one before the first dose at D0, the second one at D3, the third at D7, i.e. one week after D0, the forth at D28, i.e. four weeks after D0. A fifth sample will be taken at D14, i.e. two weeks after D0, and all haematology tests done if the previous exam had shown some abnormal values The amount of blood collected for each sample will be less than 1mL.
9) For biochemistry, 3 samples will be collected: one before the first dose at D0, the second at D7, i.e. one week after D0 and the third at D28, i.e. four weeks after D0. An additional sample will be collected at D14 if the results at D7 are outside the normal ranges. Each sample will be of 1 mL.
10) Blood samples will be collected from an arm vein or from a finger by an experienced nurse. Blood sampling may cause pain and swelling. In addition small violet spots around the site of injection called hematoma could appear.

RISKS AND DISCOMFORTS

1) Side effects following treatment with the study medications could occur. Generally, side

effects (nausea, headache, dizziness…) are expected to be mild and short-lived.

Your child will be monitored closely after receiving treatment for malaria with the study medications for any possible side effects of the drugs and will receive appropriate medical care for any problem that happens during the course of the study.

2) Randomization: Your child will be assigned to a treatment group by chance. The drug will be administered by the study nurse or anther research team member not involved in your child' clinical assessment, and the treating physician will not know which of the three medicines has been given to your child, unless he/she thinks that it is better to know it for giving a better clinical care. The treatment your child receives may prove to be less effective or to have more side effects than the other study treatments or than other available treatments. This will not be known until after the study is completed.
3) Severe malaria: Your child may develop malaria that is severe even after receiving treatment with study medications. If your child shows any evidence of severe malaria (including persistent vomiting, low blood (anaemia), convulsions, confusion, or coma) treatment with the usual standard of care will be given and your child will be referred for possible admission to hospital.
4) Blood draws: The risks of drawing blood from a fingerprick and/or of the venous collection of blood include temporary discomfort from the needle stick, bruising, skin infection, and fainting. The amount of blood removed will be too small to affect your child's health.
5) Unknown Risks: The research treatments may have side effects that no one knows about yet. The researchers will let you know if they learn anything that might make you change your mind about your child's participation in the study.
6) Confidentiality: Participation in research may involve a loss of privacy, but information about your child will be handled as confidentially as possible. Medical information related to malaria will be collected on your child and only people taking care of the child and/or the study personnel will have access to this information. Records will be kept as confidential as possible. You will also have the right to request and see the information collected during this study on your child.

BENEFITS

1) The potential benefit to your child is that the treatment received may prove to be more effective than the other study treatments or than other available treatments, although this cannot be guaranteed.
2) Your child will receive clinical care from the medical officers and nurses of the project staff in the study clinic. This will include care for unscheduled sick visits.
3) The knowledge gained from this study will help your country in determining the best treatment for uncomplicated malaria.

COST/PAYMENT

After enrolment in the study, you will not be charged for clinic visits or treatment. Your child will not be paid for participation in the study. We will reimburse any transport costs incurred for clinic visits and meals will be provided when your child is admitted for observation and treatment administration.

ALTERNATIVES TO PARTICIPATION

Your child's participation in this study is completely voluntary. If you decide that you do not want to participate in the study or decide to withdraw your child from the study at any time and for any reason, this will not affect your child's care at the outpatient department, where standard care for all medical problems is available. During the study, you will be informed promptly of any new information that may influence your willingness to continue participation in the study.

CONSEQUENCES OF WITHDRAWAL

Should you decide to withdraw your child from the study before your child has finished the course of study medicines, then your child will receive the local standard treatment for malaria from the study team, but after the standard treatment has been given, medical care will no longer be provided by the study team. If the child is withdrawn from the study after completion of the course of study medicines, then no further care will be provided by the study team.

USE OF THE RESULTS

The findings from this study may be published in a medical journal. The study participants will not be identified by name. After the study is completed, you may request an explanation of the study results.

TREATMENT AND COMPENSATION FOR INJURY

If you are injured or have questions about injuries as a result of being in the study, please contact the doctors in the study clinic. The services at the public health facility will be open to you in case of any such injury.

VOLUNTARY PARTICIPATION

Participation in this study is entirely voluntary. You have the right to refuse your child's participation or to withdraw at any point in this study without negative consequences or loss of benefits to which you and your child are otherwise entitled.

Implication of your SIGNATURE OR THUMBPRINT

If you give consent for your child to participate in this study, you should sign or place your thumbprint in the consent form. Your signature or thumbprint below means that you understand the information given to you about your child's participation in the study and in the consent form. You will also be asked to sign another copy of this informed consent form for documentation.

CONSENT FORM

CONSENT FORM FOR PARTICIPATION IN RESEARCH PROJECTS AND CLINICAL TRIALS

Study Title

Evaluation of 4 artemisinin-based combinations for treating uncomplicated malaria in African children

- Multicentre Study In Africa -

Local Investigator: ____

Address:____

Contact number____

I, ... mother/father/legal representative declare that I have understood the objectives and purposes of this study. I agree that my child... participates in this study.

I am aware that I can withdraw my child from the study at any time without any consequence to my child or to me.

____

Name of parent/legal representative

____

Signature or Thumbprint * of parent/ legal representative   Date/Time

____

Name and role of the person obtaining the consent

____

Signature of person obtaining consent   Date/Time

*If the parent or guardian is unable to read and/or write, an impartial witness should be present during the informed consent discussion. After the written informed consent form is read and explained to the parent or guardian, and after they have orally consented to their child's participation in the trial, and have either signed the consent form or provided their fingerprint, the witness should sign and personally date the consent form. By signing the consent form, the witness attests that the information in the consent form and any other written information was accurately explained to, and apparently understood by, the parent or guardian, and that informed consent was freely given by the parent or guardian.

Name of Person Witnessing Consent (printed)

Signature of Person Witnessing Consent   Date/Time
